# Site-selective radical reactions of kinetically stable open-shell singlet diradicaloid difluorenoheteroles with tributyltin hydride and azo-based radical initiators†

**DOI:** 10.1039/d3sc00381g

**Published:** 2023-05-02

**Authors:** Naoki Tabata, Takumi Uchino, Chitoshi Kitamura, Kazunari Yoshizawa, Yoshihito Shiota, Shin-ichiro Kato

**Affiliations:** a Department of Materials Chemistry, School of Engineering, The University of Shiga Prefecture 2500 Hassaka-cho Hikone Shiga 522-8533 Japan kato.s@mat.usp.ac.jp; b Institute for Materials Chemistry and Engineering (IMCE), Kyushu University 744 Motooka Nishi-ku Fukuoka 819-0395 Japan shiota@ms.ifoc.kyushu-u.ac.jp

## Abstract

We have demonstrated site-selective radical reactions of the kinetically stable open-shell singlet diradicaloids difluoreno[3,4-*b*:4′,3′-*d*]thiophene (DFTh) and difluoreno[3,4-*b*:4′,3′-*d*]furan (DFFu) with tributyltin hydride (HSn(*n*-Bu)_3_) and azo-based radical initiators. Treatment of these diradicaloids with HSn(*n*-Bu)_3_ induces hydrogenation at the *ipso*-carbon in the five-membered rings, while treatment with 2,2′-azobis(isobutyronitrile) (AIBN) induces substitution at the carbon atoms in the peripheral six-membered rings. We have also developed one-pot substitution/hydrogenation reactions of DFTh/DFFu with various azo-based radical initiators and HSn(*n*-Bu)_3_. The resulting products can be converted into substituted DFTh/DFFu derivatives *via* dehydrogenation. Theoretical calculations unveiled a detailed mechanism of the radical reactions of DFTh/DFFu with HSn(*n*-Bu)_3_ and with AIBN, and that the site-selectivity of these radical reactions is controlled by the balance of the spin density and the steric hindrance in DFTh/DFFu.

## Abstract 2

Kinetically stable open-shell singlet diradicaloids regioselectively react with radical species generated from azo-based radical initiators as well as HSn(*n*-Bu)_3_.

## Graphical abstract

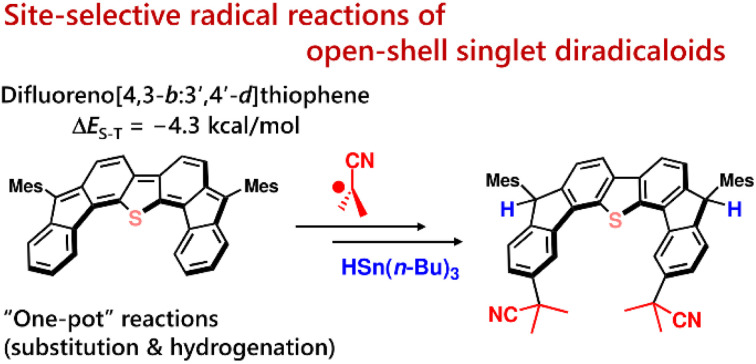

## Introduction

Open-shell singlet diradicaloids with a polycyclic π-conjugated backbone have attracted great attention due to their unique optoelectronic and magnetic properties.^1–7^ Scaffold moieties such as *ortho*- and *para*-quinodimethane (*o*-QDM and *p*-QDM, respectively), 2,6- and 1,5-naphtoquinodimethane (2,6-NQDM and 1,5-NQDM, respectively), and their π-extended derivatives play critical roles in inducing molecules to exhibit singlet diradical character.^8^ According to *Clar's aromatic sextet* rule, the benzenoid form of the open-shell contributor with two distinct radical centers is stabilized by an aromatic sextet. Installing bulky substituents into the aforementioned pro-aromatic quinoidal scaffolds has been a popular approach to synthesizing chemically and thermally stable open-shell polycyclic π-conjugated diradicaloids (PCDs).^9,10^ Elucidation of the inherent reactivity of PCDs is indispensable not only for understanding their diradical character,^11–18^ but also for the development of late-stage modifications^19–21^ and chemical transformations of PCDs into structurally and electronically interesting compounds that are difficult to access from the corresponding closed-shell compounds.^22–31^

**Fig. 1:**
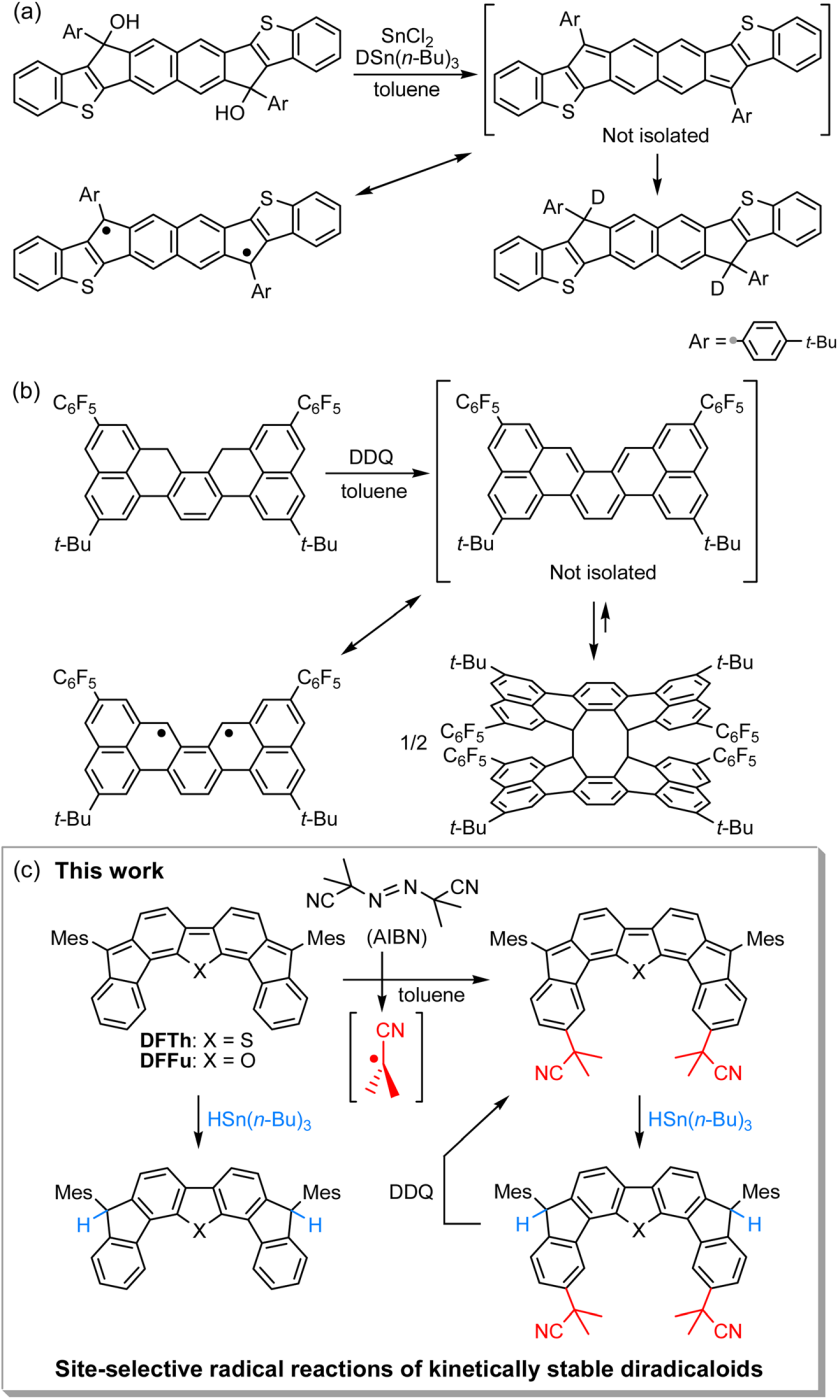
Reactions of recently reported kinetically unstable diradicaloids, in which (a) 2,6-NQDM or (b) *o*-QDM structures are embedded. (c) Site-selective radical reactions of difluoreno[4,3-*b*:3′,4′-*d*]thiophene (DFTh) and difluoreno[4,3-*b*:3′,4′-*d*]thiophene (DFFu; Mes = 2,4,6-trimethylphenyl) with AIBN and/or HSn(*n*-Bu)_3_, as well as dehydrogenation of the products. The resonance between the closed- and open-shell diradical structures of DFTh is shown in Fig. 3.

Unique reactivity that originates from diradical character has been sporadically reported in kinetically unstable PCDs without bulky substituents.^32–37^ For example, Haley's group has described the reaction of an *in situ*-formed PCD that possesses a 2,6-NQDM scaffold with the deuterium source tributyltin deuteride (DSn(*n*-Bu)_3_) at the *ipso*-carbons in the five-membered rings, which proceeded *via* a radical mechanism (Fig. 1a).^34^ More recently, Kubo's group has reported that a thermodynamically stable but kinetically unstable PCD that possesses an *o*-QDM scaffold undergoes dimerization *via* the [4 + 4] cycloaddition, which occurs through the radical stepwise mechanism (Fig. 1b);^36^ the σ-bonded dimer dissociates into a monomer upon heating or photoirradiation. A similar cyclodimerization, as well as the cyclotrimerization and higher oligomerization of a trimethylsilylethynyl-substituted indeno[1,2-*b*]fluorene derivative that incorporates a *p*-QDM scaffold, have previously been reported by Zhao's group despite the major closed-shell feature of the ground state (Scheme S1 in the ESI†).^32^ These reactions are rare examples that unveil the diradical contribution in the electronic ground state of PCDs from the viewpoint of their peculiar radical reactivity. It might, however, be argued that such radical reactions are found due to a lack of kinetic stability; in other words, in the stable PCDs synthesized so far, this unique reactivity is possibly masked due to the efficient kinetic protection. Moreover, kinetically stable PCDs usually involve relatively laborious synthetic routes, which may dissuade chemists from investigating their reactivity. Accordingly, the exploration of the reactivity of stable PCDs has been very limited to date and remains a challenging task.^19–22,28^

We have recently designed and synthesized difluoreno[4,3-*b*:3′,4′-*d*]thiophene (DFTh) and difluoreno[4,3-*b*:3′,4′-*d*]furan (DFFu), which contain Tschitschibabin's hydrocarbon as a structural motif, and examined their diradical activity and functions (Fig. 1c; the open-shell contributors are shown in Fig. 3).^38–40^DFTh and DFFu are stable enough to be handled under ambient conditions without any special precautions due to the bulky Mes groups. They exhibit distinct diradical character with singlet–triplet energy gaps (Δ*E*_S–T_) of −4.3 and −4.9 kcal mol^−1^ for DFTh and DFFu, respectively, which is reflected in their narrow optical/electrochemical gaps and ambipolar charge-transport behavior in organic field-effect transistor devices. Advantageously, these PCDs can be obtained in quantities exceeding 500 mg in a single sequence from commercially available materials (over five steps). The easy accessibility of DFTh and DFFu motivated us to explore their reactivity. Herein, we report that these PCDs react site-selectively with tributyltin hydride (HSn(*n*-Bu)_3_) and azo-based radical initiators such as 2,2′-azobis(isobutyronitrile) (AIBN) (Fig. 1c). Computational studies clearly demonstrated that DFTh/DFFu behave as π-diradicaloids in terms of reactivity; the site-selectivity observed in the reactions can be interpreted in terms of a balance between the diradical activity and the steric hindrance. We have developed a one-pot reaction of our PCDs with azo-based radical initiators and HSn(*n*-Bu)_3_; the products can be readily converted into substituted PCDs by oxidative dehydrogenation. This is, to the best of our knowledge, the first example of the synthesis of substituted diradicaloids through chemical transformations of the parent diradicaloids.

## Results and discussion

### Reaction of DFTh/DFFu with tributyltin hydride – radical hydrogenation

**Scheme 1:**
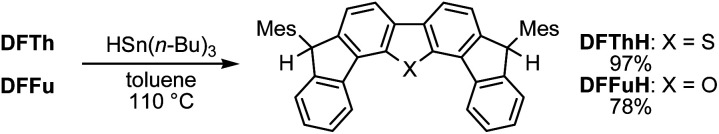
Hydrogenation of DFTh and DFFu with HSn(*n*-Bu)_3_ in toluene.

Initially, inspired by the radical hydrogenation of the PCD reported by Haley and co-workers (Fig. 1a),^34^ we investigated the hydrogenation of DFTh and DFFu (Scheme 1). Treating DFTh and DFFu with an excess of HSn(*n*-Bu)_3_ (10 equiv.) in toluene at 110 °C afforded the corresponding hydrogenated products DFThH (97%) and DFFuH (78%) as ∼1 : 1 diastereomeric mixtures. We then conducted a competitive-reaction experiment to investigate the reactivity of DFTh and DFFu in the hydrogenation. Upon heating a toluene-*d*_8_ solution of DFTh and DFFu (1 : 1) with HSn(*n*-Bu)_3_ at 90 °C for 0.5 h (for details, see the ESI†), DFTh was almost completely consumed, while ∼20% of the DFFu remained based on the ^1^H NMR spectrum (Fig. S1†). This finding suggests that DFTh is more reactive than DFFu, which might reflect the slightly smaller Δ*E*_S–T_ gap, *i.e.*, a higher diradical activity of the former compared to that of the latter.

### Mechanistic insight into the hydrogenation of DFTh/DFFu using DFT calculations

**Fig. 2:**
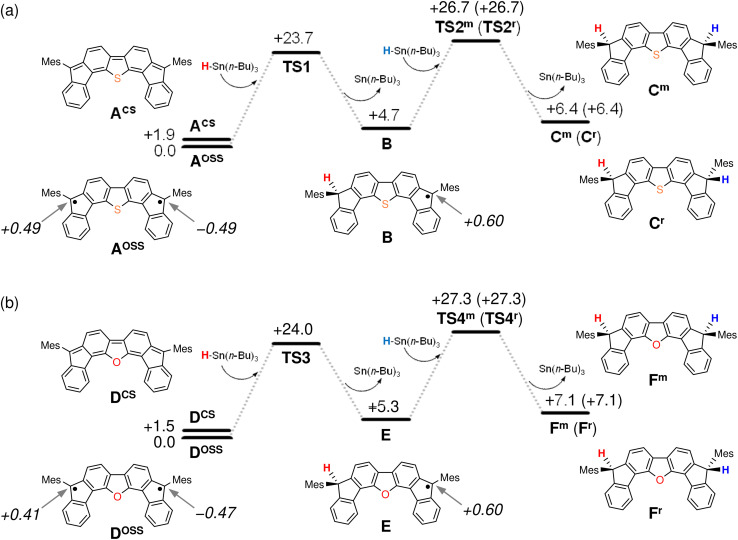
Potential energy diagrams for the hydrogenation of (a) DFTh and (b) DFFu calculated at the B3LYP/6-31G** level. Relative energies are given in kcal mol^−1^, and values in italics indicate spin density.

**Fig. 3:**
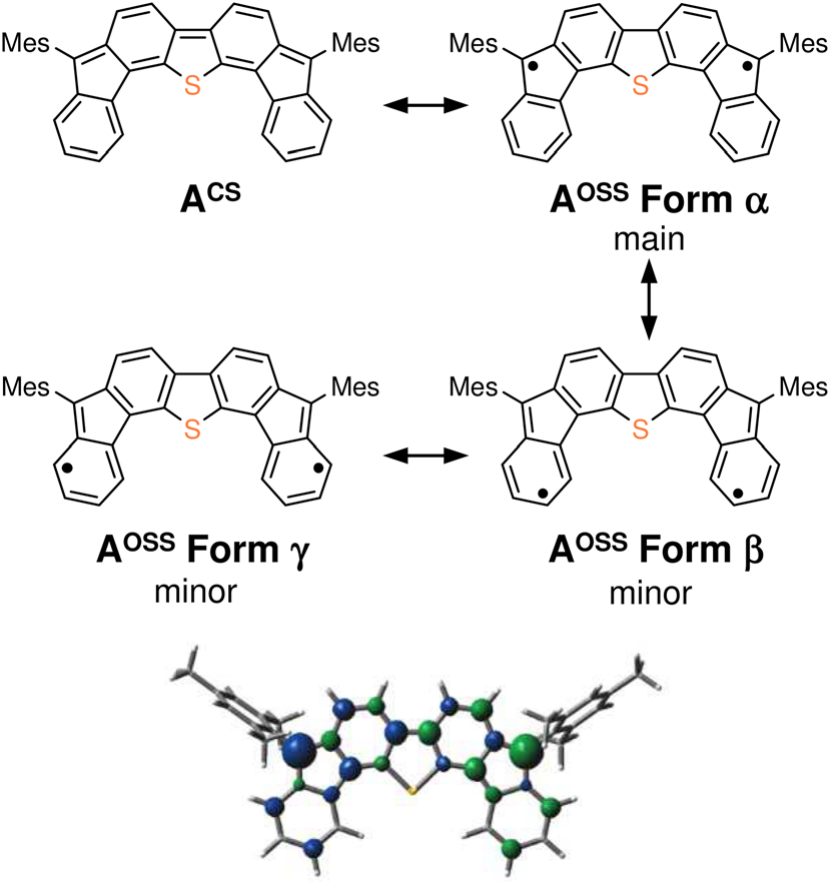
Kekulé resonance structures of A^CS^ and A^OSS^, as well as the optimized structure of A^OSS^ showing the spin density. In the spin density plot, blue is positive and green is negative (isovalue: 0.005).

The results of our DFT calculations at the B3LYP/6-31G** level suggest three forms of DFTh (A), corresponding to the closed-shell singlet state (CS), the open-shell singlet state (OSS), and the triplet state (T) (Fig. 2a)^41^ with calculated relative energies of +1.9 kcal mol^−1^ (A^CS^), 0.0 kcal mol^−1^ (A^OSS^), and +4.3 kcal mol^−1^ (A^T^). Of the three forms, the open-shell singlet is the ground state, which is diradical in nature and engages with HSn(*n*-Bu)_3_ in a hydrogenation. The calculated spin density in Fig. 3 clearly indicates a diradical state of A^OSS^, with contributions from the main form α, the minor form β, and the minor form γ.

In Fig. 2a, the hydrogenation of DFTh proceeds *via* a two-step reaction with two transition states (TS1 and TS2) and one intermediate (B). The first H-atom migration occurs at a transition state with an activation energy of +23.7 kcal mol^−1^, resulting in the formation of a radical intermediate. The hydrogenation occurs at the *ipso*-carbon of one of the five-membered rings, which exhibits the highest spin density (+0.49) in the carbon framework (Fig. 2a). These results lead to the conclusion that the regioselective hydrogenation of DFTh is controlled by the diradical activity of the substrate, *i.e.*, form α of DFTh (Fig. 3) is responsible for the hydrogenation with HSn(*n*-Bu)_3_. In the next step, the hydrogenation of radical intermediate B yields two types of products, *i.e.*, a *meso* compound and a racemate. The activation energies of TS2^m^ and TS2^r^, which correspond to the reactions to give the *meso* and racemic products (C^m^ and C^r^), respectively, are both +22.0 kcal mol^−1^. Moreover, the calculated energies of C^m^ and C^r^ are also identical (+1.7 kcal mol^−1^). These computed results are consistent with the experimental observation that the ratio between the *meso* compound and the racemate is ∼1 : 1.

We next considered the hydrogenation of DFFu, as well as that of DFTh, as shown in Fig. 2b. In the initial structure (D) of DFFu, the ground state is the open-shell singlet state; the relative energies of the closed-shell singlet and the triplet state are +1.5 and +4.9 kcal mol^−1^, respectively. These results indicate that DFFu exhibits a diradical activity similar to that of DFTh and therefore undergoes hydrogenation. This reaction pathway is also a two-step reaction that involves two transition states (TS3 and TS4) and one radical intermediate (E). The reaction is initiated by the transfer of an H atom from D with an activation energy of +24.0 kcal mol^−1^. The activation energy of TS3 for DFFu is slightly higher (0.3 kcal mol^−1^) than that of TS1 for DFTh, which likely corresponds to the result of the aforementioned competitive-reaction experiment. The next step is the hydrogenation of the radical intermediate (E), which results in the formation of the *meso* compound and the racemate (F^m^ and F^r^). The activation energies of TS4^m^ and TS4^r^, which correspond to the reactions to give the *meso* and racemic products, respectively, were both calculated to be +22.0 kcal mol^−1^; this value is identical to the activation energies of TS2^m^ and TS2^r^. The DFT calculations thus suggest that the hydrogenation of DFFu produces equal amounts of the *meso* compound and the racemate, as is the case with the hydrogenation of DFTh.

### Reaction of DFTh with 2,2′-azobis(isobutyronitrile) – radical coupling and hydrogen abstraction

**Scheme 2:**
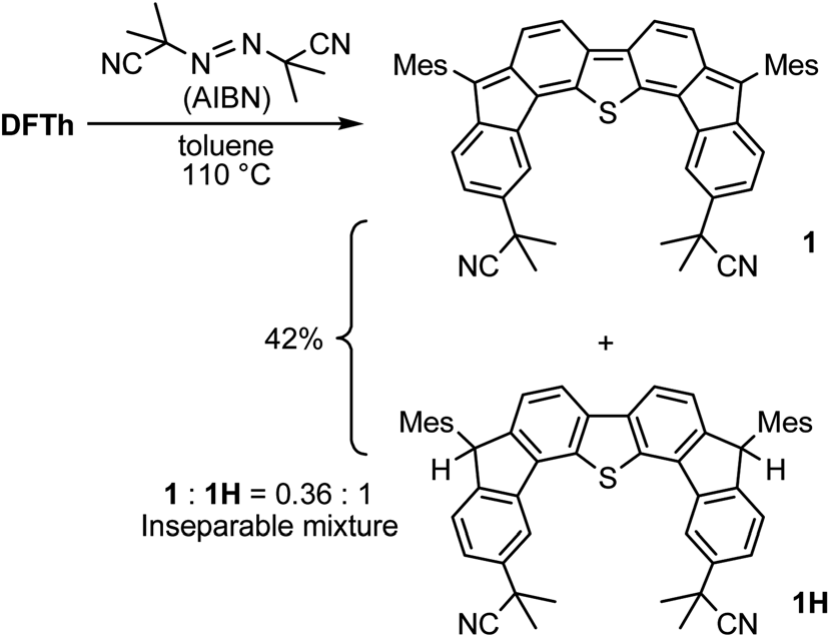
Reaction of DFTh with 2,2′-azobis(isobutyronitrile) (AIBN) and hydrogenation of the substituted product.

We chose AIBN for the reaction with DFTh, given that it is a representative azo-based radical initiator. We envisaged that the reaction between DFTh and the cyanoisopropyl radical (CIPR), which is formed upon heating AIBN, would occur in a site-selective manner; the sterically demanding CIPR can be expected to react at a carbon atom other than the *ipso*-carbons of the five-membered rings due to the steric hindrance between the CIPR and the mesityl group. Gratifyingly, treatment of DFTh with AIBN in toluene at 110 °C furnished 1 and 1H in 42% total yield as an inseparable 0.36 : 1 mixture within a few minutes (Scheme 2 and Fig. S2†). Notably, no regioisomers of 1 or 1H were detected. When the reaction time was prolonged, 1 almost disappeared, and 1H was obtained together with unidentified byproducts. No reaction was observed between pure 1H, which was prepared according to our reported procedures,^39^ and AIBN in toluene at 110 °C. These findings suggest that 1 is formed initially by the radical reaction between DFTh and CIPR, and subsequently hydrogenated to afford 1H.^42^ The use of benzene-*d*_6_ and toluene-*d*_8_ as reaction solvents also provided 1H, which suggests that H-atom transfer from CIPR to 1 affords 1H (Scheme S2†). The molecular structure of 1H was determined unambiguously *via* single-crystal X-ray diffraction analysis (Fig. S3†).

### Mechanistic insight into the reaction of DFTh/DFFu with AIBN using DFT calculations

**Fig. 4:**
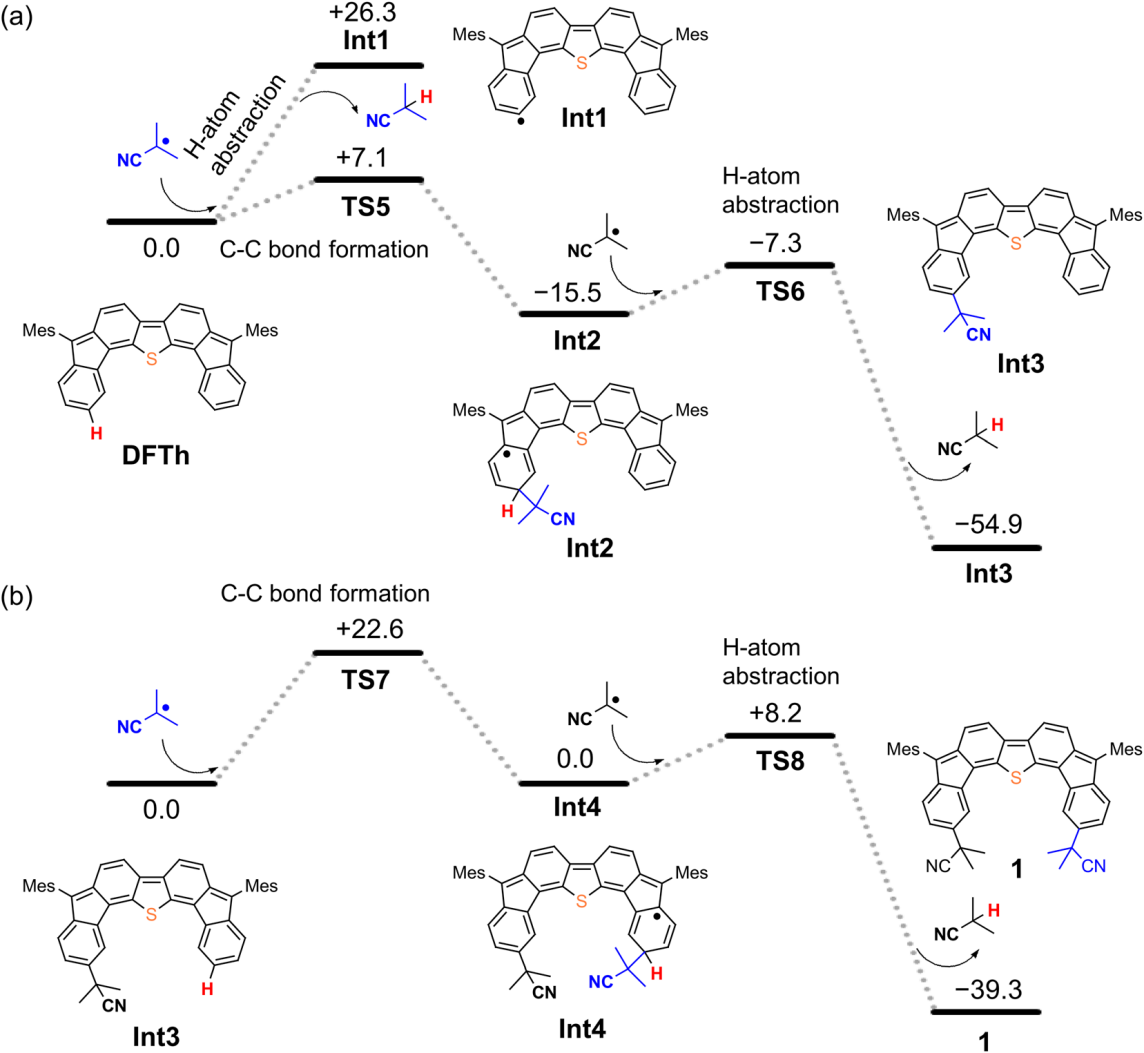
Potential energy diagrams for (a) the first substitution and (b) the second substitution of DFTh, calculated at the B3LYP/6-31G** level; units are given in kcal mol^−1^.

We next considered the reaction of DFTh with AIBN to form a substituted product, as shown in Fig. 4. The decomposition of AIBN produces two CIPRs, which could induce either H-atom abstraction or radical C–C coupling. Thus, there are two possible reactions for the formation of 1 (Fig. 4a); one is the H-atom abstraction by CIPR, and the other is the C–C bond formation between CIPR and the carbon atom of the aromatic ring. H-atom abstraction from DFTh gives radical intermediate Int1 with an energy of +26.3 kcal mol^−1^. The C–C bond formation by radicals involves transition state TS5, which has an activation energy of +7.1 kcal mol^−1^ in the doublet state. Subsequently, Int2 with an energy of −15.5 kcal mol^−1^ in the doublet state is formed. These computational results indicate that the C–C bond formation is energetically favored over the H-atom abstraction in the first step. Thus, we can rule out the formation of Int1*via* direct H-atom abstraction from DFTh. In the next step, another CIPR reacts with Int2, resulting in the formation of Int3, which corresponds to monosubstituted DFTh.^43,44^ This process involves transition state TS6, in which the H-atom abstraction occurs with an activation energy of +8.2 kcal mol^−1^ in the open-shell singlet state, and therefore, this reaction is likely to proceed. Our DFT calculations furthermore suggest that the first substitution reaction is exothermic by 55 kcal mol^−1^ and that the transition state is low enough for the reaction to occur very easily.

As shown in Fig. 4b, the second substitution occurs *via* the same mechanism as the first substitution. The activation energy for the C–C bond formation in the first step of the second substitution *via* transition state TS7 requires +22.6 kcal mol^−1^. In the second step, H-atom abstraction occurs at TS8 with an activation energy of 8.2 kcal mol^−1^. These results suggest that the C–C bond formation in the second substitution is the rate-determining step. The CIPR plays a role in the formation of C–C bonds and the desorption of H atoms in the substitution reaction. The difference in activation energy between the first and the second C–C bond formation (+7.1 *vs.* +22.6 kcal mol^−1^) is quite large, whereas that between the first and the second H-atom abstraction is comparable (+8.2 kcal mol^−1^). An increase in the activation energy of the second C–C bond formation is likely due to the change in the electronic structure of the DFTh π-system resulting from the first cyanoisopropyl substitution. Based on the hydrogenation of DFTh, one might expect that the CIPRs would also react with the carbon atom at the highest spin density, as was observed in the hydrogenation with HSn(*n*-Bu)_3_. However, steric effects are also important for bulky radicals. In fact, the relative energy of the intermediate corresponding to the C–C bond formation at the *ipso*-carbons in the five-membered rings was calculated to be +9.7 kcal mol^−1^ at the B3LYP level. Therefore, we can assume that no such intermediate is present in the reaction with AIBN. As mentioned above, the reaction of DFTh with AIBN gives 1 and 1H. DFT calculations also suggest that the reaction between DFTh and CIPR is controlled by the steric hindrance rather than the diradical activity of DFTh. Since the major Form α of A^oss^ cannot contribute to the reaction due to steric hindrance, minor Form β plays an essential role in the substitution reaction.

### One-pot reaction of DFTh/DFFu with azo-based radical initiators and HSn(*n*-Bu)_3_

**Scheme 3:**
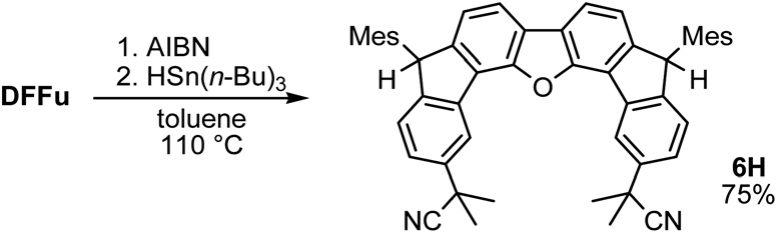
One-pot substitution and hydrogenation of DFFu with AIBN and tributyltin hydride.

A prolonged reaction time of *ca.* 1 h for the reaction of DFTh with AIBN increased the amount of polar byproducts according to a TLC analysis, which renders the purification of 1H laborious. We therefore designed a one-pot reaction of DFTh with AIBN and HSn(*n*-Bu)_3_ (Table 1, entry 1); we expected that such a reaction could converge the product to 1H at a shorter reaction time and suppress the formation of byproducts. After DFTh was allowed to react with AIBN (5 equiv.) in toluene at 110 °C for 20 min, an excess of HSn(*n*-Bu)_3_ (10 equiv.) was added at once, which changed the color of the reaction solution from blue-green to pale yellow within 30 min. Fortunately, 1H was easily purified using column chromatography on silica gel and isolated in 65% yield. We then treated DFTh with various azo-based radical initiators under the one-pot conditions to clarify the reaction scope and limitations (Table 1). The use of 2,2′-azobis(2-methylpropionate) (V-601™), in which the cyano group of AIBN is replaced by a methoxycarbonyl group, and HSn(*n*-Bu)_3_ provided 2H in 51% yield after chromatographic separation of the reaction mixture (entry 2). The use of sterically more demanding 1,1′-azobis(cyclohexane-1-carbonitrile) (V-40™) and dimethyl 1,1′-azobis(1-cyclohexanecarboxylate) (VE-073™) also afforded 3H in 94% yield and 4H in 68% yield, respectively (entries 3 and 4). Decreasing the stoichiometry of AIBN from 5 equiv. to 1.5 equiv. provided monosubstituted 5H in 47% yield as the main product (entry 5). The one-pot reaction using AIBN and HSn(*n*-Bu)_3_ was also applicable to DFFu (Scheme 3), which furnished the disubstituted, hydrogenated product 6H in 75% yield. The reaction did not proceed for 2,2′-azobis(2,4,4-trimethylpentane) (VE-110™), not even after elevating the temperature to 140 °C in mesitylene (entry 6). The reaction with 2,2′-azobis(4-methoxy-2,4-dimethylvaleronitrile) (V-70™) gave a complex product mixture (entry 7).

**Table 1:** One-pot substitution and hydrogenation of DFTh with various azo-based radical initiators and tributyltin hydride a

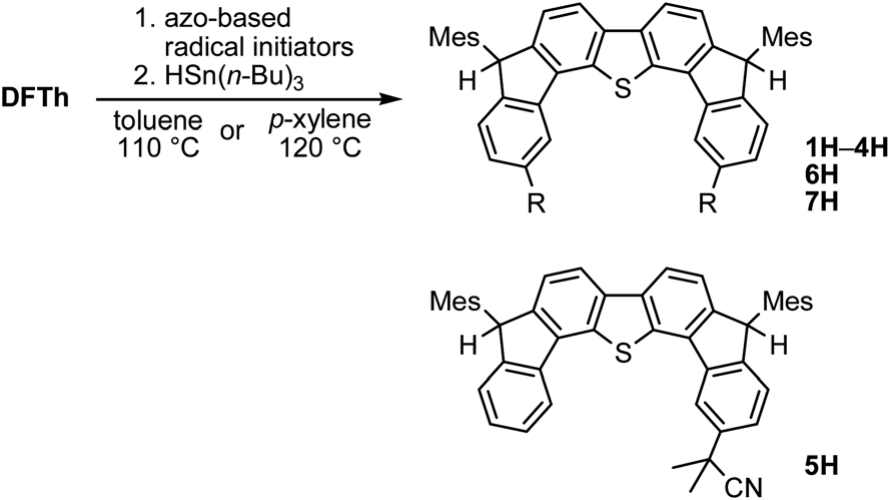				
Entry	Azo-based radical initiator	Product	R	Yield [%]
1	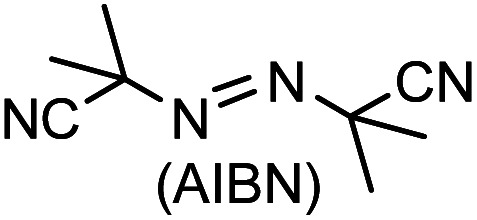	1H	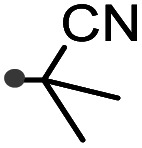	65
2	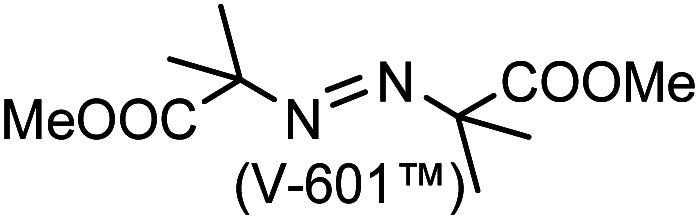	2H	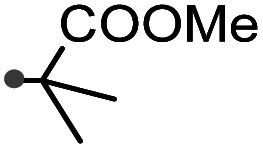	51
3	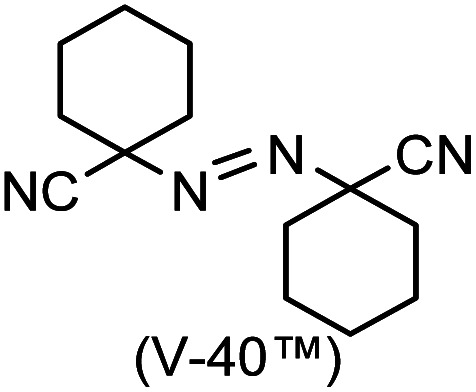	3H	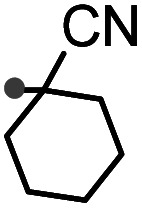	94
4	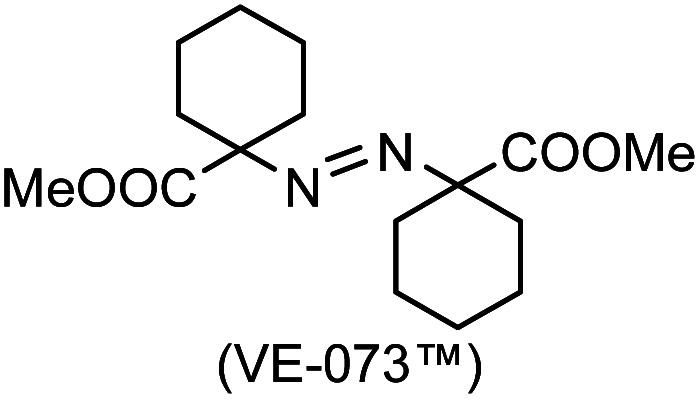	4H	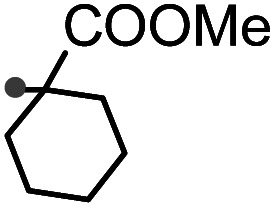	68
5^b^	AIBN	5H		47
6	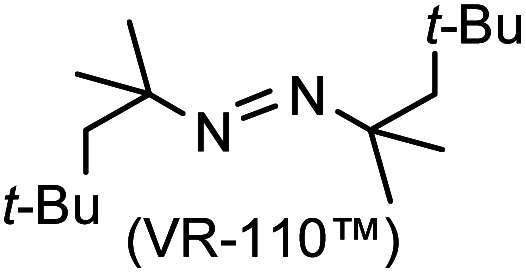	7H	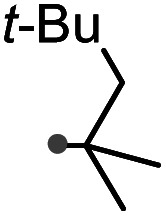	N/A^c^
7	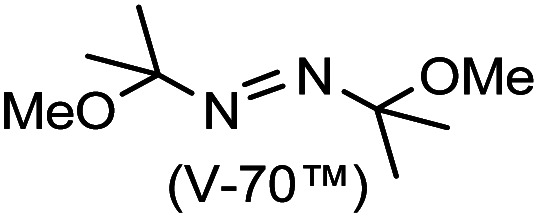	8H	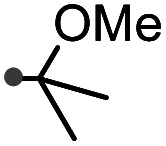	N/A^d^

a Azo-based radical initiator (5 equiv.) and HSn(*n*-Bu)_3_ (10 equiv.).

b AIBN (1.5 equiv.) and HSn(*n*-Bu)_3_ (5 equiv.).

c No reaction between DFTh and VR-110 was observed in mesitylene at 140 °C.

d The reaction between DFTh and V-70 gave a complex product mixture.

### Oxidative dehydrogenation of 1H–6H – synthesis of substituted difluorenoheteroles

**Scheme 4:**
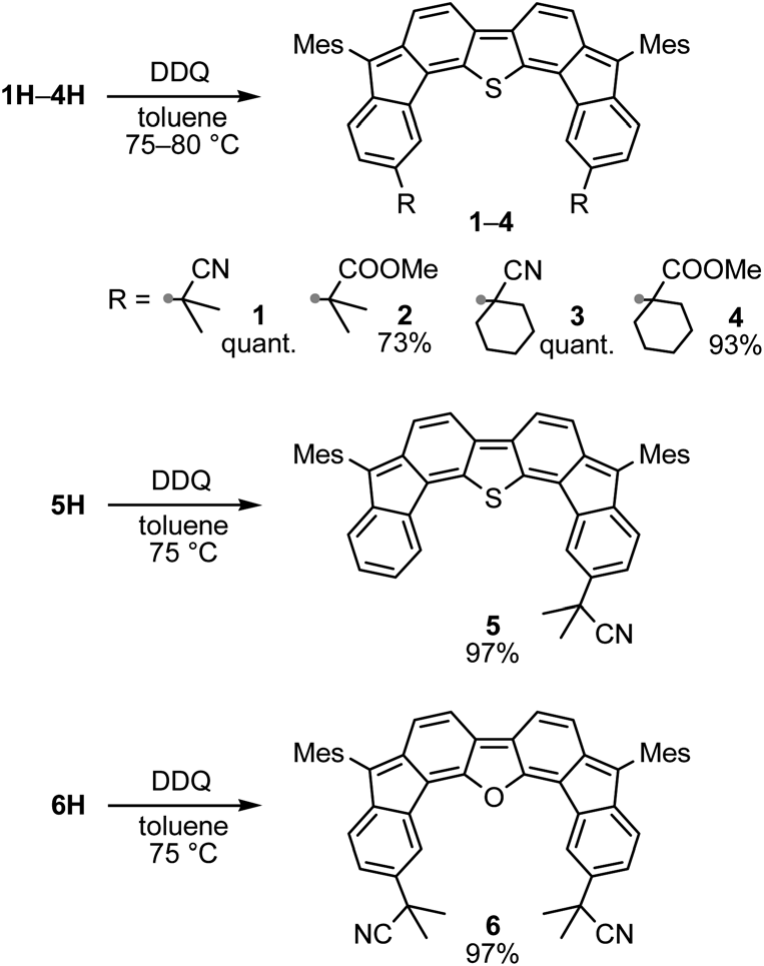
Oxidation of hydrogenated DFTh and DFFu derivatives 1H–6H with DDQ.

We then decided to convert 1H–6H into substituted DFTh and DFFu derivatives *via* oxidative dehydrogenation. Treatment of 1H–6H with 2,3-dichloro-5,6-dicyano-*p*-benzoquinone (DDQ) in toluene afforded the corresponding products (1–6) as deep-blue solids in good yield (Scheme 4). Similar to DFTh and DFFu, 1–6 were sufficiently stable under ambient conditions and could be handled without any special precautions.

Substituted difluorenoheteroles 1–6 are open-shell singlet diradicaloids from the parental DFTh and DFFu. The ^1^H NMR spectra of 1 and 6 at 25 °C in *p*-xylene-*d*_10_ display sharp signals in the aromatic region. Upon increasing the temperature to 110 °C, the aromatic resonances broaden (Fig. S5†). The signals are recovered to full height when the samples are cooled to 25 °C, which indicates that a thermally accessible triplet state is responsible for the signal broadening. The onset of peak broadening, implying population of the paramagnetic triplet state, is observed at ∼50 °C for 1 and ∼70 °C for 6, which are almost identical to those of DFTh and DFFu, respectively. Using the Yamaguchi method, the singlet diradical indices (*y*) of 1′, 6′, DFTh′, and DFFu′, in which the Mes groups of 1, 6, DFTh, and DFFu are replaced with protons, were estimated *via* DFT calculations at the LC-BLYP/6-311G*//M06-2X/6-31G* level (Table S1†). The *y* values of 1′ (0.50) and 6′ (0.46) are comparable to those of DFTh (0.50) and DFFu (0.45), respectively, which is in agreement with the experimental variable-temperature ^1^H NMR results.

The electrochemical properties of 1–6 were examined using cyclic voltammetry to gain insight into the electronic effects of the substituents (Fig. S6 and S7 and Table S2†). As is the case for DFTh and DFFu, 1–6 exhibit two reversible oxidation waves and two reversible reduction waves, thus indicating sufficient stability of these molecules toward redox reactions. The σ-inductive effects of the cyano residues of 1, 3, and 6 induce subtle but distinctive positive shifts of 0.06 V in the oxidation and reduction potentials relative to those of DFTh and DFFu. This indicates that the electrochemical properties of DFTh and DFFu can be tuned by functionalization of the outer six-membered rings. In the electronic absorption spectra (Fig. S8 and S9†), the absorption maxima of 1–6 are almost equal to the corresponding values for DFTh (609 nm) and DFFu (601 nm), and thus the optical gaps are retained after the introduction of substituents; however, substitution decreases the molar absorption coefficients in the long-wavelength region.

## Conclusions

We have developed site-selective radical reactions of kinetically stabilized open-shell singlet diradicaloids, *i.e.*, difluoreno[3,4-*b*:4′,3′-*d*]thiophene (DFTh) and difluoreno[3,4-*b*:4′,3′-*d*]furan (DFFu), with tributyltin hydride (HSn(*n*-Bu)_3_) and various azo-based radical initiators. The radical hydrogenation of DFTh/DFFu by HSn(*n*-Bu)_3_ occurs at the *ipso*-carbon of the five-membered rings, where the highest spin density is located. On the other hand, the radical coupling between DFTh/DFFu and the neutral radical derived from the decomposition of the azo-based radical initiators occurs at carbon atoms on the peripheral six-membered rings, where a relatively large spin density resides. This reactivity of the difluorenoheteroles demonstrates their open-shell π-radical nature,^11^ and is consistent with the singlet–triplet energy gaps of −4.3/−4.9 kcal mol^−1^, which indicate that DFTh/DFFu possess moderate diradical character. Theoretical calculations confirmed that the site-selectivity is controlled by the balance between the diradical activity of DFTh/DFFu and the steric hindrance. The one-pot reactions of DFTh/DFFu with azo-based radical initiators and HSn(*n*-Bu)_3_, followed by the dehydrogenation of the resulting products by DDQ, affords functionalized DFTh/DFFu derivatives. Although the substituents introduced in this way have marginal effects on the electronic properties, the present procedure provides a new approach to the late-stage functionalization of polycyclic conjugated diradicaloids. A strategy to access π-functionalized DFTh/DFFu derivatives is currently under investigation in our group.

## Data availability

The ESI† contains detailed description for the synthetic method and computational method. The supplementary spectroscopic and crystallographic data were also provided.

## Author contributions

N. Tabata synthesized the compounds and contributed to most of the experimental work. T. Uchino conducted additional complementary experiments. S.-i. Kato, Y. Shiota, and K. Yoshizawa performed the theoretical calculations. N. Tabata, C. Kitamura, and S.-i. Kato conducted the characterization of compounds. S.-i. Kato and Y. Shiota wrote the manuscript and played a critical role in the discussion of the experimental design, project direction, experiments and results, as well as the preparation of the manuscript. All authors discussed the results and commented on the manuscript.

## Conflicts of interest

There are no conflicts to declare.

## Supplementary Material

SC-014-D3SC00381G-s001

SC-014-D3SC00381G-s002

## References

[cit1] Abe M. (2013). Chem. Rev..

[cit2] Kubo T. (2015). Chem. Lett..

[cit3] Nakano M., Champagne B. (2015). J. Phys. Chem. Lett..

[cit4] Casado J. (2017). Top. Curr. Chem..

[cit5] Dong S., Li Z. (2022). J. Mater. Chem. C.

[cit6] Chen Z. X., Li Y., Huang F. (2021). Chem.

[cit7] Diradicaloids, ed. J. Wu, Jenny Stanford Publishing, New York, 2022

[cit8] Tobe Y. (2018). Top. Curr. Chem..

[cit9] Zeng Z., Shi X., Chi C., Navarrete J. T. L., Casado J., Wu J. (2015). Chem. Soc. Rev..

[cit10] Moles Quintero S., Haley M. M., Kertesz M., Casado J. (2022). Angew. Chem., Int. Ed..

[cit11] Stuyver T., Chen B., Zeng T., Geerlings P., De Proft F., Hoffmann R. (2019). Chem. Rev..

[cit12] Kolc J., Michl J. (1970). J. Am. Chem. Soc..

[cit13] Kolc J., Michl J. (1973). J. Am. Chem. Soc..

[cit14] Flynn C. R., Michl J. (1974). J. Am. Chem. Soc..

[cit15] Castellan A., Kolc J., Michl J. (1978). J. Am. Chem. Soc..

[cit16] Trahanovsky W. S., Lorimor S. P. (2006). J. Org. Chem..

[cit17] Uchida K., Ito S., Nakano M., Abe M., Kubo T. (2016). J. Am. Chem. Soc..

[cit18] Šolomek T., Ravat P., Mou Z., Kertesz M., Juríček M. (2018). J. Org. Chem..

[cit19] Dressler J. J., Barker J. E., Karas L. J., Hashimoto H. E., Kishi R., Zakharov L. N., MacMillan S. N., Gomez-Garcia C. J., Nakano M., Wu J. I., Haley M. M. (2020). J. Org. Chem..

[cit20] Guo J., Yang Y., Dou C., Wang Y. (2021). J. Am. Chem. Soc..

[cit21] Tian X., Guo J., Sun W., Yuan L., Dou C., Wang Y. (2022). Chem.–Eur. J..

[cit22] Kubo T., Sakamoto M., Akabane M., Fujiwara Y., Yamamoto K., Akita M., Inoue K., Takui T., Nakasuji K. (2004). Angew. Chem., Int. Ed..

[cit23] London G., von Wantoch Rekowski M., Dumele O., Schweizer W. B., Gisselbrecht J.-P., Boudon C., Diederich F. (2014). Chem. Sci..

[cit24] Miyoshi H., Nobusue S., Shimizu A., Hisaki I., Miyata M., Tobe Y. (2014). Chem. Sci..

[cit25] Hu P., Lee S., Herng T. S., Aratani N., Gonçalves T. P., Qi Q., Shi X., Yamada H., Huang K.-W., Ding J., Kim D., Wu J. (2016). J. Am. Chem. Soc..

[cit26] Hu P., Lee S., Park K. H., Das S., Herng T. S., Gonçalves T. P., Huang K. W., Ding J., Kim D., Wu J. (2016). J. Org. Chem..

[cit27] Qiu S., Wang C., Xie S., Huang X., Chen L., Zhao Y., Zeng Z. (2018). Chem. Commun..

[cit28] Majewski M. A., Chmielewski P. J., Chien A., Hong Y., Lis T., Witwicki M., Kim D., Zimmerman P. M., Stępień M. (2019). Chem. Sci..

[cit29] Hayashi H., Barker J. E., Cárdenas Valdivia A., Kishi R., MacMillan S. N., Gómez-García C. J., Miyauchi H., Nakamura Y., Nakano M., Kato S.-i., Haley M. M., Casado J. (2020). J. Am. Chem. Soc..

[cit30] Zhen C.-J., Lu S.-F., Lin M.-H., Wu J.-T., Chao I., Lin C.-H. (2021). Chem.–Eur. J..

[cit31] Barker J. E., Price T. W., Karas L. J., Kishi R., MacMillan S. N., Zakharov L. N., Gómez-García C. J., Wu J. I., Nakano M., Haley M. M. (2021). Angew. Chem., Int. Ed..

[cit32] Fu X., Zhao D. (2015). Org. Lett..

[cit33] Ravat P., Šolomek T., Rickhaus M., Häussinger D., Neuburger M., Baumgarten M., Juríček M. (2016). Angew. Chem., Int. Ed..

[cit34] Dressler J. J., Teraoka M., Espejo G. L., Kishi R., Takamuku S., Gómez-García C. J., Zakharov L. N., Nakano M., Casado J., Haley M. M. (2018). Nat. Chem..

[cit35] Ravat P., Šolomek T., Häussinger D., Blacque O., Juríček M. (2018). J. Am. Chem. Soc..

[cit36] Sahara K., Abe M., Zipse H., Kubo T. (2020). J. Am. Chem. Soc..

[cit37] Čavlović D., Häussinger D., Blacque O., Ravat P., Juríček M. (2022). JACS Au.

[cit38] Mori S., Akita M., Suzuki S., Asano M. S., Murata M., Akiyama T., Matsumoto T., Kitamura C., Kato S.-i. (2020). Chem. Commun..

[cit39] Mori S., Moles Quintero S., Tabaka N., Kishi R., González Núñez R., Harbuzaru A., Ponce Ortiz R., Marín-Beloqui J., Suzuki S., Kitamura C., Gómez-García C. J., Dai Y., Negri F., Nakano M., Kato S.-i., Casado J. (2022). Angew. Chem., Int. Ed..

[cit40] Kato S.-i. (2021). Adv. Phys. Org. Chem..

[cit41] Frisch M. J. , *et al.*, Gaussian 16, revision C.01, Gaussian, Inc., Wallingford, CT, 2016; for the full author list, see the ESI

[cit42] We have also tested the reaction of DFTh with benzoyl peroxide (BPO), 2,2,6,6-tetramethylpiperidine 1-oxyl (TEMPO), or (2,4,6-trimethylbenzoyl)diphenylphosphine oxide (TMDPO) (Scheme S3†). In the cases of BPO or TMDPO, only the decomposition of DFTh was observed, and unidentified polar materials were obtained; almost no reaction occurred between DFTh and TEMPO

[cit43] The activation energy for the reaction of Int2 with AIBN was calculated to be +41.0 kcal mol^−1^, and that for the reaction of Int2 with AIBNH, which is possibly formed by the H-atom abstraction of Int2 by AIBN, was calculated to be +23.2 kcal mol^−1^ (Fig. S4 and Scheme S4†). Both activation energy values were found to be higher than the activation energy (+8.2 kcal mol^−1^) required for the reaction of Int2 with CIPR

[cit44] According to DFT calculations at the B3LYP/6-31G** level, the dimerization of CIPR is an exothermic reaction (50.9 kcal mol^−1^) (Scheme S5†). This value is larger than that of the exothermic energy (39.4 kcal mol^−1^) required for the formation of Int3 from Int2, suggesting that the dimerization of CIPR competes with the H-atom abstraction of Int2 by CIPR

