# Supplementary material for: Site-selective radical reactions of kinetically stable open-shell singlet diradicaloid difluorenoheteroles with tributyltin hydride and azo-based radical initiators
Source: Chem Sci. 2023 May 2;14(22):5974–82. doi: 10.1039/d3sc00381g (PMC10246680; doi:10.1039/d3sc00381g)
Supplement: SC-014-D3SC00381G-s001 [file SC-014-D3SC00381G-s001.pdf]

Electronic Supplementary Material (ESI) for Chemical Science.
This journal is © The Royal Society of Chemistry 2023

Electronic supplementary information

Site-selective radical reactions of kinetically stable open-shell singlet diradicaloid difluorenoheteroles with tributyltin hydride and azo-based radical initiators

Naoki Tabata,[a] Takumi Uchino,[a] Chitoshi Kitamura,[a] Kazunari Yoshizawa,[b] Yoshihito Shiota,*[b] and Shin-ichiro Kato *[a]

[a] *Department of Materials Chemistry, School of Engineering, The University of Shiga Prefecture, 2500 Hassaka-cho, Hikone, Shiga 522-8533, Japan.*
kato.s@mat.usp.ac.jp

[b] *Institute for Materials Chemistry and Engineering (IMCE), Kyushu University, 744 Motooka, Nishi-ku, Fukuoka 819-0395, Japan.*
shiota@ms.ifoc.kyushu-u.ac.jp

Table of Contents

1. Experimental Details: Synthesis of Compounds (S2)
2. Supporting Scheme, Figures, and Tables (S9)
3. ^{1}H and ^{13}C NMR Spectral Data (S19)
4. Theoretical Calculations (S32)
5. References (S82)

1. Experimental Details: Synthesis of Compounds

Commercially available reagents and solvents were used as received. **DFTh** and **DFFu** were prepared according to the literature procedures.[1,2] An oil bath was used as the heat source. Column chromatography and plug filtrations were carried out with SiO_2. Thin-layer chromatography (TLC) was conducted on aluminum sheets coated with SiO_2 60 F_{254}. Melting points (M.p.) were measured with a capillary tube (Stanford Research Systems OPTiMelt MPA100). Recycling gel-permeation chromatography (JAIGEL LC-918 or LaboACE LC-5060) was performed with UV detectors using 1H and 2H polystyrene columns eluted with $CHCl_3$. ^1H and ^{13}C NMR spectra were recorded on a spectrometer (JEOL JNM-ECS400) at 400 MHz for ^1H and 100 MHz for ^{13}C. $CDCl_3$ or *p*-xylene-d_{10} were used as a solvent, and residual solvent signal in the ^1H and ^{13}C NMR spectra was used as an internal reference. To avoid the effect of DCl on the spectral broadening of diradicaloids, Et_3N was included in $CDCl_3$. HRMS (Thermo Fisher Scientific LTQ Orbitrap XL) spectrometric analyses were conducted in a positive mode. Electronic absorption (JASCO V-670 or JV-550) spectra were measured in a cuvette of 1 cm at room temperature. Cyclic voltammetry (EC Frontier ECstat-100) was performed using a cell equipped with a platinum wire working electrode, a platinum wire counter electrode, and an Ag/$AgNO_3$ reference electrode. All electrochemical measurements were performed in CH_2Cl_2 solution (ca. 5×10^{-4} mol L^{-1}) containing 0.1 mol L^{-1} [(*n*-Bu)$_4$N][PF_6] at room temperature. All potentials are referenced to the ferrocenium/ferrocene (Fc^+/Fc) couple, which was used as a standard.

Preparation of DFThH

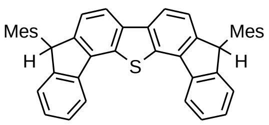

DFThH

To a dry toluene solution of **DFTh** (30 mg, 52 µmol) was added tributyltin hydride (0.14 mL, 0.50 mmol, 10 equiv) at room temperature under a nitrogen atmosphere. The resulting solution was stirred at 110 °C for 6 h. After the solvent was removed under reduced pressure, the residue was purified by column chromatography (silica gel, toluene/hexane = 1:3) to give **DFThH** (29 mg, 49 µmol) in 97% yield as a white solid. The ^1H NMR spectral data are in agreement with those reported previously.[2]

Preparation of DFFuH

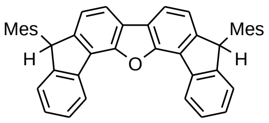

DFFuH

To a dry toluene solution of **DFFu** (30 mg, 52 µmol) was added tributyltin hydride (0.14 mL, 0.50 mmol, 10 equiv) at room temperature under a nitrogen atmosphere. The resulting solution was stirred at 110 °C for 6 h. After the solvent was removed under reduced pressure, the residue was purified by column chromatography (silica gel, toluene/hexane = 1:3) to give **DFFuH** (24 mg, 41 µmol) in 78% yield as a white solid. The ^1H NMR spectral data are in agreement with those reported previously.[1]

General procedure for the synthesis of 1H–6H. A mixture of **DFTh** or **DFFu** (1 equiv) and azo radical initiator (1.5–5 equiv) in was stirred dry toluene at 110 °C or in dry *p*-xylene at 120 °C for 3–20 min under a nitrogen atmosphere; the color of the mixture changed from blue to green. Tributyltin

hydride was added to the mixture; the mixture gradually became pale yellow. After the reaction solvent was removed under reduced pressure, the residue was dissolved with CH_2Cl_2. This solution was washed with brine, dried over anhydrous Na_2SO_4, and concentrated under reduced pressure. The residue was subjected to column chromatography (silica gel) to give **1H–6H**.

Preparation of 1H

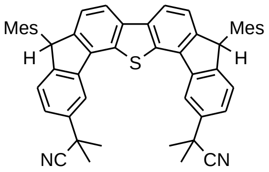

1H

In dry toluene (20 mL), **DFTh** (50 mg, 84 µmol) was allowed to react with 2,2′-azobis(isobutyronitrile) (AIBN; 69 mg, 0.42 mmol, 5 equiv) for 20 min and then tributyltin hydride (0.23 mL, 0.84 mmol, 10 equiv) for 1 h according to the general procedure for the synthesis of **1H–6H**. The crude product was purified by column chromatography (silica gel, CH_2Cl_2) to give **1H** (40 mg, 55 µmol) in 65% yield as a white solid. Mp 294–296 °C. ^1H NMR (400 MHz, $CDCl_3$, diastereomer mixture): δ 8.39 (2H, s), 8.17 (2H, d, J = 7.8 Hz), 7.49 (2H, d, J = 7.8 Hz), 7.40 (2H, d, J = 7.8 Hz), 7.35 (2H, d, J = 7.8 Hz), 7.07 (2H, s), 6.67 (2H, s), 5.70 (2H, s), 2.74 (6H, s), 2.30 (6H, s), 1.94 (12H, s), 1.10 (3H, s), 1.05 (3H, s) ppm. ^{13}C NMR (100 MHz, $CDCl_3$): δ 147.40, 146.99, 146.89, 141.19, 141.36, 140.81, 138.00, 137.97, 137.86, 136.79, 135.20, 134.78, 133.16, 132.27, 132.23, 130.74, 129.15, 124.93, 124.54, 124.44, 121.03, 120.90, 118.63, 50.33, 37.79, 29.71, 29.64, 22.02, 21.03, 18.92, 18.81 (31 signals out of 52 expected). UV–vis (CH_2Cl_2) λ_{max} (relative intensity): 265 (1.0), 285 (0.65), 296 (0.68), 308 (0.67), 346 (0.03) nm. HRMS (ESI, positive): m/z calcd for $C_{52}H_{46}N_2S$ 730.3376, found 730.3380 [M^+].

Preparation of 2H

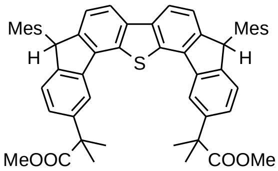

2H

In dry toluene (20 mL), **DFTh** (50 mg, 84 µmol) was allowed to react with dimethyl 2,2'-azobis(2-methylpropionate) (V-601™; 97 mg, 0.42 mmol, 5 equiv) for 20 min and then tributyltin hydride (0.23 mL, 0.84 mmol, 10 equiv) for 1 h according to the general procedure for the synthesis of **1H–6H**. The crude product was purified by column chromatography (silica gel, CH_2Cl_2/hexane = 9:1) to give **2H** (35 mg, 42 µmol) in 51% yield as a white solid. Mp 238–241 °C (decomp). ^1H NMR (400 MHz, $CDCl_3$, diastereomer mixture): δ 8.29 (2H, s), 8.14 (2H, d, J = 8.0 Hz), 7.39 (2H, d, J = 8.0 Hz), 7.34 (2H, d, J = 8.0 Hz), 7.28 (2H, dd, J = 8.0 & 2.8 Hz), 7.06 (2H, s), 6.66 (2H, s), 5.68 (2H, s), 3.75 (6H, s), 2.73 (6H, s), 2.30 (6H, s), 1.84 (6H, s), 1.82 (6H, s), 1.11 (3H, s), 1.07 (3H, s) ppm. ^{13}C NMR (100 MHz, $CDCl_3$): δ 177.51, 146.83, 146.73, 146.26, 144.20, 140.43, 138.08, 137.86, 136.61, 135.29, 135.26, 135.19, 133.53, 133.50, 132.15, 130.69, 129.09, 125.26, 124.05, 120.98, 120.52, 119.15, 52.52, 50.31, 46.83, 31.11, 26.92, 26.84, 26.75, 21.98, 21.03, 19.00, 18.89 (33 signals out of 52 expected) ppm. UV–vis (CH_2Cl_2) λ_{max} (relative intensity): 266 (1.0), 291 (0.96) nm. HRMS (ESI, positive): m/z calcd for $C_{54}H_{52}O_4S$ 796.3580, found 796.3576 [M^+].

Preparation of 3H

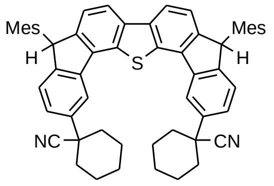

3H

In dry *p*-xylene (8 mL), **DFTh** (50 mg, 84 μmol) was allowed to react with 1,1'-azobis(cyclohexane-1-carbonitrile) (V-40™; 62 mg, 0.25 mmol, 3.0 equiv) for 10 min and then tributyltin hydride (0.23 mL, 0.84 mmol, 10 equiv) for 1 h according to the general procedure for the synthesis of **1H**–**6H**. The crude product was purified by column chromatography (silica gel, toluene) to give **3H** (60 mg, 74 μmol) in 94% yield as a white solid. Mp 258–261 °C (decomp). ^{1}H NMR (400 MHz, $CDCl_3$, diastereomer mixture): δ 8.39 (2H, s), 8.16 (2H, dd, J = 8.0 Hz & 1.3 Hz), 7.53 (2H, td, J = 8.0 Hz & 1.7 Hz), 7.40 (2H, d, J = 8.0 Hz), 7.35 (2H, dd, J = 8.0 Hz & 3.4 Hz), 7.07 (2H, s), 6.67 (2H, s), 5.70 (2H, s), 2.74 (6H, s), 2.35–2.38 (4H, m), 2.30 (6H, s), 2.04–1.94 (16H, m), 1.09 (3H, s), 1.05 (3H, s) ppm. ^{13}C NMR (100 MHz, $CDCl_3$): δ 147.46, 147.42, 147.03, 146.90, 141.23, 140.82, 138.01, 137.88, 136.77, 135.25, 134.92, 133.24, 132.24, 132.21, 130.75, 129.15, 125.13, 124.50, 123.11, 121.04, 120.84, 119.13, 50.33, 44.90, 37.90, 37.82, 25.17, 23.98, 22.00, 21.04, 18.91, 18.82 (32 signals out of 54 expected) ppm. UV–vis (CH_2Cl_2) λ_{max} (relative intensity): 265 (1.0), 295 (0.64), 308 (0.64) nm. HRMS (ESI, positive): *m/z* calcd for $C_{58}H_{54}N_2S$ 810.4002, found 810.4013 [M^+].

Preparation of 4H

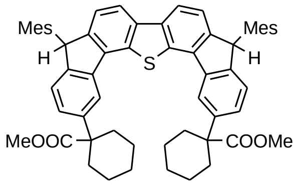

4H

In dry *p*-xylene (10 mL), **DFTh** (50 mg, 84 μmol) was allowed to react with dimethyl 1,1'-azobis(1-cyclohexanecarboxylate) (VE-073™; 78 mg, 0.25 mmol, 3.0 equiv) for 10 min and then tributyltin hydride (0.23 mL, 0.84 mmol, 10 equiv) for 1 h according to the general procedure for the synthesis of **1H**–**6H**. The crude product was purified by column chromatography (silica gel, toluene) to give **4H** (50 mg, 58 μmol) in 68% yield as a white solid. Mp 214–217 °C (decomp). ^{1}H NMR (400 MHz, $CDCl_3$, diastereomer mixture): δ 8.37 (2H, s), 8.13 (2H, dd, J = 8.0 Hz & 1.6 Hz), 7.40 (2H, d, J = 9.0 Hz), 7.38 (2H, d, J = 8.0 Hz), 7.27 (2H, d, J = 8.0 Hz), 7.06 (2H, s), 6.66 (1H, s), 6.65 (1H, s), 5.68 (1H, s), 5.67 (1H, s), 3.74 (6H, s), 2.80–2.73 (4H, m), 2.73 (6H, s), 2.29 (6H, s), 2.03–2.00 (4H, m), 1.93–1.80 (4H, m), 1.78–1.70 (2H, m), 1.64–1.61 (4H, m), 1.43–1.40 (2H, m), 1.10 (3H, s), 1.05 (3H, s) ppm; ^{13}C NMR (100 MHz, $CDCl_3$): δ 175.94, 146.79, 146.68, 146.36, 146.31, 143.36, 140.49, 138.10, 137.85, 136.59, 135.38, 135.20, 133.59, 132.17, 130.68, 129.08, 125.35, 124.08, 120.96, 120.47, 119.72, 52.31, 51.17, 50.27, 35.26, 35.16, 35.11, 25.87, 24.06, 21.98, 21.03, 18.98, 18.87 (33 signals out of 56 expected) ppm. UV–vis (CH_2Cl_2) λ_{max} (relative intensity): 266 (1.0), 308 (0.57), 392 (0.17) nm. HRMS (ESI, positive): *m/z* calcd for $C_{60}H_{60}O_4S$ 876.4206, found 876.4216 [M^+].

Preparation of 5H

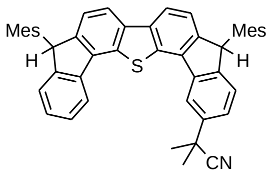

5H

In dry toluene (20 mL), **DFTh** (50 mg, 84 μmol) was allowed to react with 2,2′-azobis(isobutyronitrile) (AIBN; 21 mg, 0.13 mmol, 1.5 equiv) for 3 min and then tributyltin hydride (0.12 mL, 0.42 mmol, 5.0 equiv) for 1 h according to the general procedure for the synthesis of **1H**–**6H**. The crude product was purified by column chromatography (silica gel, toluene/hexane = 1:3 → pure toluene) to give **5H** (26 mg, 42 μmol) in 47% yield as a white solid. Mp 212–215 °C. ^{1}H NMR (400 MHz, $CDCl_3$): δ 8.30 (1H, s), 8.26 (1H, d, J = 7.6 Hz), 8.16 (1H, dd, J = 7.6 & 1.4 Hz), 8.15 (1H, dd, J = 7.6 & 2.0 Hz), 7.65 (1H, t, J = 7.6 Hz), 7.51 (1H, d, J = 7.4 Hz), 7.41–7.33 (5H, m), 7.07 (2H, s), 6.67 (2H, s), 5.71 (1H, s), 5.69 (1H, s), 2.75 (3H, s), 2.74 (3H, s), 2.30 (6H, s), 1.99 (3H, s), 1.98 (3H, s), 1.10 (3H, 2s), 1.08 (3H, s) ppm. ^{13}C NMR (100 MHz, $CDCl_3$): δ 148.23, 147.85, 147.37, 147.30, 146.96, 141.37, 141.26, 140.46, 138.42, 138.33, 138.21, 137.11, 136.94, 135.70, 135.45, 135.01, 133.98, 133.52, 132.73, 132.50, 131.07, 131.03, 129.47, 129.39, 129.21, 127.80, 127.74, 127.62, 125.31, 124.84, 124.77, 124.54, 122.69, 121.36, 121.24, 120.80, 118.91, 50.87, 50.81, 37.86, 30.10, 30.04, 22.32, 21.36, 19.31, 19.23, 19.15, 19.08 (48 signals out of 48 expected). UV–vis (CH_2Cl_2) λ_{max} (relative intensity): 266 (1.0), 294 (0.84), 306 (0.80), 346 (0.04) nm. HRMS (ESI, positive): *m*/*z* calcd for $C_{48}H_{41}NS$ 663.2954, found 663.2948 [M^+].

Preparation of 6H

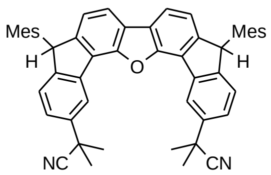

6H

In dry toluene (20 mL), **DFFu** (60 mg, 104 μmol) was allowed to react with 2,2′-azobis(isobutyronitrile) (AIBN; 51 mg, 0.31 mmol, 3 equiv) for 20 min and then tributyltin hydride (0.28 mL, 1.04 mmol, 10 equiv) for 1 h according to the general procedure for the synthesis of **1H**–**6H**. The crude product was purified by column chromatography (silica gel, CH_2Cl_2) to give **1H** (55 mg, 78 μmol) in 75% yield as a white solid. M.p. 250–252 °C; ^{1}H NMR (400 MHz, $CDCl_3$, diastereomer mixture): δ 8.55 (2H, d, J = 1.9 Hz), 7.90 (2H, dd, J = 7.9 & 1.9 Hz), 7.52 (2H, d, J = 7.9 & 1.9 Hz), 7.32 (2H, d, J = 7.9 Hz), 7.26 (2H, d, J = 6.2 Hz), 7.07 (2H, s), 6.68 (2H, s), 5.70 (2H, s), 2.74 (6H, s), 2.30 (6H, s), 2.01 (6H, s), 1.99 (6H, d, J = 1.9 Hz), 1.12 (3H, s), 1.10 (3H, s) ppm; ^{13}C NMR (100 MHz, $CDCl_3$): δ 151.50, 147.96, 147.22, 140.79, 139.61, 137.99, 137.85, 136.79, 133.48, 130.77, 129.15, 125.20, 124.87, 124.38, 124.02, 120.02, 119.65, 119.32, 50.40, 37.30, 31.75, 29.73, 29.67, 29.63, 29.57, 22.81, 21.98, 21.78, 21.04, 18.97, 18.96, 18.92, 18.91, 14.29 (34 signals out of 52 expected); UV–vis (CH_2Cl_2) λ_{max} (relative intensity): 296 (0.99), 303 (1.0), 321 (0.64) nm; HRMS (ESI, positive): *m*/*z* calcd for $C_{52}H_{46}N_2O$ 715.3682, found 715.3681 [M^+].

General procedure for the synthesis of 1–6. To a dry toluene solution of dihydro compounds **1H**–**6H** (1 equiv) was added DDQ (1.5–2 equiv), and the resulting mixture was stirred at 80 °C for 12 h under a nitrogen atmosphere; the color of the mixture became deep blue. The mixture was diluted with toluene and passed through a bed of silica gel. The filtrate was concentrated under removed pressure

to give **1**–**6**. An analytical sample was obtained by recycling gel-permeation chromatography.

Preparation of 1

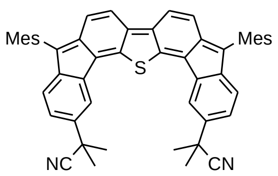

1

Dihydro compound **1H** (30 mg, 41 μmol) was allowed to react with DDQ (19 mg, 82 μmol, 2 equiv) in dry toluene (10 mL) according to the general procedure for the synthesis of **1**–**6** to give **1** (30 mg, 42 μmol) in almost quantitatively as a deep blue solid. Mp 197–199 °C (decomp). ^{1}H NMR (400 MHz, $CDCl_3$): δ 7.88 (2H, d, J = 1.5 Hz), 7.24 (2H, dd, J = 1.5 & 9.1 Hz), 7.20 (2H, d, J = 9.1 Hz), 7.01 (4H, s), 6.94 (2H, d, J = 7.9 Hz), 6.80 (2H, d, J = 9.1 Hz), 2.38 (6H, s), 2.14 (12H, s), 1.87 (12H, s) ppm. ^{13}C NMR (100 MHz, $CDCl_3$): δ 143.01, 141.84, 140.36, 138.10, 137.98, 137.27, 136.46, 135.70, 132.94, 130.11, 128.50, 124.78, 123.11, 119.84, 118.98, 37.72, 29.86, 29.32, 21.35, 20.58 (20 signals out of 22 expected) ppm. UV–vis (CH_2Cl_2) λ_{max}^{abs} (ε): 280 (58200), 613 (69100), 835 (3900) nm. HRMS (ESI, positive): m/z calcd for $C_{52}H_{44}N_2S$ 726.3219, found 728.3216 [M^+].

Preparation of 2

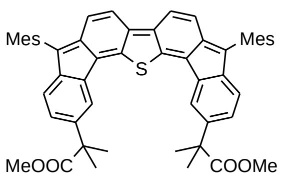

2

Dihydro compound **2H** (25 mg, 31 μmol) was allowed to react with DDQ (14 mg, 63 μmol, 2 equiv) in dry toluene (10 mL) according to the general procedure for the synthesis of **1**–**6** to give **2** (18 mg, 23 μmol) in 73% yield as a deep blue solid. Mp 247–250 °C (decomp). ^{1}H NMR (400 MHz, $CDCl_3$): δ 7.72 (2H, s), 7.16 (2H, d, J = 9.1 Hz), 7.08 (2H, d, J = 6.6 Hz), 7.00 (4H, s), 6.88 (2H, d, J = 8.0 Hz), 6.76 (2H, d, J = 9.1 Hz), 3.78 (6H, s), 2.37 (6H, s), 2.13 (12H, s), 1.72 (12H, s) ppm. ^{13}C NMR (100 MHz, CD_2Cl_2): δ 177.22, 144.38, 143.21, 141.16, 138.14, 137.87, 137.38, 136.37, 135.80, 133.39, 130.43, 129.34, 128.63, 125.60, 124.80, 122.91, 120.90, 118.95, 52.60, 47.21, 26.74, 21.28, 20.48 (23 signals out of 23 expected) ppm. UV–vis (CH_2Cl_2) λ_{max}^{abs} (ε): 280 (48000), 613 (68000), 852 (3500) nm. HRMS (ESI, positive): m/z calcd for $C_{54}H_{50}O_4S$ 794.3424, found 794.3420 [M^+].

Preparation of 3

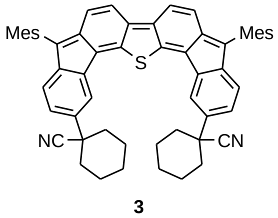

3

Dihydro compound **3H** (30 mg, 37 μmol) was allowed to react with DDQ (13 mg, 56 μmol, 1.5 equiv) in dry toluene (10 mL) according to the general procedure for the synthesis of **1**–**6** to give **3** (31 mg, 38 μmol) in almost quantitatively as a deep blue solid. Mp 192 °C (decomp). ^{1}H NMR (400 MHz, $CDCl_3$): δ 7.88 (2H, s), 7.28 (2H, d, J = 8.4 Hz), 7.19 (2H, d, J = 9.1 Hz), 7.01 (4H, s), 6.95 (2H, d), 6.79 (2H, d, J = 8.8 Hz), 2.38 (6H, s), 2.29 (4H, d, J = 6.5 Hz), 2.14 (12H, s), 1.98–1.92 (14H, m), 1.46–1.38 (2H, m) ppm. ^{13}C NMR (100 MHz, $CDCl_3$): δ 142.97, 141.86, 140.43, 138.09, 137.94, 137.28, 136.43, 135.66, 133.00, 130.15, 128.48, 125.42, 124.75, 123.08, 122.79, 120.27, 118.92, 44.88, 37.56, 29.86, 25.14, 23.86, 21.35, 20.60 (24 signals out of 24 expected) ppm.

UV–vis (CH_2Cl_2) λ_{max}^{abs} (ε): 280 (48300), 614 (45400), 864 (2100) nm. HRMS (ESI, positive): *m*/*z* calcd for $C_{58}H_{52}N_2S$ 808.3845, found 808.3855 [M^+].

Preparation of 4

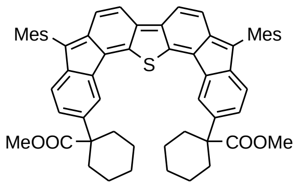

4

Dihydro compound **4H** (30 mg, 34 μmol) was allowed to react with DDQ (16 mg, 68 μmol, 2 equiv) in dry toluene (10 mL) according to the general procedure for the synthesis of **1**–**6** to give **4** (28 mg, 32 μmol) in 93% yield as a deep blue solid. Mp 237–240 °C (decomp). ^{1}H NMR (400 MHz, $CDCl_3$): δ 7.82 (2H, s), 7.16 (2H, d, *J* = 9.0 Hz), 7.14 (2H, d, 7.8 Hz), 7.00 (4H, s), 6.88 (2H, d, *J* = 7.8 Hz), 6.76 (2H, d, *J* = 9.0 Hz), 3.78 (6H, s), 2.68–2.65 (4H, m), 2.37 (6H, s), 2.13 (12H, s), 1.97–1.82 (8H, m), 1.76–1.68 (4H, m), 1.38–1.36 (4H, m) ppm. ^{13}C NMR (100 MHz, $CDCl_3$): δ 175.65, 143.14, 142.84, 141.01, 137.90, 137.74, 137.67, 137.32, 136.27, 135.50, 133.27, 130.44, 128.40, 125.44, 124.55, 122.84, 120.89, 118.61, 51.48, 35.07, 29.85, 25.76, 24.05, 21.34, 20.62 (25 signals out of 25 expected) ppm. UV–vis (CH_2Cl_2) λ_{max}^{abs} (ε): 280 (36400), 616 (36800), 868 (1900) nm. HRMS (ESI, positive): *m*/*z* calcd for $C_{60}H_{58}O_4S$ 874.4050, found 874.4064 [M^+].

Preparation of 5

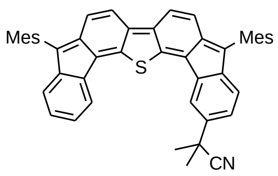

5

Dihydro compound **5H** (30 mg, 45 μmol) was allowed to react with DDQ (21 mg, 91 μmol, 2 equiv) in dry toluene (10 mL) according to the general procedure for the synthesis of **1**–**6** to give **5** (29 mg, 43 μmol) in 97% yield as a deep blue solid. Mp 198–200 °C (decomp). ^{1}H NMR (400 MHz, $CDCl_3$): δ 7.81 (1H, s), 7.77 (1H, d, *J* = 7.4 Hz), 7.30–7.26 (2H, m), 7.20 (1H, d, *J* = 7.4 Hz), 7.18 (1H, d, *J* = 7.4 Hz), 7.13 (1H, t, *J* = 7.4 Hz), 7.01 (4H, s), 6.94 (1H, d, *J* = 6.8 Hz), 6.92 (1H, d, *J* = 6.8 Hz), 6.78 (2H, d, *J* = 8.9 Hz), 2.38 (6H, s), 2.14 (6H, s), 2.12 (6H, s), 1.90 (6H, s) ppm. ^{13}C NMR (100 MHz, $CDCl_3$): δ 143.93, 142.44, 142.10, 141.92, 139.88, 138.43, 138.28, 138.00, 137.90, 137.83, 137.65, 137.30, 136.49, 135.97, 135.84, 135.34, 133.67, 132.54, 130.30, 130.16, 129.18, 128.46, 128.43, 128.37, 127.87, 127.26, 125.44, 124.84 124.76, 124.56, 123.48, 122.99, 122.85, 119.87, 118.94, 118.73, 37.50, 29.85, 29.45, 21.34, 20.56 (41 signals out of 48 expected) ppm. UV–vis (CH_2Cl_2) λ_{max} (ε): 279 (55000), 610 (80700), 845 (3800) nm. HRMS (ESI, positive): *m*/*z* calcd for $C_{48}H_{39}NS$ 661.2797, found 661.2801 [M^+].

Preparation of 6

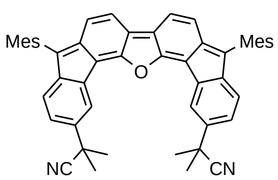

6

Dihydro compound **6H** (21 mg, 29.4 μmol) was allowed to react with DDQ (10 mg, 45.0 μmol, 1.5 equiv) in dry toluene (10 mL) according to the general procedure for the synthesis of **1**–**6** to give **6** (21 mg, 29 μmol,) in 97% yield as a deep blue solid. M.p. 266–268 °C (decomp.); ^{1}H NMR (400 MHz, $CDCl_3$): δ 8.08 (2H, d, *J* = 1.8 Hz), 7.28 (2H, dd, *J* = 1.8 & 8.0 Hz), 7.01 (4H, s), 6.97 (2H, d, *J* = 8.9 Hz), 6.95(2H, d, *J* = 8.9 Hz), 6.76 (2H, d, *J* = 8.8 Hz), 2.38 (6H, s), 2.12 (12H, s), 1.89

(12H, s) ppm; ^{13}C NMR (100 MHz, $CDCl_3$): δ 155.03, 143.69, 141.48, 140.25, 137.92, 137.30, 134.79, 130.29, 129.11, 128.46, 125.29, 124.92, 124.47, 122.91, 120.91, 118.41, 116.89, 37.37, 29.86, 29.26, 21.34, 20.51 (22 signals out of 22 expected) ppm; UV–vis (CH_2Cl_2) λ_{max}^{abs} (ε): 271 (59100), 602 (47500), 827 (3100) nm; HRMS (ESI, positive): *m*/*z* calcd for $C_{52}H_{44}N_2O$ 712.3448, found 712.3447 [M^+].

2. Supporting Scheme, Figures, and Tables

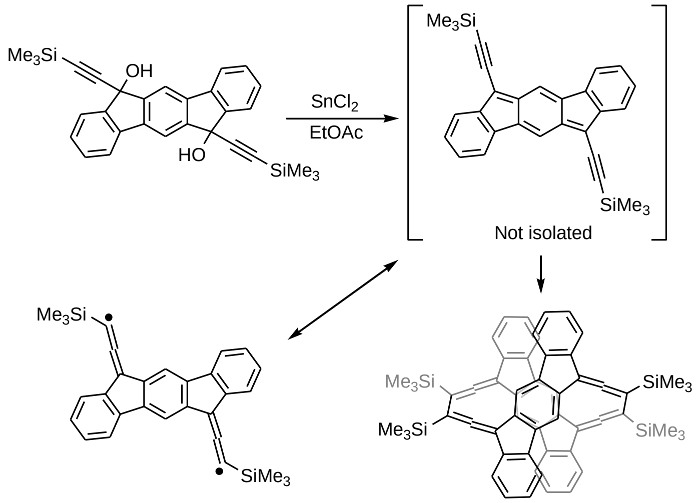

Scheme S1. Cyclo-dimerization of *in-situ* formed trimethylsilylethynyl-substituted indeno[1,2-*b*]fluorene derivative reported by Zhao and co-workers.[3]

Competitive reaction experiment: A mixture of **DFTh** (10.2 mg, 17.3 μmol) and **DFFu** (10.0 mg, 17.3 μmol) in toluene-d_8 (5 mL) was heated at 120 °C under a nitrogen atmosphere. Tributyltin hydride (0.10 mL, 0.35 mmol, 20 equiv) was added, and the resulting mixture was stirred for 0.5 h. The solution was directly transferred to the NMR tube, and the ^{1}H NMR spectrum was measured (Figure S1). The radio of **DFThH** to **DFFuH** to **DFFu** was determined to be 1.0 to 0.81 to 0.19; **DFTh** was almost completely consumed.

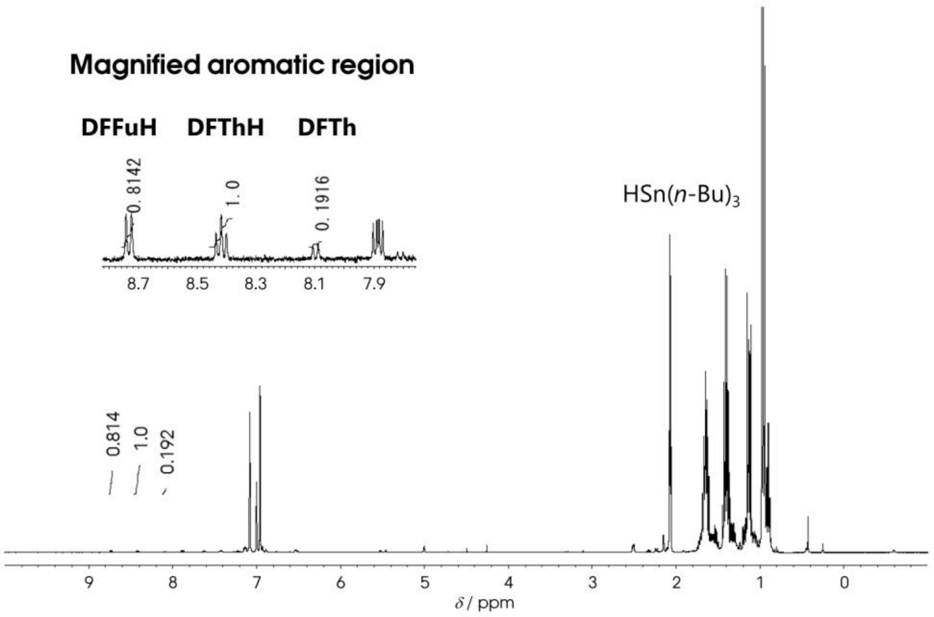

Figure S1. ^{1}H NMR spectrum of the products in toluene-d_8, which was obtained by the competitive reaction experiment using **DFTh**, **DFFu**, and tributyltin hydride.

Reaction of DFTh with AIBN: To a solution of **DFTh** (40 mg, 67 μmol) in dry toluene (10 mL) was added AIBN (33 mg, 202 μmol, 3.0 equiv) at room temperature. The resulting mixture was heated at 110 °C for *ca.* 10 min under a nitrogen atmosphere; the color of the solution rapidly changed from deep blue to pale blue. After the reaction solvent was removed under reduced pressure, the residue was subjected to recycling gel-permeation chromatography to give the mixture of **1** and **1H** (0.36:1, 21 mg) as a blue solid in a total yield of 42%; the ratio of **1** and **1H** was determined by NMR spectroscopy (Figure S2).

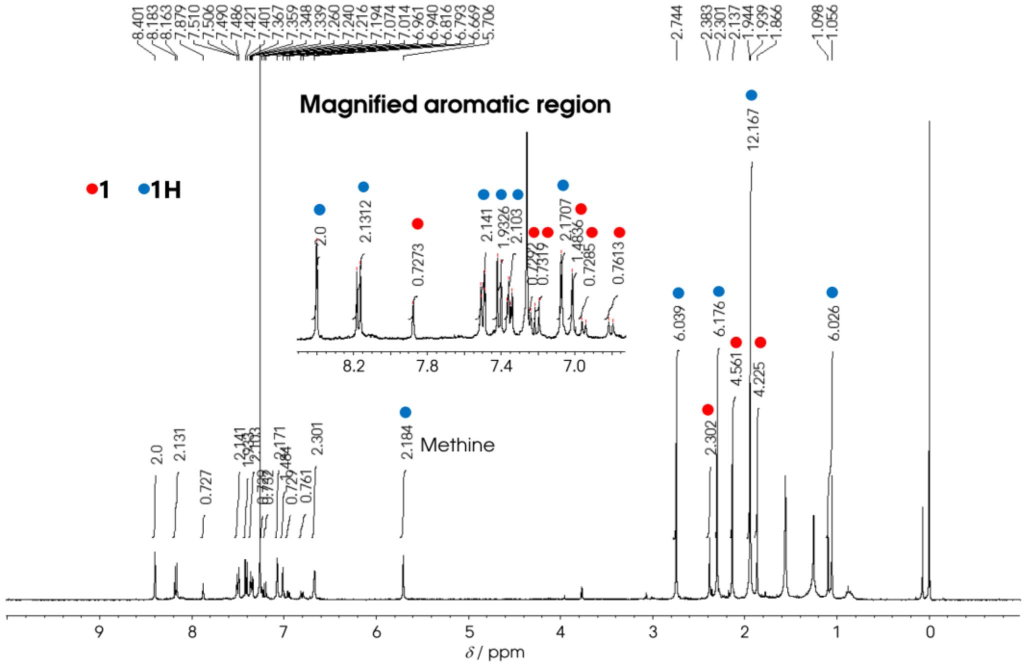

Figure S2. ^{1}H NMR spectrum of the mixture of **1** and **1H** in $CDCl_3$, which was obtained by the reaction of **DFTh** with AIBN in toluene at 110 °C for ca. 10 min.

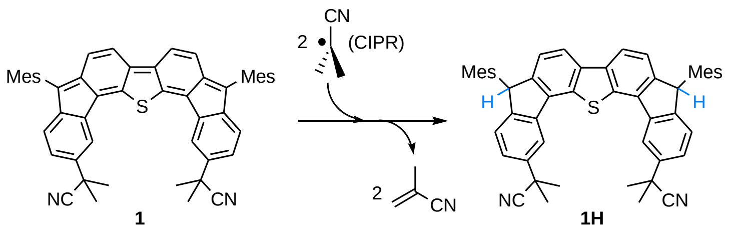

Scheme S2. Plausible reaction of **1** with CIPR to afford **1H**.

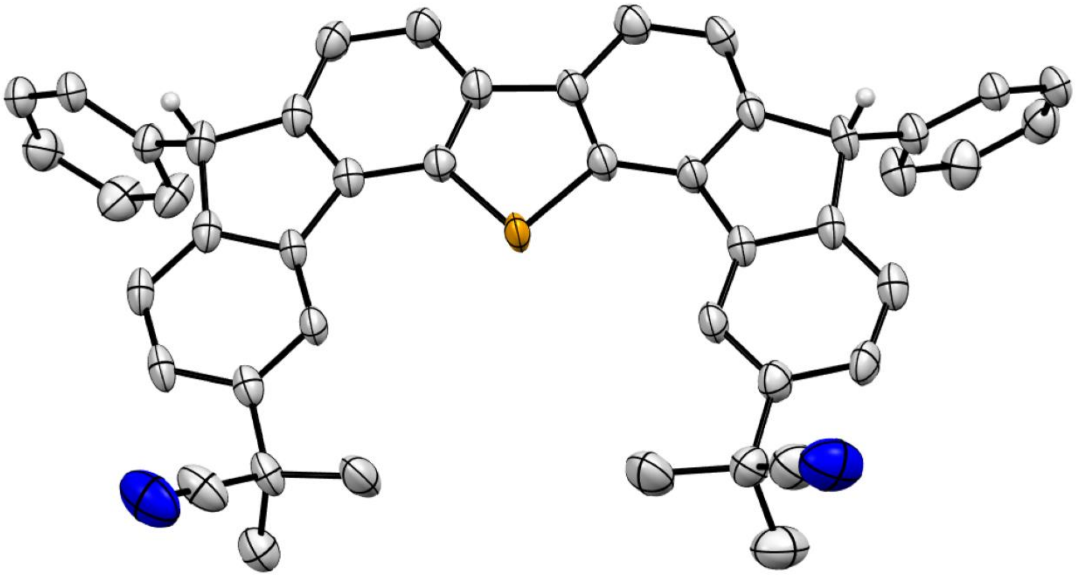

Figure S3. X-ray crystal structure of **1H** (meso compound). Displacement ellipsoids are shown at the 50% probability level. Most hydrogen atoms, Me groups, and the solvent molecule are omitted for clarity.

Low-temperature X-ray diffraction data for **1H** were collected on a Rigaku AFC10 diffractometer coupled to a Rigaku AFC HyPix-6000 detector with Mo Kα radiation (λ = 0.71073 Å) from an FR-E+ X-ray source. The diffraction images were processed and spaced using the CrysAlisPro software.[4] Using Olex2,[5] the structures were solved through intrinsic phasing using SHELXT[6] and refined against F^2 on all data by full-matrix least squares with SHELXL[7] following established refinement strategies. All non-hydrogen atoms were refined anisotropically. All hydrogen atoms bound to carbon were included in the model at geometrically calculated positions and refined using a riding model. The isotropic displacement parameters of all hydrogen atoms were fixed to 1.2 times the Ueq value of the atoms they are linked to (1.5 times for methyl groups).

1H: crystal data at 113 K for $C_{52}H_{46}N_2S{\cdot}CH_3CN$, M_r = 772.02, Triclinic, space group *P*-1, D_{calcd} = 1.076 g cm^{-3}, Z = 2, a = 11.0183(8) Å, b = 14.4087(10) Å, c = 17.3579(9) Å, α = 65.994(6)°, β = 102.478(7)°, γ = 65.994(6)°, V = 2383.1(3) Å^3; Mo-*K*α radiation, λ = 0.71073, μ = 0.104 mm^{-1}. Numbers of measured and unique reflections were 39193 [R_{int} = 0.1182, R_{sigma} = 0.1385] and 10929, respectively. Final $R(F)$ = 0.0884 for 545 parameters and 10929 reflections with $I > 2\sigma(I)$ (for all data, $R(F)$ and $wR(F^2)$ values are 0.1642 and 0.2488, respectively). CCDC-2231637.

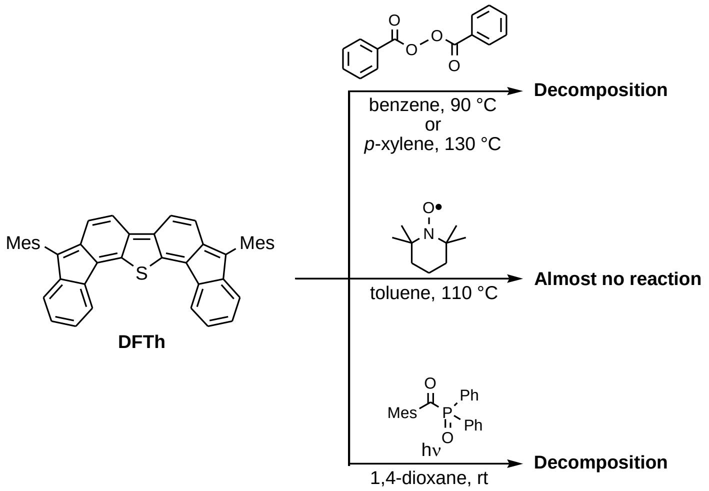

Scheme S3. Reactions of **DFTh** with benzoyl peroxide, 2,2,6,6-tetramethylpiperidine 1-oxyl, or (2,4,6-trimethylbenzoyl)diphenylphosphine oxide.

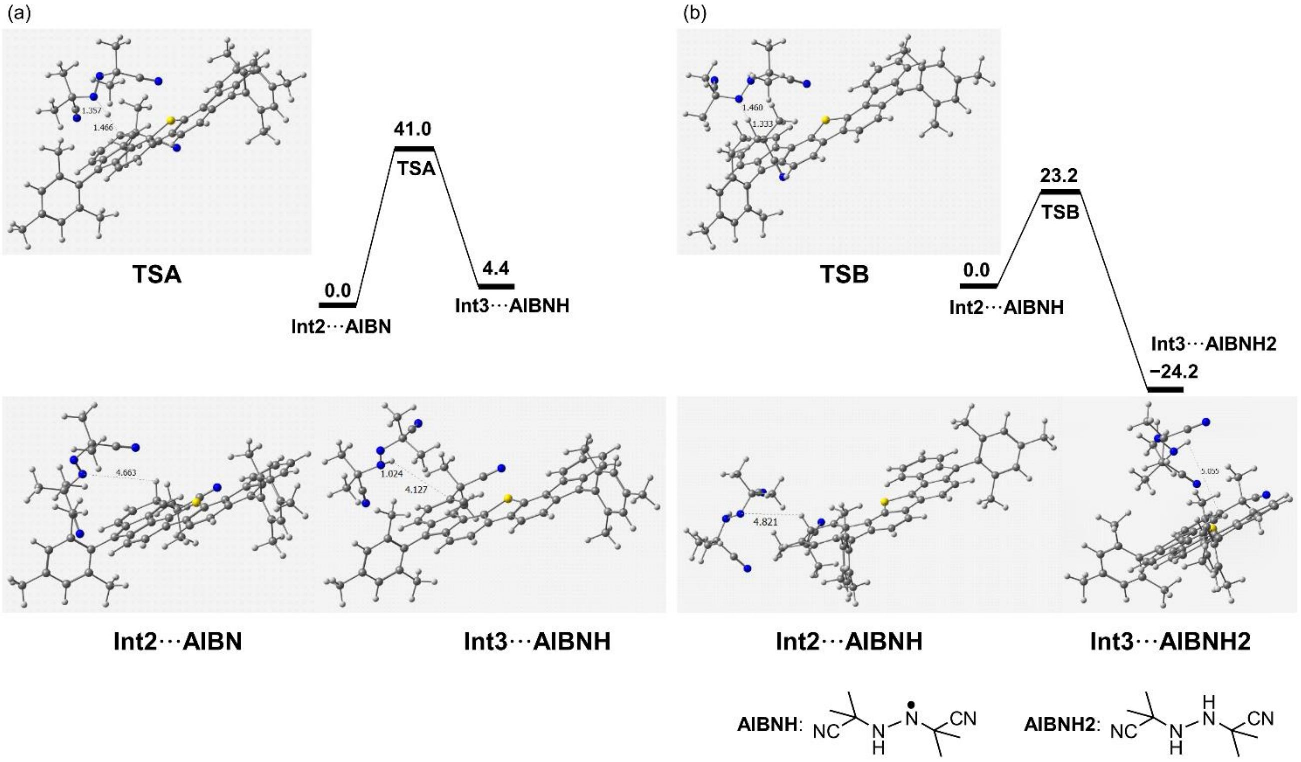

Figure S4. Potential energy diagrams for the H-atom transfer from **Int2** to (a) AIBN or (b) AIBNH to afford **Int3**, calculated at the B3LYP/6-31G** level; units are given in kcal/mol.

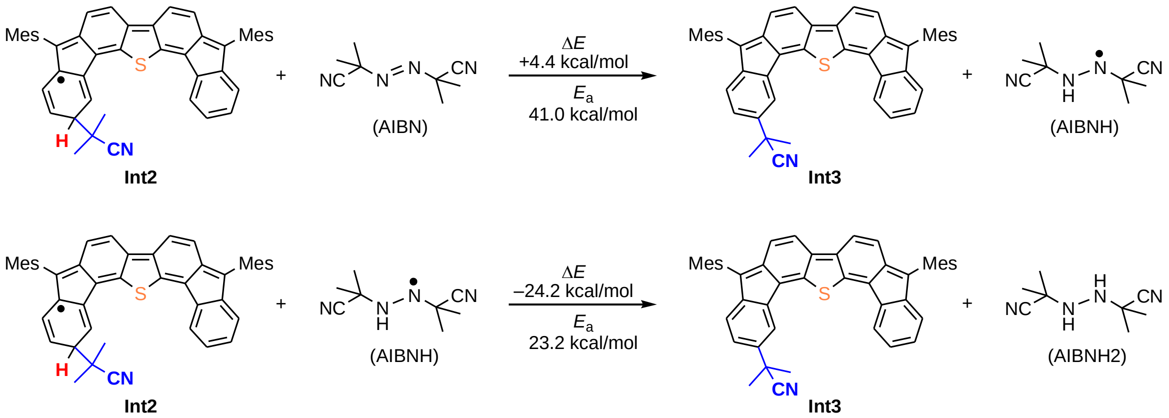

Scheme S4. H-atom transfer from **Int2** to AIBN or AIBNH to afford **Int3**, calculated at the B3LYP/6-31G** level.

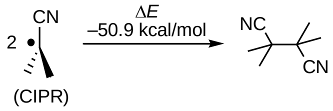

Scheme S5. Dimerizaition of CIPR calculated at the B3LYP/6-31G** level.

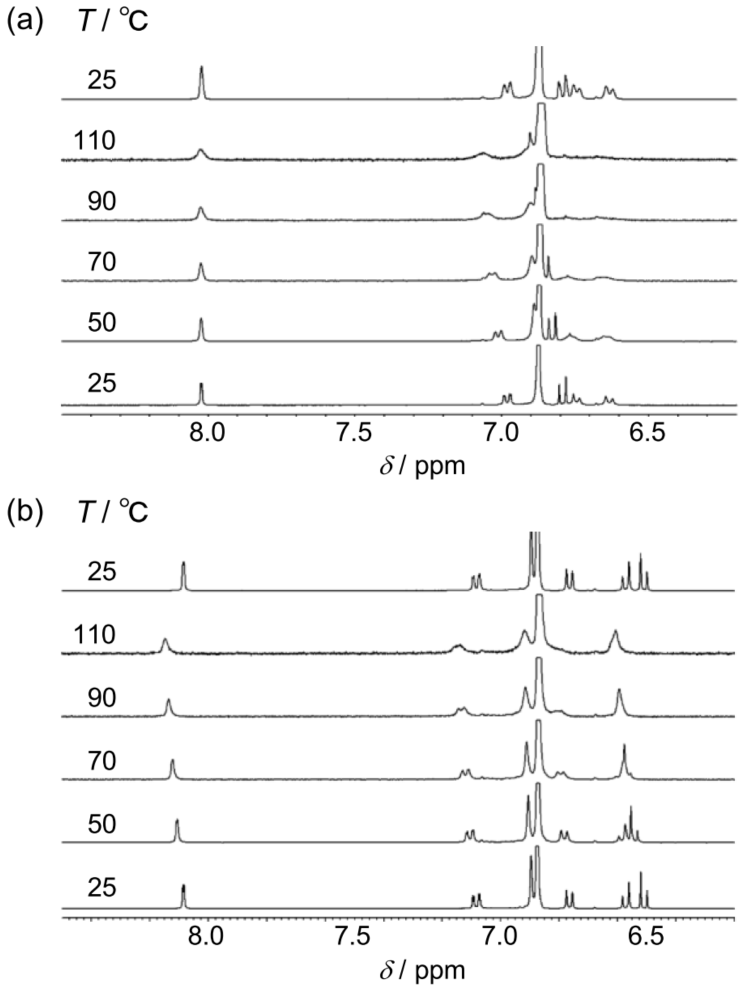

Figure S5. Temperature-dependent ^{1}H NMR spectra of (a) **1** and (b) **6** (*p*-xylene-d_{10}, 400 MHz). The original spectra were recovered upon cooling to 25 °C.

The open-shell singlet diradical character index y was calculated based on the natural orbital occupation number (NOON) of the LUMO in spin-unrestricted LC-BLYP calculation using 6-311G* basis set. According to the Yamaguchi scheme,[8] y value is expressed as

$$y = 1 - (2T / (1 + T^2))$$

where T is the orbital overlap between the corresponding orbital pairs and it can be presented using the NOON of HOMO and LUMO.

$$T = (n_{\mathrm{HOMO}} - n_{\mathrm{LUMO}}) / 2$$

The y values obtained by this method are listed in Table S1.

Table S1. Relative Energies and Open-Shell Singlet Diradical Character Indexes

Compd	ΔE (OS – CS)[a]	ΔE ($S_0 - T_1$)[b]	y [c]
**DFTh′**	–2.2	–3.7	0.50
**DFFu′**	–1.5	–4.3	0.45
**1′**	–2.3	–3.6	0.50
**2′**	–1.6	–4.2	0.46

[a] Open-shell singlet energy minus closed-shell singlet energy (kcal/mol^{-1}; (U)M06-2X/6-311G*).
[b] Open-shell singlet energy minus unrestricted triplet energy (kcal/mol^{-1}; (U)M06-2X/6-311G*).
[c] Open-shell singlet diradical character index.

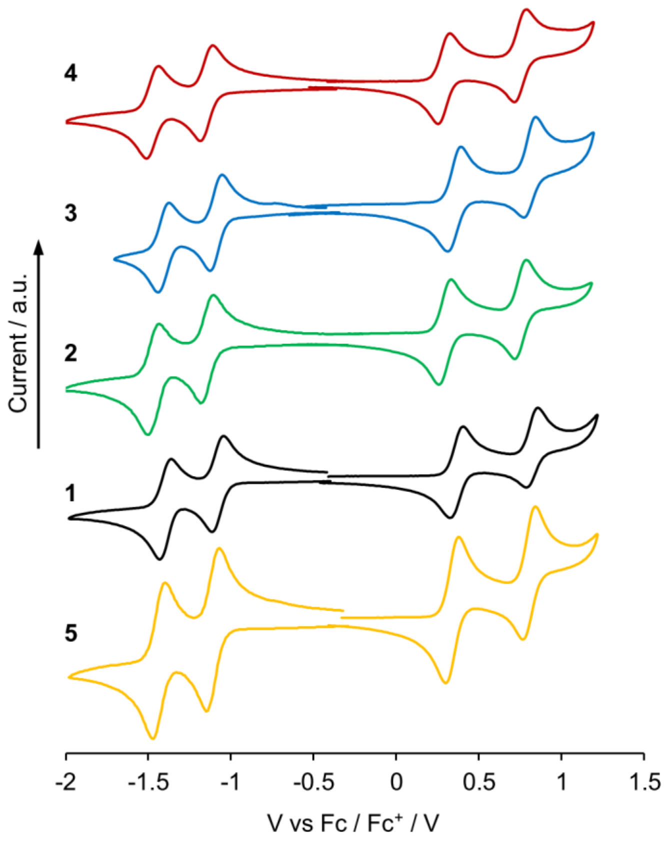

Figure S6. Cyclic voltammograms of **1**–**5** in CH_2Cl_2 containing 0.1 mol L^{-1} [(*n*-Bu)$_4$N][PF_6] as a supporting electrolyte at a scan rate of 100 mV s^{-1}.

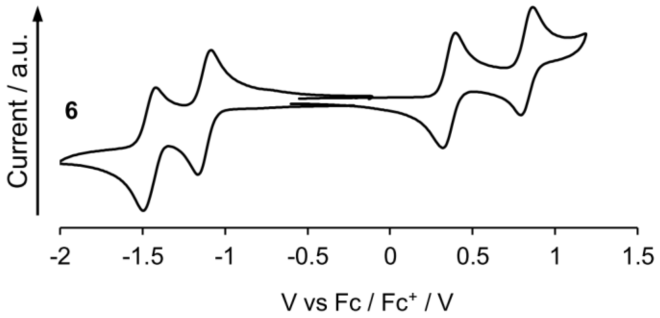

Figure S7. Cyclic voltammograms of **6** in CH_2Cl_2 containing 0.1 mol L^{-1} [(*n*-Bu)$_4$N][PF_6] as a supporting electrolyte at a scan rate of 100 mV s^{-1}.

Table S2. Redox Potentials Determined by Cyclic Voltammetry[a]

Cmpd.	E_{ox} [V]	E_{red} [V]	ΔE_{redox} [V] [b]
**DFTh**	+0.31	–1.14	1.45
	+0.78	–1.47	
**1**	+0.38	–1.08	1.46
	+0.83	–1.40	
**2**	+0.30	–1.14	1.44
	+0.75	–1.47	
**3**	+0.36	–1.09	1.45
	+0.81	–1.41	
**4**	+0.29	–1.15	1.44
	+0.75	–1.48	
**5**	+0.34	–1.11	1.45
	+0.81	–1.43	
**DFFu**	+0.32	–1.17	1.49
	+0.81	–1.51	
**6**	+0.36	–1.13	1.49
	+0.81	–1.46	

[a] Scan rate 100 mV s^{-1}. [b] $\Delta E_{redox} = E^1_{ox} - E^1_{red}$

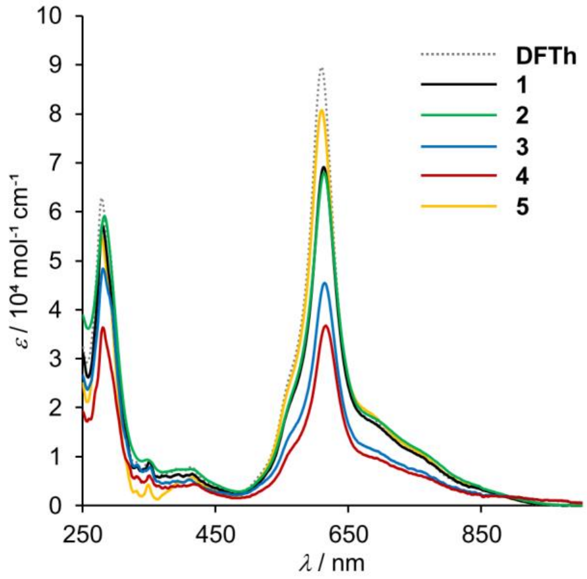

Figure S8. Electronic absorption spectra of **DFTh** and its derivatives **1–5** in CH_2Cl_2.

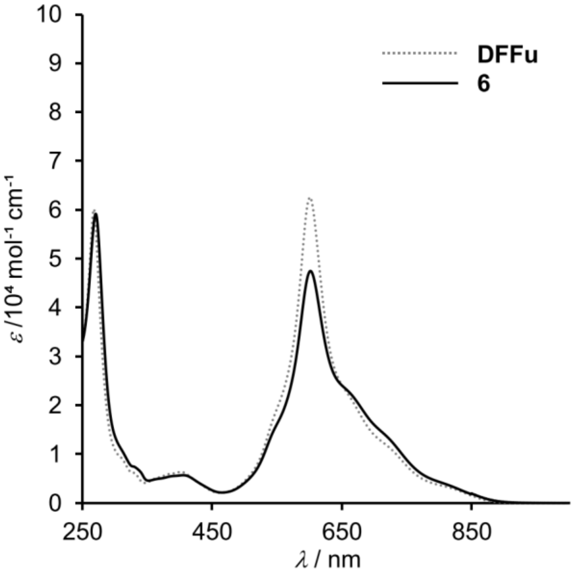

Figure S9. Electronic absorption spectra of **DFFu** and its derivatives **6** in CH_2Cl_2.

3. ^{1}H and ^{13}C Spectral Data

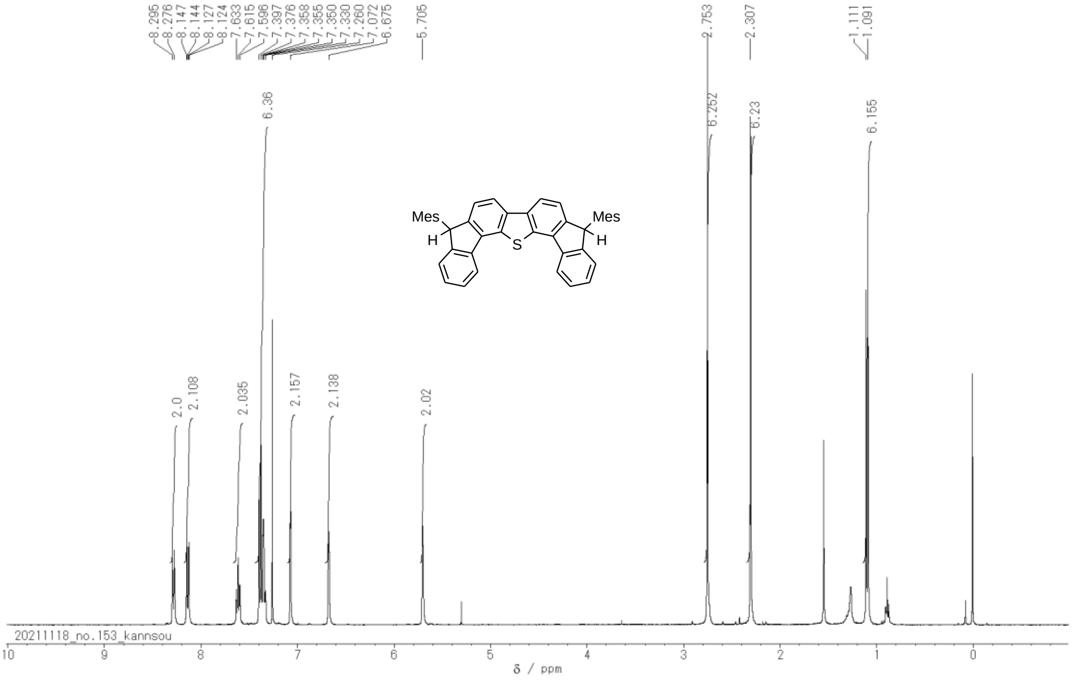

^{1}H NMR spectrum of **DFThH** in $CDCl_3$ solution (400 MHz).

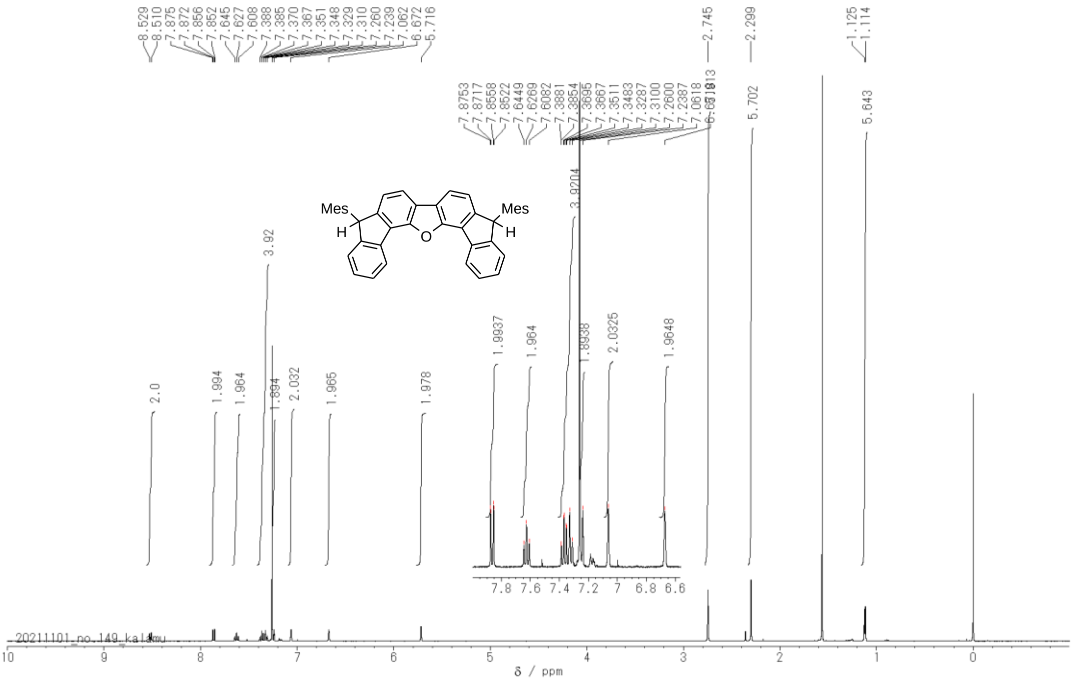

^{1}H NMR spectrum of **DFFuH** in $CDCl_3$ solution (400 MHz).

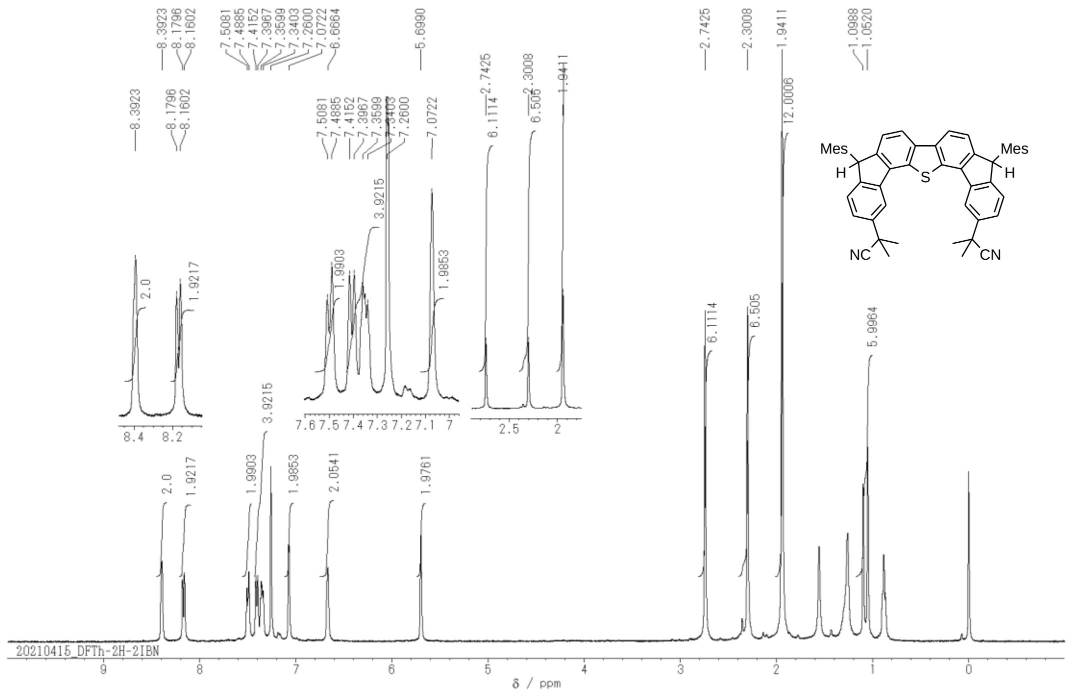

^{1}H NMR spectrum of **1H** in $CDCl_3$ solution (400 MHz).

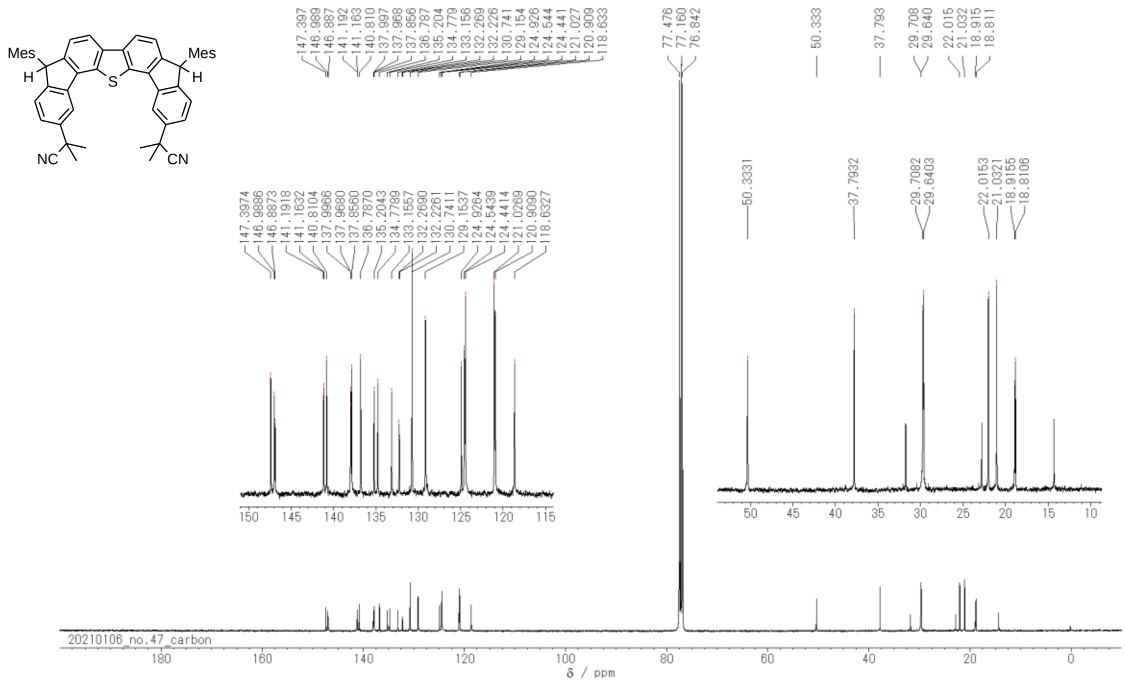

^{13}C NMR spectrum of **1H** in $CDCl_3$ solution (100 MHz).

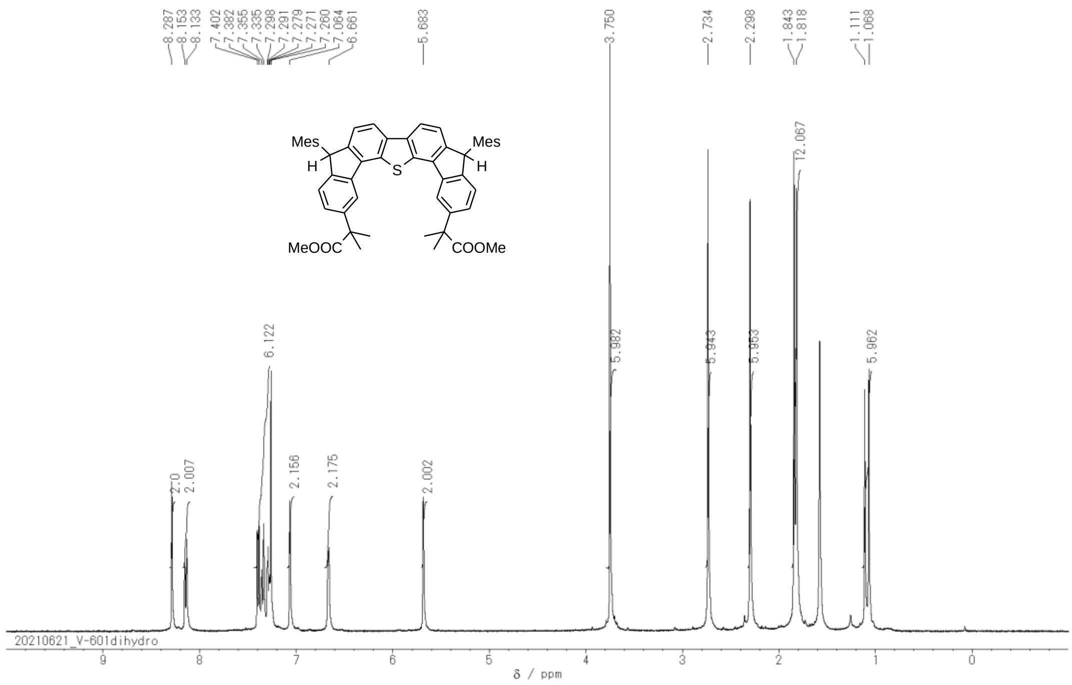

^{1}H NMR spectrum of **2H** in $CDCl_3$ solution (400 MHz).

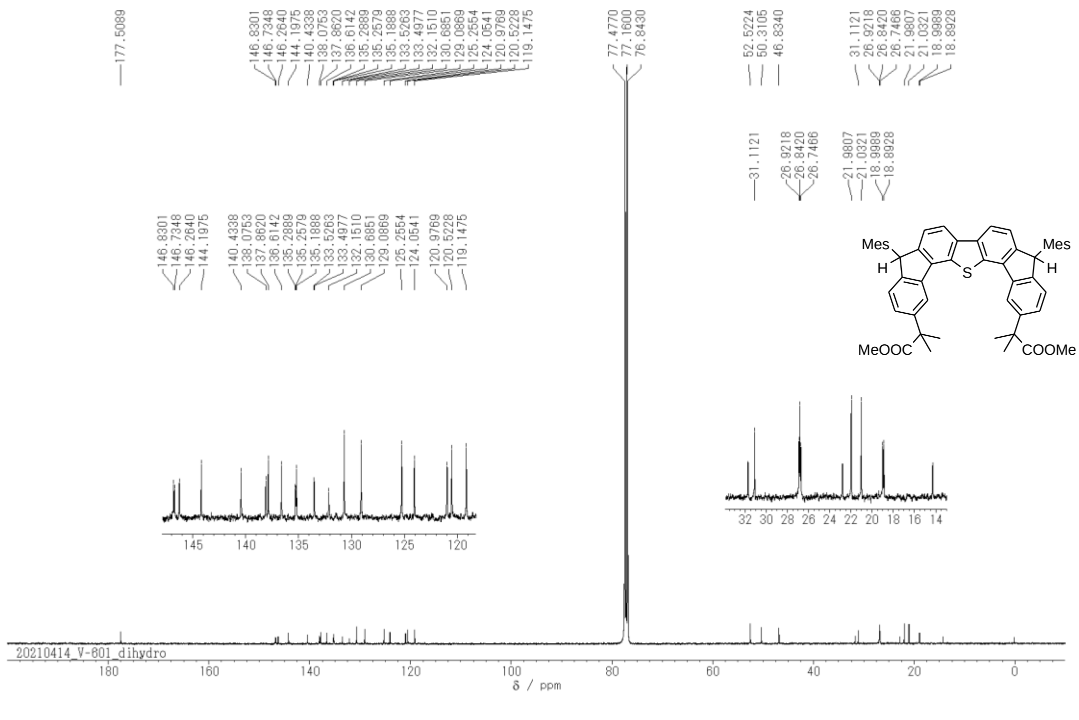

^{13}C NMR spectrum of **2H** in $CDCl_3$ solution (100 MHz).

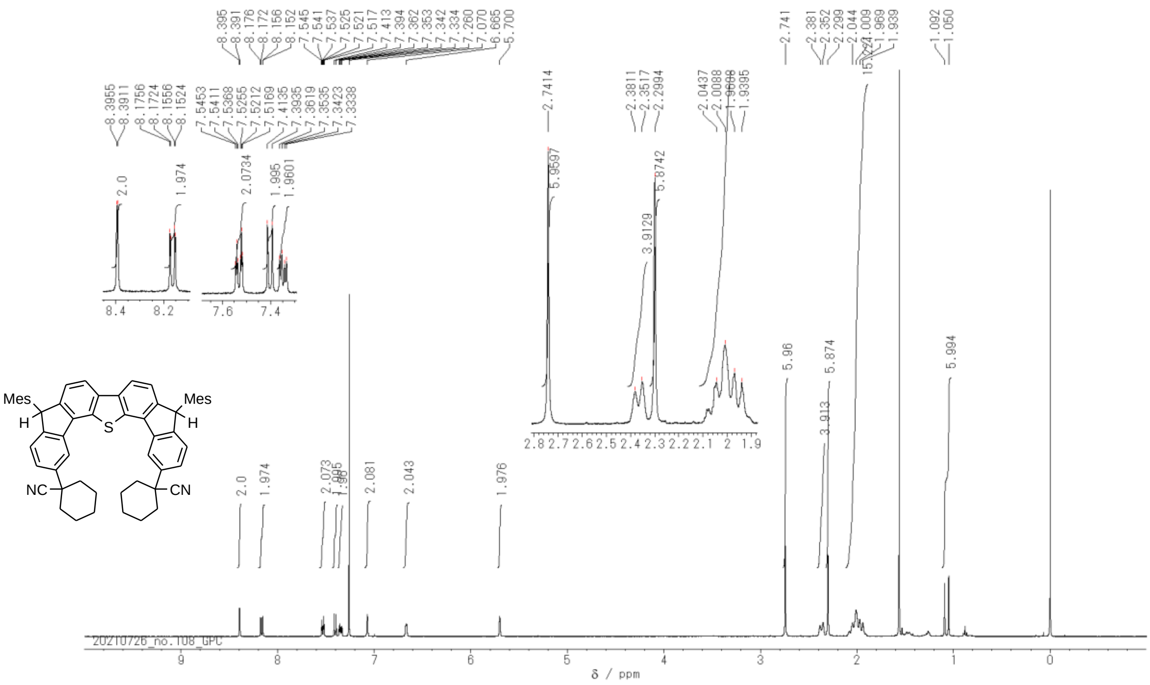

^{1}H NMR spectrum of **3H** in $CDCl_3$ solution (400 MHz).

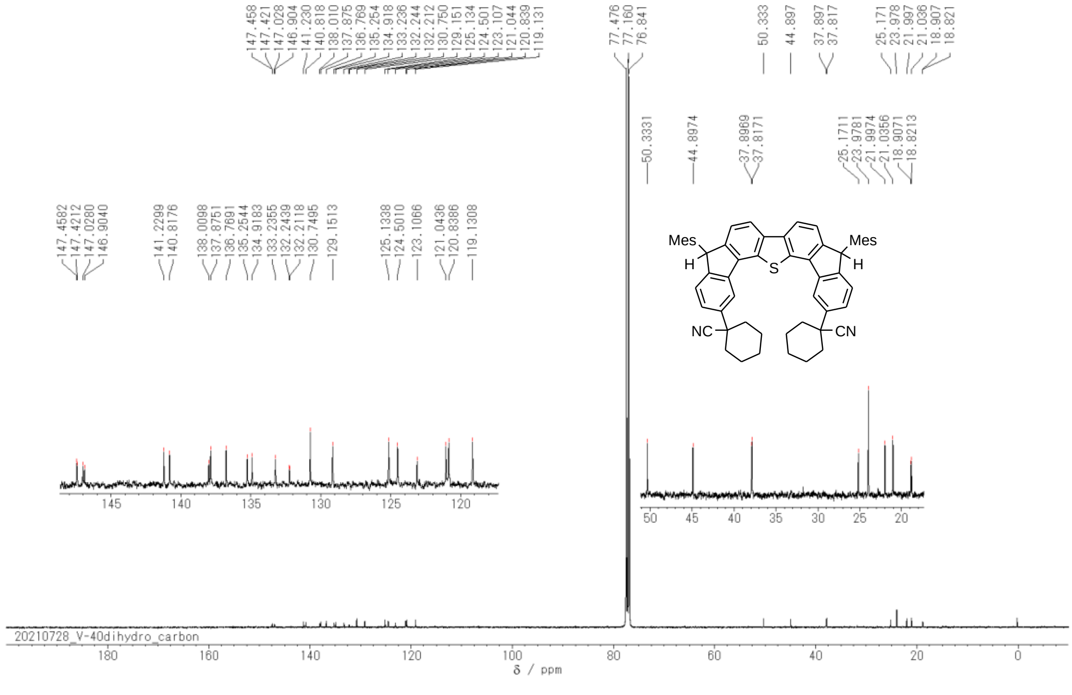

^{13}C NMR spectrum of **3H** in $CDCl_3$ solution (100 MHz).

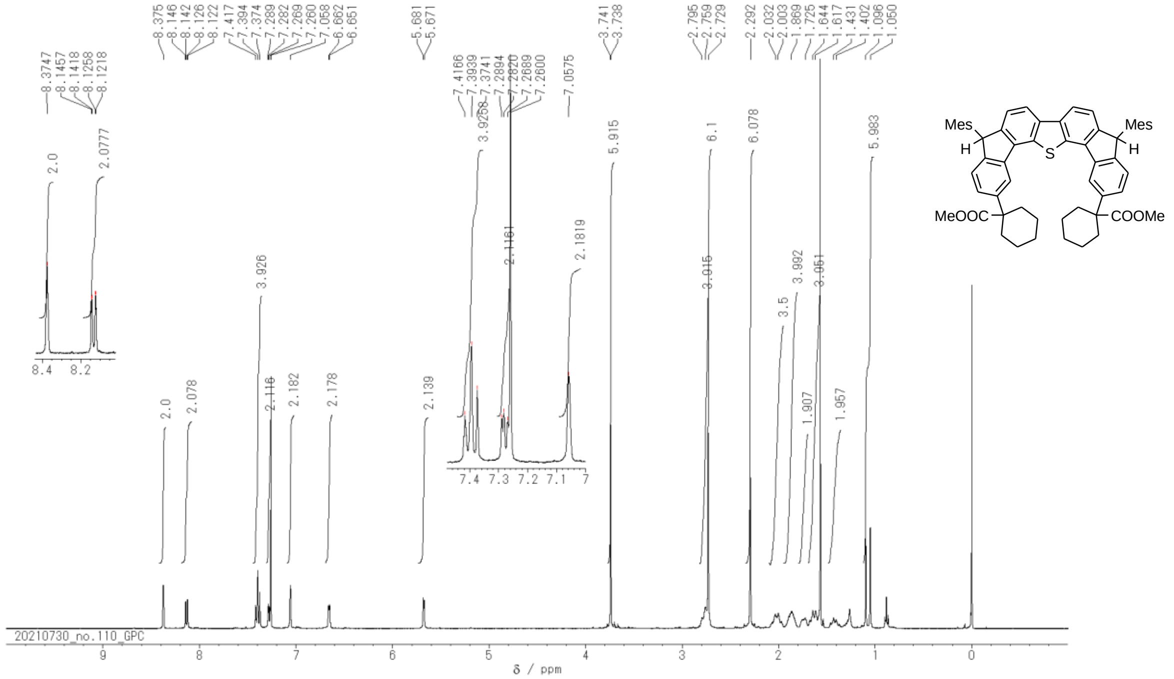

^{1}H NMR spectrum of **4H** in $CDCl_3$ solution (400 MHz).

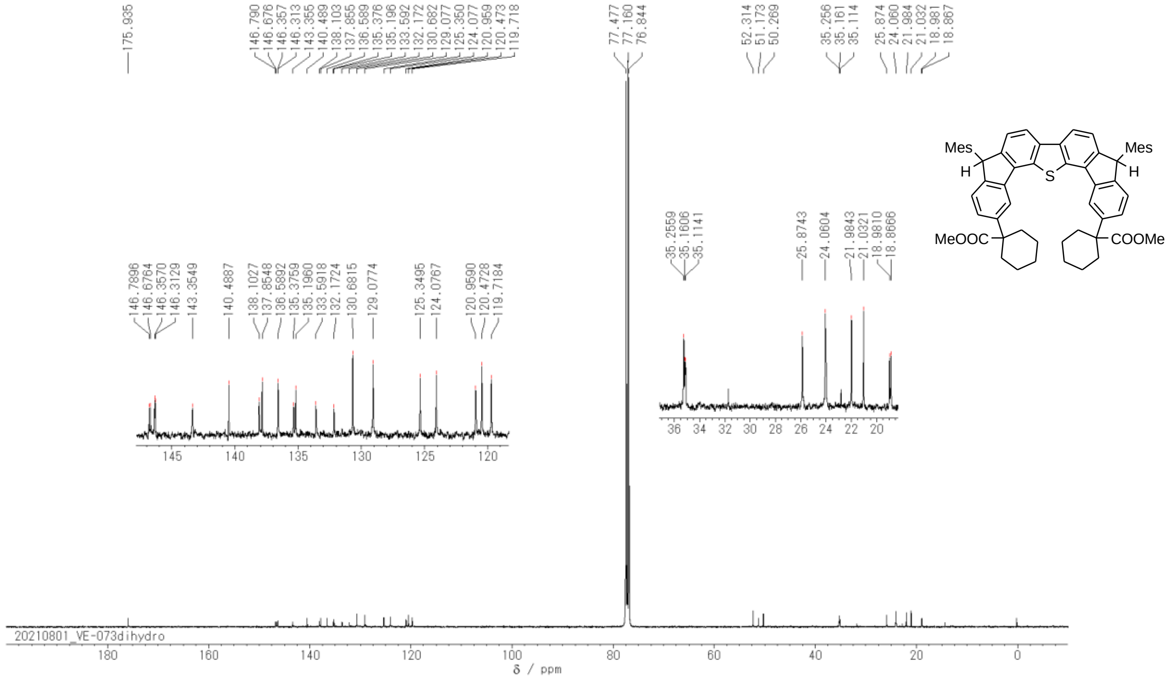

^{13}C NMR spectrum of **4H** in $CDCl_3$ solution (100 MHz).

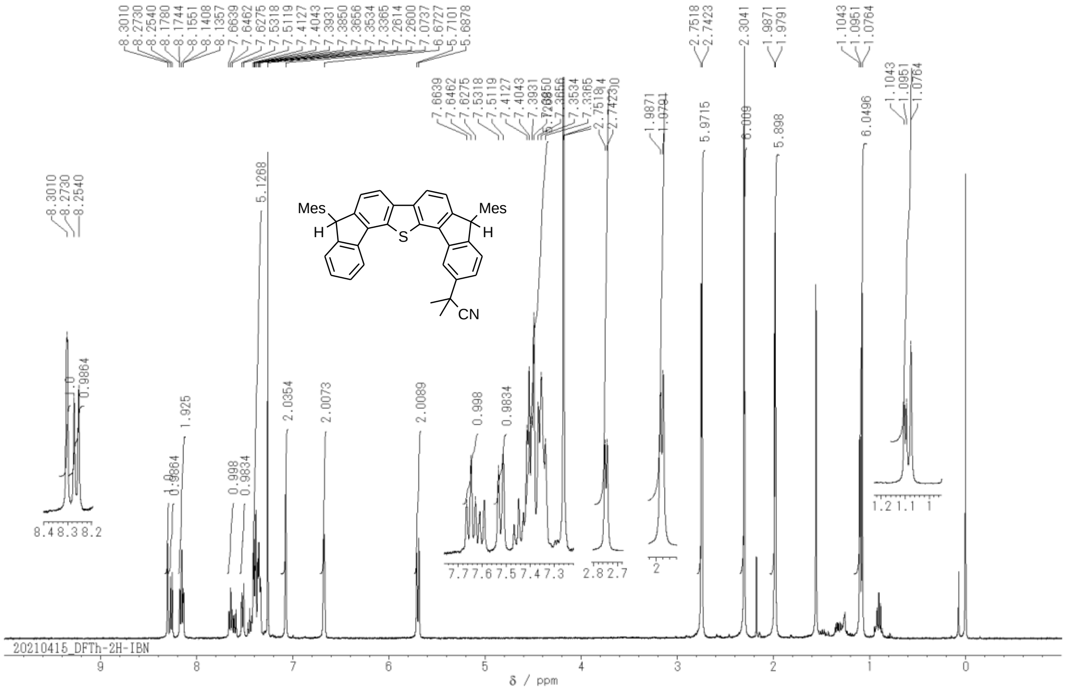

^{1}H NMR spectra of **5H** in $CDCl_3$ solution (400 MHz).

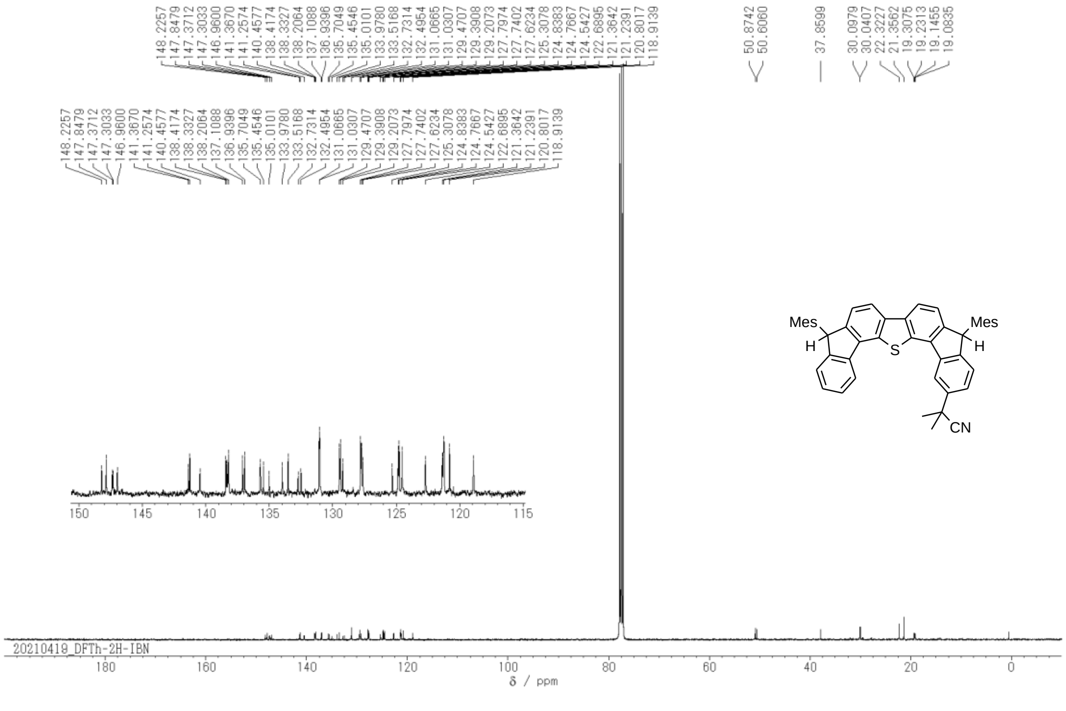

^{13}C NMR spectrum of **5H** in $CDCl_3$ solution (100 MHz).

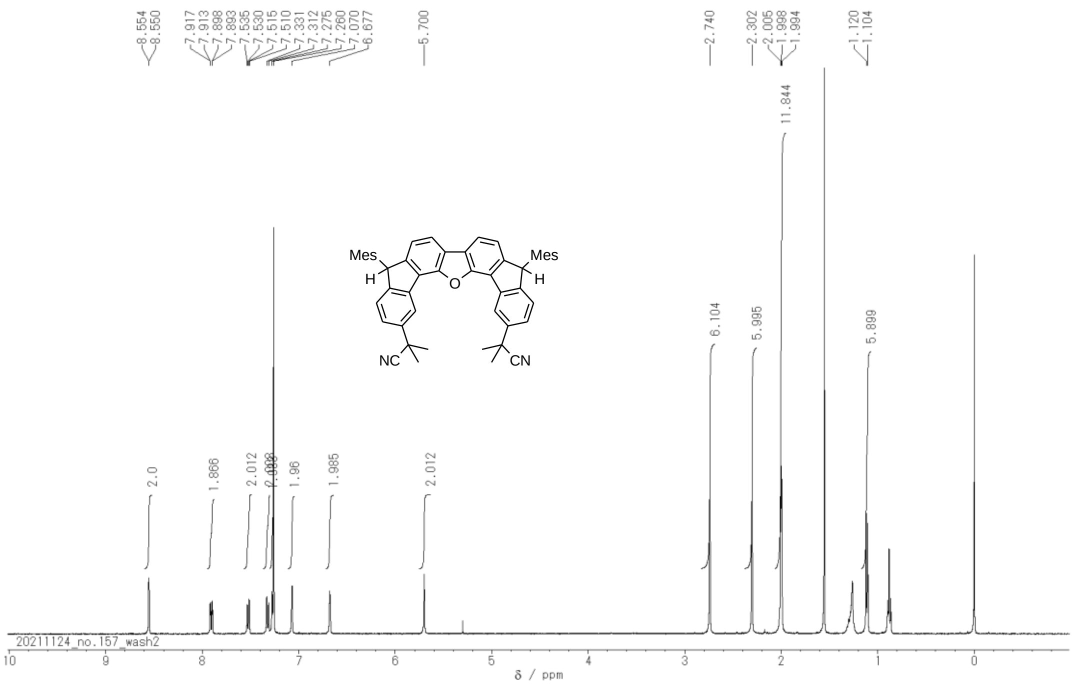

^{1}H NMR spectra of **6H** in $CDCl_3$ solution (400 MHz).

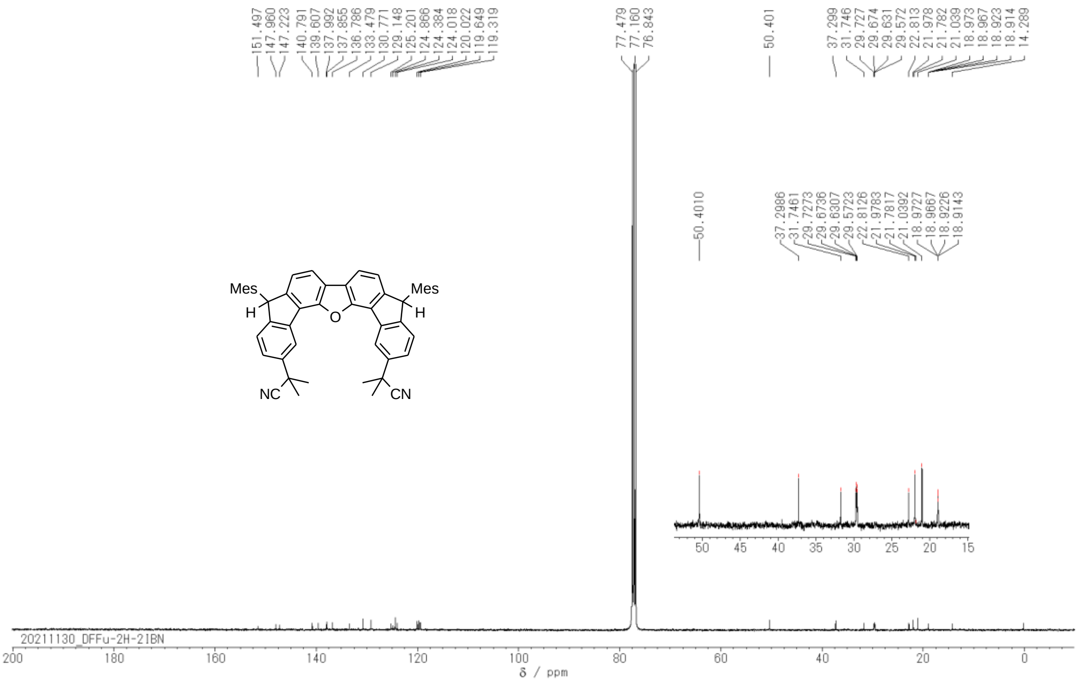

^{13}C NMR spectrum of **6H** in $CDCl_3$ solution (100 MHz).

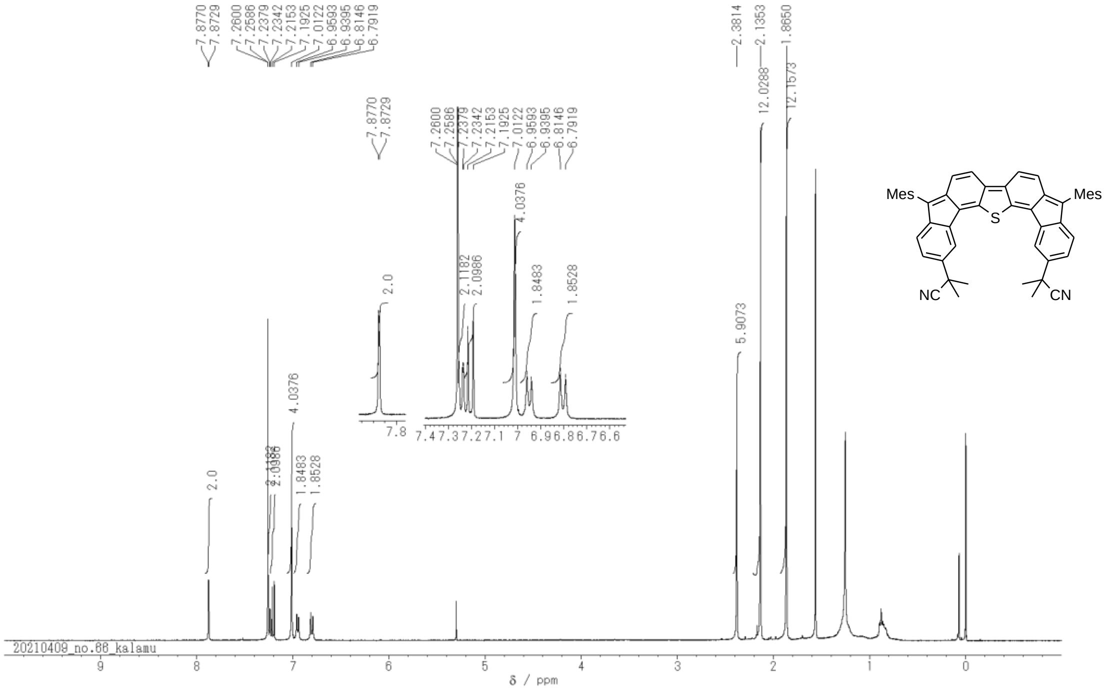

^{1}H NMR spectra of **1** in $CDCl_3$ solution (400 MHz).

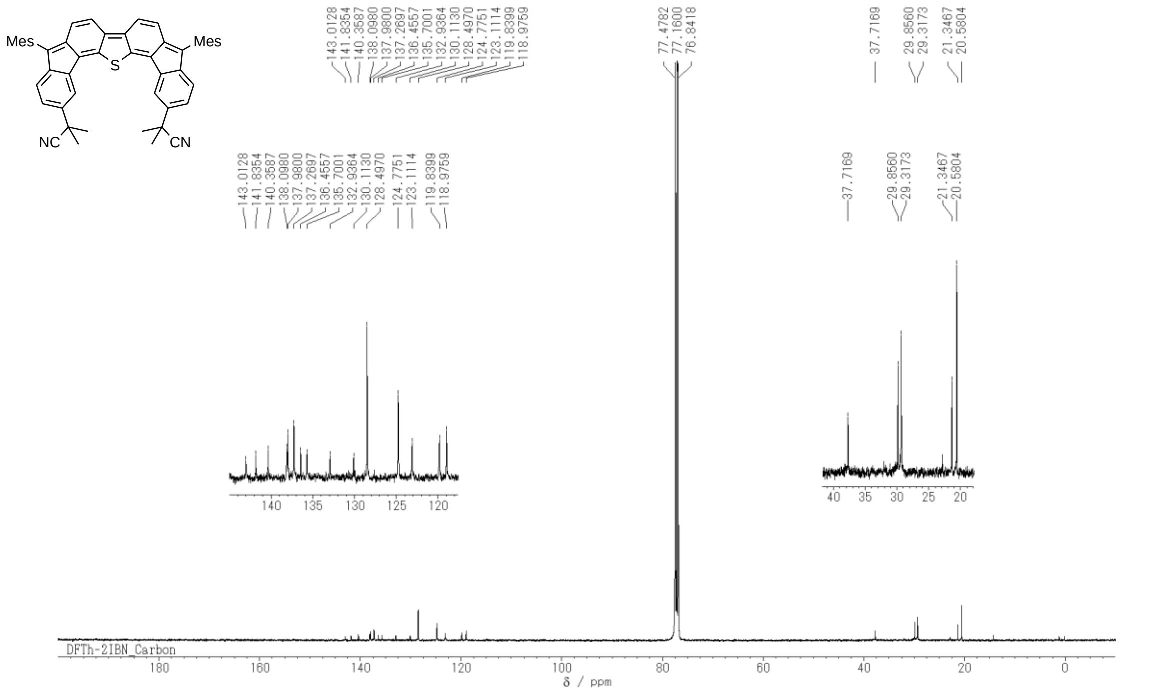

^{13}C NMR spectrum of **1** in $CDCl_3$ solution (100 MHz).

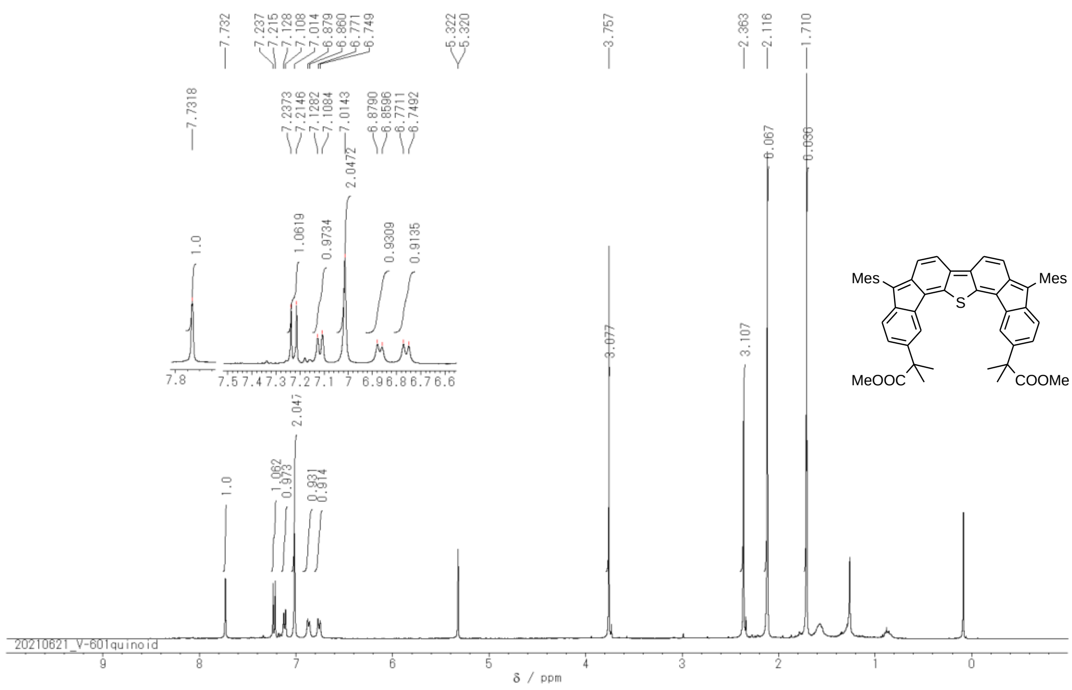

^{1}H NMR spectra of **2** in $CDCl_3/Et_3N$ solution (400 MHz).

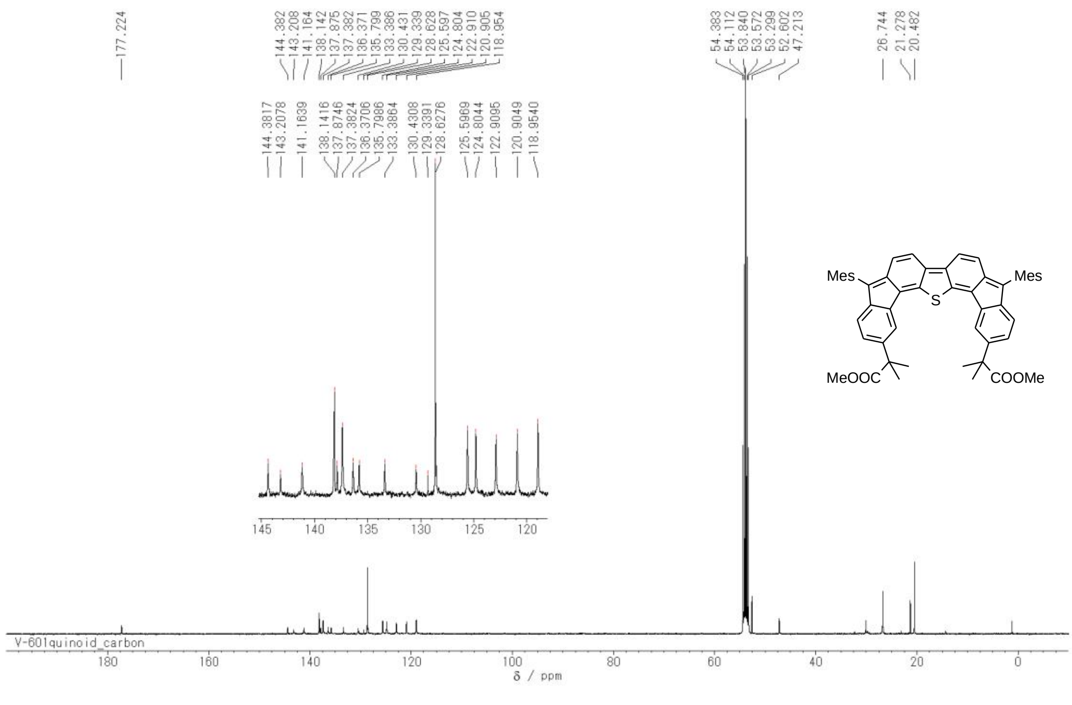

^{13}C NMR spectrum of **2** in CD_2Cl_2 solution (100 MHz).

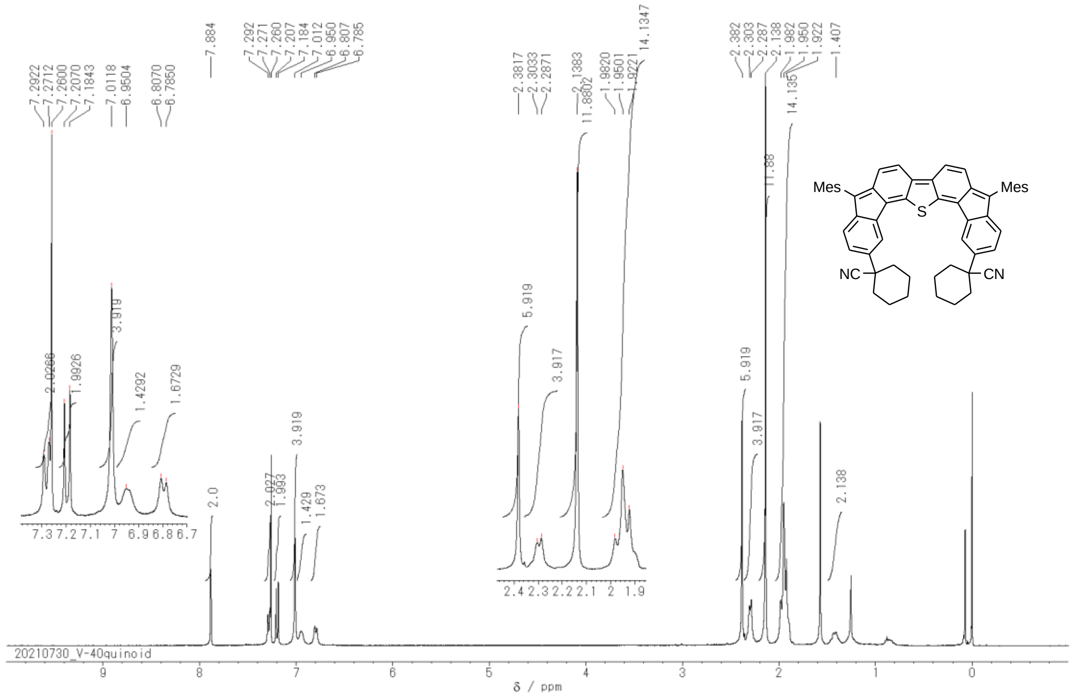

^{1}H NMR spectra of **3** in $CDCl_3/Et_3N$ solution (400 MHz).

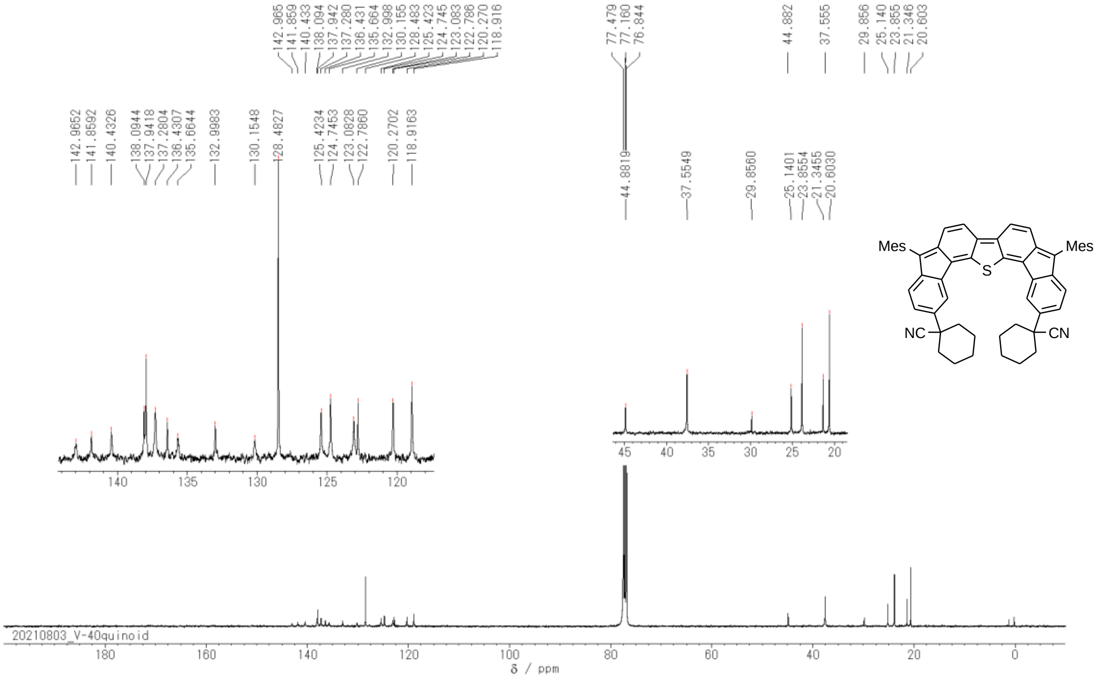

^{13}C NMR spectrum of **3** in CD_2Cl_2 solution (100 MHz).

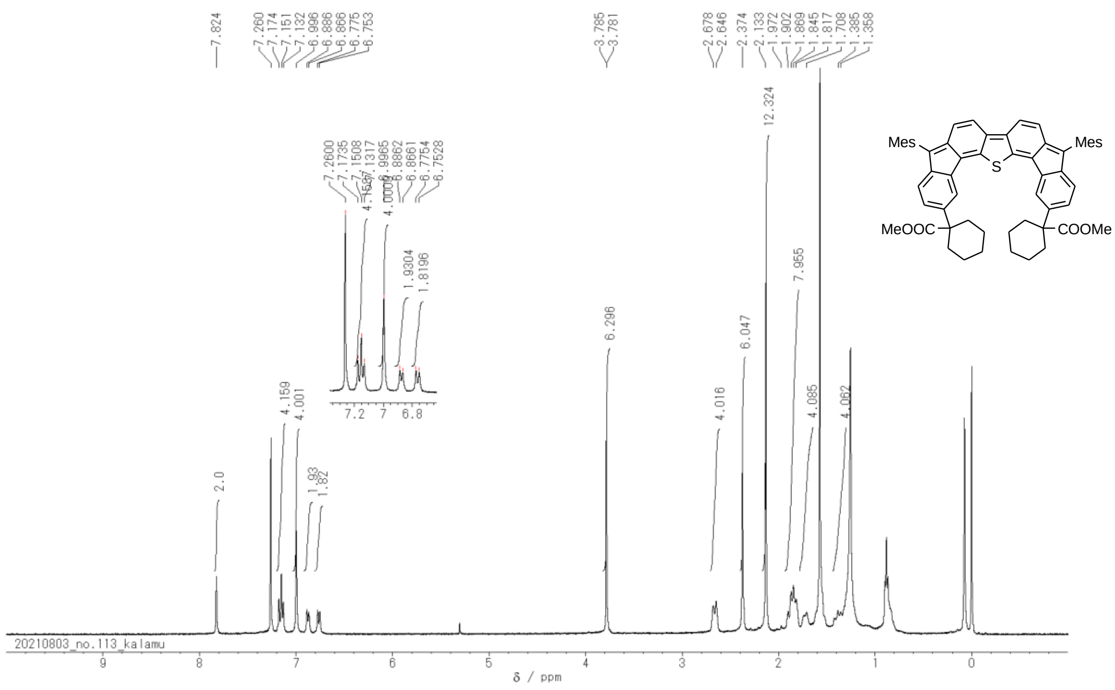

^{1}H NMR spectra of **4** in $CDCl_3$/Et_3N solution (400 MHz).

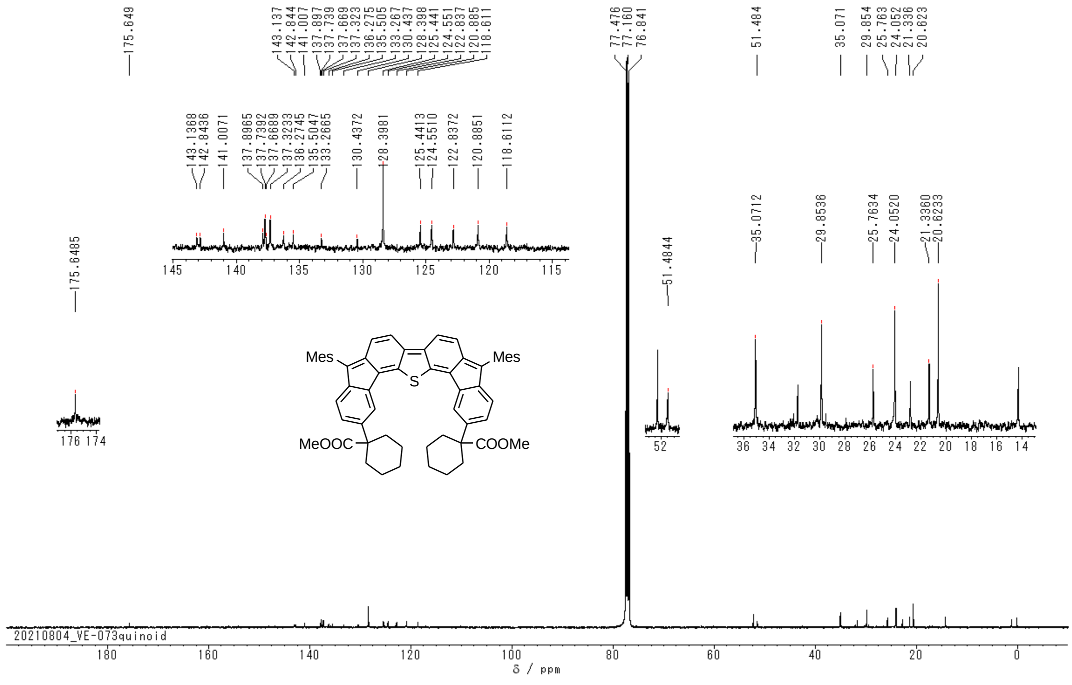

^{13}C NMR spectrum of **4** in CD_2Cl_2 solution (100 MHz).

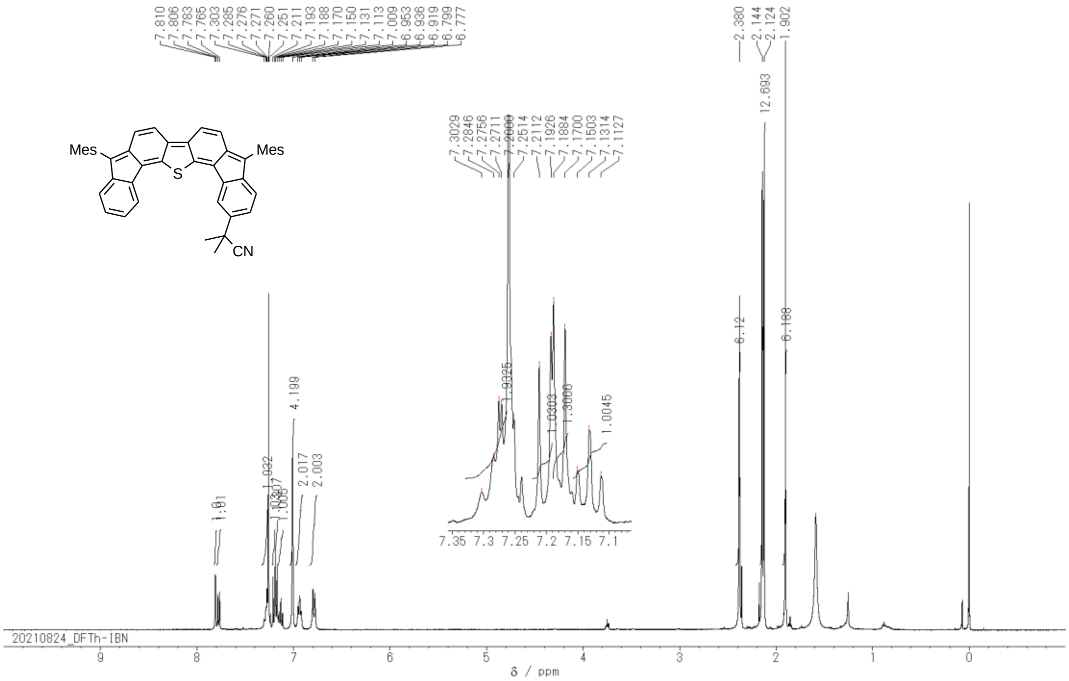

^{1}H NMR spectra of **5** in $CDCl_3$ solution (400 MHz).

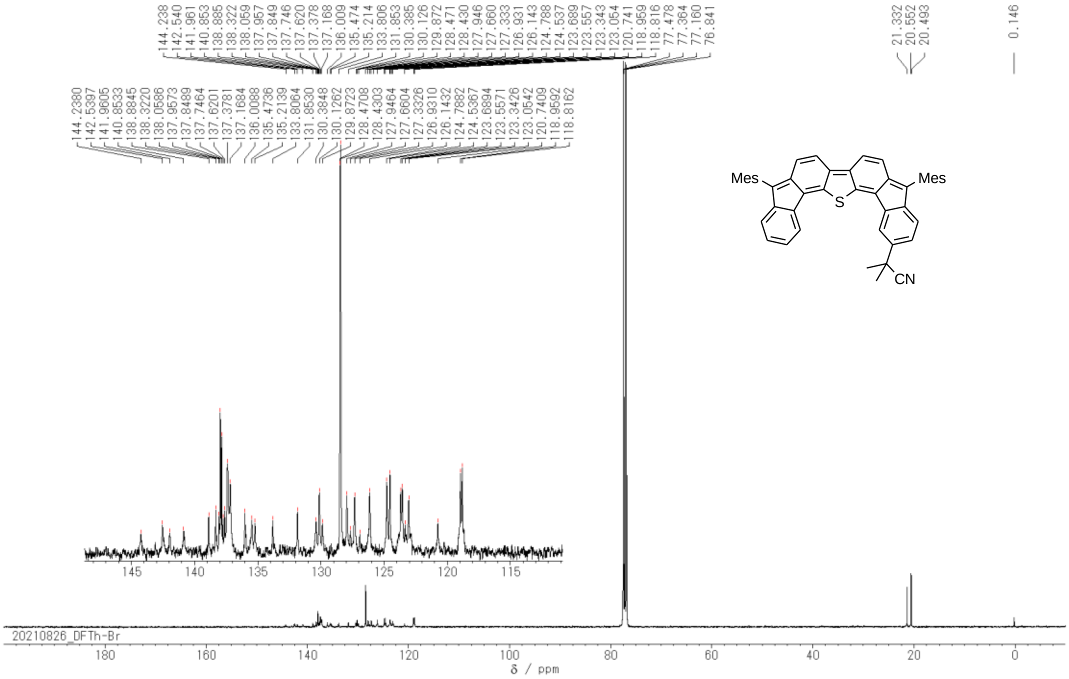

^{13}C NMR spectra of **5** in $CDCl_3$ solution (100 MHz).

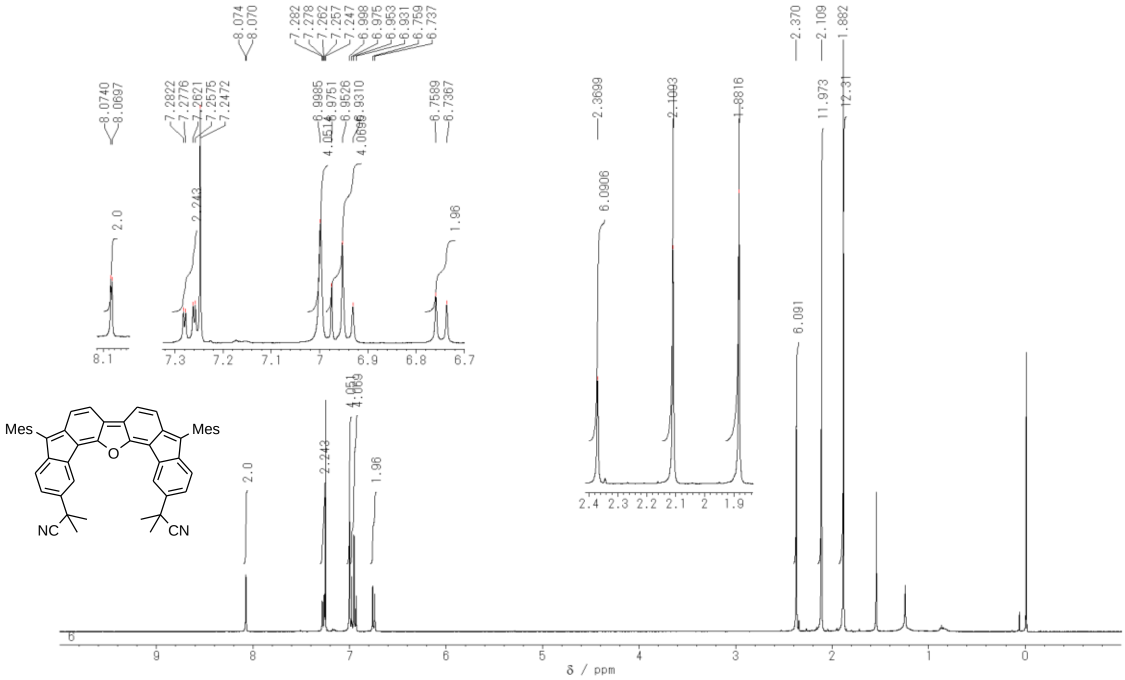

^{1}H NMR spectra of **6** in $CDCl_3$ solution (400 MHz).

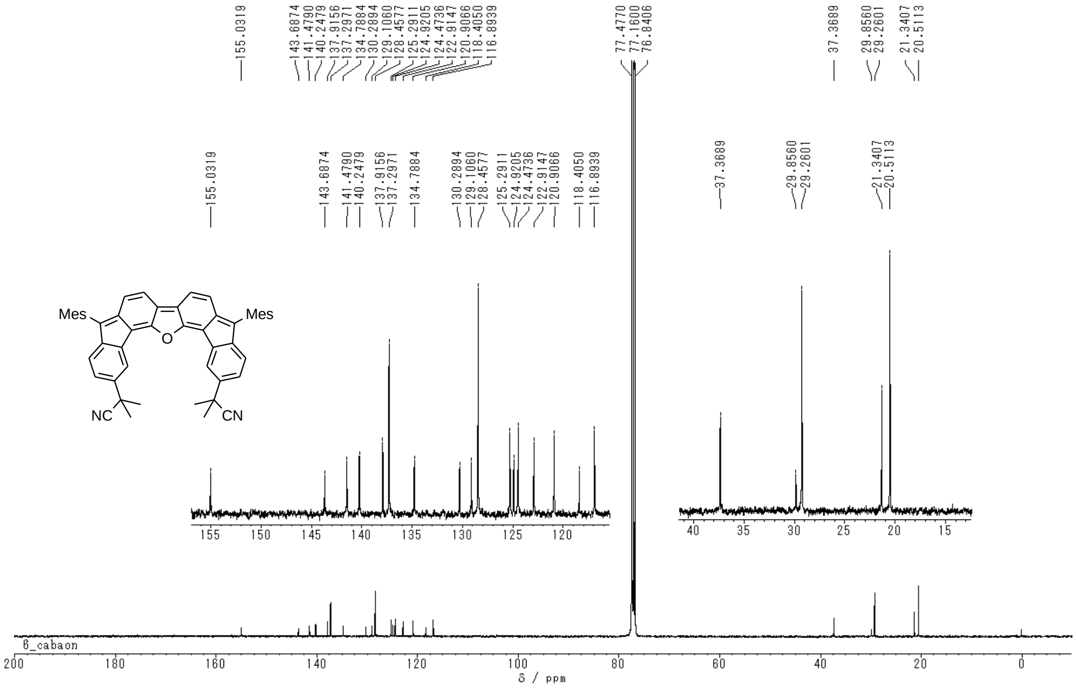

^{13}C NMR spectra of **6** in $CDCl_3$ solution (100 MHz).

4. Theoretical Calculations

All optimized structures for the analysis of reaction mechanisms were obtained using B3LYP functional[9,10] with 6-31G** basis set[11] as implemented in the *Gaussian 16* program package.[11] Using the vibrational frequency analysis, we confirmed that the obtained local minima have no imaginary frequency.

Optimized structure of $\boldsymbol{A}^{cs}$

Atom	x	y	z
C	-0.701391	-0.751037	0.001858
C	-1.262856	0.582203	0.004021
C	1.262470	0.581753	-0.005837
C	0.700537	-0.751282	-0.003066
C	-1.585395	-1.879490	0.001072
C	-2.941732	-1.703599	0.003720
C	-3.495340	-0.385652	0.013109
C	-2.617864	0.777744	0.008806
C	2.617551	0.776811	-0.010504
C	3.494635	-0.386882	-0.014136
C	2.940552	-1.704635	-0.004054
C	1.584150	-1.880039	-0.001512
C	3.474058	1.961378	-0.012230
C	4.822617	1.494421	-0.012816
C	4.821417	0.035526	-0.014379
C	-4.821974	0.037192	0.013529
C	-4.822705	1.496087	0.011809
C	-3.473988	1.962592	0.010511
C	3.203696	3.325658	-0.019927
C	4.276306	4.229282	-0.028755
C	5.598256	3.772806	-0.033965
C	5.882560	2.402992	-0.028855
C	-5.882345	2.405015	0.028330
C	-5.597565	3.774735	0.033145
C	-4.275467	4.230764	0.027125
C	-3.203161	3.326780	0.017855
C	-6.031489	-0.821857	0.023622
C	-6.777272	-1.007268	-1.162520
C	-7.919021	-1.813011	-1.129315
C	-8.347913	-2.441177	0.043143
C	-7.600726	-2.239912	1.206510
C	-6.450395	-1.444946	1.220523
C	-5.690424	-1.248407	2.513270
C	-6.345811	-0.366999	-2.462469
C	-9.568088	-3.331385	0.046427
C	6.031089	-0.823274	-0.023335
C	6.774203	-1.010224	1.164336
C	7.912379	-1.821035	1.134195
C	8.343245	-2.448782	-0.037825
C	7.594494	-2.252133	-1.200901
C	6.447631	-1.452139	-1.217975
C	5.684740	-1.262927	-2.510111
C	6.338879	-0.372609	2.464301
C	9.596557	-3.291591	-0.052326
H	-1.163372	-2.879027	-0.006415

H	-3.610674	-2.558918	-0.004811
H	3.609153	-2.560216	0.005277
H	1.161790	-2.879428	0.006583
H	2.183856	3.698314	-0.021206
H	4.075665	5.296371	-0.034076
H	6.413491	4.490294	-0.044546
H	6.909372	2.048843	-0.040314
H	-6.909282	2.051242	0.040462
H	-6.412557	4.492491	0.044123
H	-4.074465	5.297786	0.032196
H	-2.183196	3.699094	0.018576
H	-8.486689	-1.954567	-2.046520
H	-7.924080	-2.708873	2.133345
H	-4.697946	-1.710324	2.475697
H	-6.233091	-1.690010	3.353281
H	-5.534255	-0.186432	2.729064
H	-7.004001	-0.667114	-3.282022
H	-5.322021	-0.652617	-2.726809
H	-6.360093	0.726279	-2.400815
H	-9.303325	-4.368474	-0.195475
H	-10.304468	-3.004590	-0.693967
H	-10.053828	-3.341423	1.026733
H	8.473954	-1.969374	2.054076
H	7.912218	-2.731107	-2.124555
H	4.693013	-1.726154	-2.468960
H	6.226701	-1.707205	-3.349166
H	5.526639	-0.202121	-2.730139
H	6.352596	0.720758	2.404112
H	6.995473	-0.673290	3.284927
H	5.314666	-0.659227	2.725928
H	9.761911	-3.779980	0.912784
H	9.548494	-4.067885	-0.821780
H	10.483320	-2.680330	-0.262436
S	0.000026	1.821783	-0.001223

*Optimized structure of **A**^{oss}*

Atom	x	y	z
C	-0.726769	-0.765745	0.004061
C	-1.264410	0.550010	0.008815
C	1.263976	0.549585	-0.010586
C	0.725895	-0.765989	-0.005786
C	-1.594733	-1.873987	0.005391
C	-2.972362	-1.691640	0.013074
C	-3.499191	-0.387904	0.024955
C	-2.639502	0.754512	0.018205
C	2.639140	0.753621	-0.019774
C	3.498458	-0.389080	-0.026358
C	2.971186	-1.692646	-0.014419
C	1.593491	-1.874518	-0.006880
C	3.487444	1.945865	-0.018081
C	4.848285	1.497076	-0.017734
C	4.870687	0.058541	-0.024487
C	-4.871280	0.060144	0.023402
C	-4.848422	1.498684	0.017137
C	-3.487432	1.947023	0.017001
C	3.201246	3.305857	-0.021874
C	4.262325	4.224244	-0.025899

C	5.594321	3.789264	-0.030497
C	5.897907	2.427430	-0.029462
C	-5.897720	2.429406	0.029924
C	-5.593658	3.791134	0.031345
C	-4.261519	4.225665	0.026089
C	-3.200762	3.306916	0.021137
C	-6.074466	-0.803341	0.028031
C	-6.821742	-0.984732	-1.157849
C	-7.955027	-1.802578	-1.128766
C	-8.373140	-2.447504	0.038426
C	-7.623804	-2.251132	1.201517
C	-6.482081	-1.444660	1.219702
C	-5.721110	-1.246636	2.511321
C	-6.401712	-0.326869	-2.453011
C	-9.583422	-3.351042	0.036308
C	6.074096	-0.804652	-0.028103
C	6.817156	-0.989725	1.159926
C	7.946900	-1.812472	1.133872
C	8.368504	-2.454764	-0.033584
C	7.619042	-2.260811	-1.196931
C	6.480828	-1.449379	-1.218148
C	5.718321	-1.256282	-2.509607
C	6.391580	-0.336690	2.455715
C	9.612457	-3.311303	-0.043377
H	-1.182520	-2.878283	-0.002970
H	-3.641671	-2.546485	0.005964
H	3.640176	-2.547743	-0.006987
H	1.180954	-2.878678	0.001609
H	2.176955	3.665772	-0.023662
H	4.045688	5.288321	-0.028221
H	6.397138	4.520456	-0.037699
H	6.929342	2.086945	-0.041201
H	-6.929279	2.089312	0.042310
H	-6.396228	4.522587	0.039389
H	-4.044519	5.289667	0.028699
H	-2.176345	3.666474	0.022568
H	-8.523926	-1.941332	-2.045616
H	-7.938638	-2.733399	2.124432
H	-4.703542	-1.646693	2.448744
H	-6.227667	-1.745041	3.341952
H	-5.626106	-0.184235	2.760004
H	-7.041141	-0.649746	-3.278751
H	-5.366031	-0.574656	-2.709015
H	-6.457094	0.765090	-2.391045
H	-9.307458	-4.383141	-0.214217
H	-10.324433	-3.026475	-0.700403
H	-10.067493	-3.374311	1.017212
H	8.508499	-1.959583	2.053912
H	7.929332	-2.751276	-2.117062
H	4.701140	-1.657029	-2.445228
H	6.224783	-1.756444	-3.339239
H	5.622235	-0.194660	-2.761161
H	6.445755	0.755507	2.396913
H	7.028819	-0.660949	3.282603
H	5.355385	-0.586513	2.707671
H	9.776599	-3.790175	0.926667
H	9.552704	-4.095870	-0.803561

H	10.504821	-2.712141	-0.264334
S	-0.000008	1.786960	-0.000946

Optimized structure of ***A***^{T}

Atom	x	y	z
C	-0.726769	-0.765745	0.004061
C	-1.264410	0.550010	0.008815
C	1.263976	0.549585	-0.010586
C	0.725895	-0.765989	-0.005786
C	-1.594733	-1.873987	0.005391
C	-2.972362	-1.691640	0.013074
C	-3.499191	-0.387904	0.024955
C	-2.639502	0.754512	0.018205
C	2.639140	0.753621	-0.019774
C	3.498458	-0.389080	-0.026358
C	2.971186	-1.692646	-0.014419
C	1.593491	-1.874518	-0.006880
C	3.487444	1.945865	-0.018081
C	4.848285	1.497076	-0.017734
C	4.870687	0.058541	-0.024487
C	-4.871280	0.060144	0.023402
C	-4.848422	1.498684	0.017137
C	-3.487432	1.947023	0.017001
C	3.201246	3.305857	-0.021874
C	4.262325	4.224244	-0.025899
C	5.594321	3.789264	-0.030497
C	5.897907	2.427430	-0.029462
C	-5.897720	2.429406	0.029924
C	-5.593658	3.791134	0.031345
C	-4.261519	4.225665	0.026089
C	-3.200762	3.306916	0.021137
C	-6.074466	-0.803341	0.028031
C	-6.821742	-0.984732	-1.157849
C	-7.955027	-1.802578	-1.128766
C	-8.373140	-2.447504	0.038426
C	-7.623804	-2.251132	1.201517
C	-6.482081	-1.444660	1.219702
C	-5.721110	-1.246636	2.511321
C	-6.401712	-0.326869	-2.453011
C	-9.583422	-3.351042	0.036308
C	6.074096	-0.804652	-0.028103
C	6.817156	-0.989725	1.159926
C	7.946900	-1.812472	1.133872
C	8.368504	-2.454764	-0.033584
C	7.619042	-2.260811	-1.196931
C	6.480828	-1.449379	-1.218148
C	5.718321	-1.256282	-2.509607
C	6.391580	-0.336690	2.455715
C	9.612457	-3.311303	-0.043377
H	-1.182520	-2.878283	-0.002970
H	-3.641671	-2.546485	0.005964
H	3.640176	-2.547743	-0.006987
H	1.180954	-2.878678	0.001609
H	2.176955	3.665772	-0.023662
H	4.045688	5.288321	-0.028221
H	6.397138	4.520456	-0.037699
H	6.929342	2.086945	-0.041201

H	-6.929279	2.089312	0.042310
H	-6.396228	4.522587	0.039389
H	-4.044519	5.289667	0.028699
H	-2.176345	3.666474	0.022568
H	-8.523926	-1.941332	-2.045616
H	-7.938638	-2.733399	2.124432
H	-4.703542	-1.646693	2.448744
H	-6.227667	-1.745041	3.341952
H	-5.626106	-0.184235	2.760004
H	-7.041141	-0.649746	-3.278751
H	-5.366031	-0.574656	-2.709015
H	-6.457094	0.765090	-2.391045
H	-9.307458	-4.383141	-0.214217
H	-10.324433	-3.026475	-0.700403
H	-10.067493	-3.374311	1.017212
H	8.508499	-1.959583	2.053912
H	7.929332	-2.751276	-2.117062
H	4.701140	-1.657029	-2.445228
H	6.224783	-1.756444	-3.339239
H	5.622235	-0.194660	-2.761161
H	6.445755	0.755507	2.396913
H	7.028819	-0.660949	3.282603
H	5.355385	-0.586513	2.707671
H	9.776599	-3.790175	0.926667
H	9.552704	-4.095870	-0.803561
H	10.504821	-2.712141	-0.264334
S	-0.000008	1.786960	-0.000946

Optimized structure of ***TS1***

Atom	x	y	z
C	-8.340795	-0.584172	-1.696891
C	-8.065571	0.188678	-0.546398
C	-8.996787	1.160066	-0.114620
C	-10.177750	1.340522	-0.840516
C	-10.466954	0.588615	-1.982473
C	-9.536025	-0.370468	-2.390102
C	-6.809066	-0.024283	0.210321
C	-5.508612	0.434189	-0.157326
C	-4.565407	0.018746	0.843893
C	-5.304329	-0.725640	1.863844
C	-6.676771	-0.739917	1.460153
C	-5.088874	1.190101	-1.274846
C	-3.754096	1.526838	-1.414210
C	-2.806115	1.125396	-0.444885
C	-3.234372	0.369189	0.686059
C	-1.388469	1.384093	-0.424967
C	-0.749255	0.822868	0.714379
S	-1.891538	-0.017928	1.770271
C	0.620227	0.977576	0.910163
C	1.375079	1.692814	-0.064190
C	0.745932	2.266548	-1.182940
C	-0.620453	2.112726	-1.359326
C	1.526013	0.580089	1.988288
C	2.821134	1.056301	1.661646
C	2.794909	1.677256	0.308084
C	3.897673	0.809887	2.511841
C	3.674112	0.119489	3.707409

C	2.393623	-0.340861	4.037447
C	1.313024	-0.120033	3.178733
C	-7.634135	-1.398501	2.240909
C	-7.228324	-2.028881	3.419596
C	-5.885405	-2.009311	3.814706
C	-4.914319	-1.359275	3.038158
C	3.725419	2.792770	-0.097472
C	4.351668	2.845649	-1.366672
C	5.224537	3.900464	-1.664598
C	5.492050	4.928602	-0.764029
C	4.806564	4.907567	0.451694
C	3.923711	3.881130	0.797628
C	4.128814	1.827292	-2.466968
C	3.164334	4.029409	2.100166
C	6.469804	6.030509	-1.093110
C	-8.723606	2.013855	1.103026
C	-11.735651	0.824138	-2.767555
C	-7.377369	-1.649561	-2.169668
Sn	3.767474	-1.420978	-0.878166
C	3.160752	-2.866142	0.676919
C	4.271548	-3.289809	1.647745
C	3.794422	-4.298441	2.704582
C	4.901745	-4.731292	3.670690
C	2.717619	-1.906894	-2.758644
C	3.014415	-3.316650	-3.294476
C	2.267878	-3.640015	-4.599087
C	2.563107	-5.045206	-5.133018
C	5.947844	-1.550476	-1.235239
C	6.823865	-0.719580	-0.286285
C	8.327130	-0.845443	-0.578306
C	9.198990	-0.015264	0.369110
H	-3.427822	2.106119	-2.272377
H	-5.817518	1.504966	-2.015759
H	1.325442	2.843625	-1.896051
H	-1.108527	2.562167	-2.218570
H	0.326354	-0.484922	3.446054
H	2.237235	-0.874997	4.969838
H	4.501831	-0.060368	4.387320
H	4.892888	1.161915	2.254688
H	-8.674504	-1.419303	1.929128
H	-7.961239	-2.541853	4.035333
H	-5.588660	-2.505834	4.733737
H	-3.877303	-1.360233	3.359899
H	-10.889063	2.092047	-0.504601
H	-9.746723	-0.975633	-3.269277
H	-6.424963	-1.219060	-2.496974
H	-7.798443	-2.210477	-3.008234
H	-7.142414	-2.359419	-1.369520
H	-9.512060	2.758244	1.241879
H	-7.768917	2.543010	1.012813
H	-8.663338	1.411681	2.015736
H	-11.589612	1.593272	-3.536521
H	-12.549886	1.164272	-2.120573
H	-12.065354	-0.085643	-3.278158
H	5.699462	3.917178	-2.643295
H	4.941883	5.730524	1.150265
H	2.121015	3.719039	2.001467

H	3.174378	5.076111	2.416640
H	3.597747	3.434294	2.909562
H	4.860454	1.012953	-2.419157
H	4.253360	2.306208	-3.443026
H	3.133880	1.382142	-2.441821
H	6.586613	6.153330	-2.173840
H	6.148443	6.989493	-0.674825
H	7.463184	5.814787	-0.679998
H	3.291602	0.416903	-0.362702
H	6.219364	-2.611894	-1.169280
H	6.125907	-1.252631	-2.275939
H	1.642659	-1.785251	-2.581650
H	2.996445	-1.151931	-3.503217
H	2.759918	-3.741140	0.148745
H	2.325169	-2.423076	1.228142
H	5.113469	-3.728861	1.094018
H	4.672205	-2.406763	2.162610
H	2.964803	-3.853413	3.269709
H	3.380171	-5.181719	2.199519
H	4.531096	-5.446566	4.412386
H	5.731494	-5.207961	3.136053
H	5.311396	-3.871935	4.213925
H	4.093517	-3.435382	-3.467485
H	2.746640	-4.070993	-2.541097
H	1.188489	-3.528388	-4.430128
H	2.535027	-2.892887	-5.358631
H	2.017681	-5.244048	-6.061379
H	3.631408	-5.174271	-5.341474
H	2.274120	-5.814022	-4.407312
H	6.538791	0.340069	-0.344807
H	6.639446	-1.021458	0.754018
H	8.618118	-1.902846	-0.515595
H	8.517414	-0.540158	-1.616337
H	10.263017	-0.123922	0.134669
H	8.951200	1.050347	0.303879
H	9.055934	-0.323229	1.411270

*Optimized structure of **B***

Atom	x	y	z
S	0.063511	1.746695	0.134676
C	-1.222297	0.534595	0.192305
C	-0.713263	-0.774740	0.414081
C	0.732176	-0.785958	0.539822
C	1.294664	0.508029	0.410460
C	-2.586647	0.750683	0.046608
C	-3.469195	-0.374505	0.124935
C	-2.967698	-1.674230	0.337430
C	-1.601719	-1.866961	0.482728
C	1.576595	-1.888717	0.759164
C	2.954307	-1.711886	0.856129
C	3.494326	-0.431681	0.726755
C	2.677579	0.694350	0.497613
C	-3.405442	1.940186	-0.191512
C	-4.768240	1.506990	-0.257620
C	-4.819000	0.078170	-0.062405
C	4.950979	-0.004220	0.861830
C	4.874507	1.483138	0.538577

C	3.530152	1.883605	0.380028
C	5.906067	2.408733	0.448373
C	5.593247	3.749977	0.199357
C	4.262609	4.152458	0.050247
C	3.221188	3.225726	0.139189
C	-3.092389	3.285954	-0.341375
C	-4.130383	4.205644	-0.556662
C	-5.464838	3.785719	-0.617187
C	-5.794738	2.437289	-0.465875
C	6.005262	-0.836755	0.135793
C	7.013860	-1.484478	0.883627
C	7.981618	-2.249626	0.221103
C	7.988281	-2.395798	-1.165532
C	6.982199	-1.751619	-1.889332
C	5.995033	-0.977576	-1.272425
C	-6.037309	-0.764822	-0.054961
C	-6.671727	-1.104196	-1.271290
C	-7.821827	-1.898221	-1.239857
C	-8.364864	-2.367669	-0.040811
C	-7.725813	-2.016572	1.151344
C	-6.572228	-1.227070	1.168655
C	-6.113923	-0.639190	-2.597590
C	-9.590546	-3.250250	-0.035311
C	-5.932318	-0.858250	2.488175
C	7.096080	-1.389788	2.395121
C	9.057279	-3.200946	-1.865298
C	4.947794	-0.322658	-2.144193
H	-1.210643	-2.866223	0.647575
H	-3.651547	-2.516374	0.382409
H	3.605645	-2.565524	1.018442
H	1.150429	-2.882969	0.852230
H	2.193822	3.552866	0.016179
H	4.034706	5.196942	-0.140970
H	6.390228	4.483573	0.120467
H	6.940269	2.094183	0.557943
H	-6.829820	2.110056	-0.504693
H	-6.250536	4.517263	-0.780947
H	-3.893187	5.258674	-0.675256
H	-2.065146	3.634104	-0.292838
H	-8.303767	-2.158848	-2.179715
H	-8.138776	-2.360738	2.097140
H	-4.925767	-1.279381	2.582879
H	-6.530257	-1.224769	3.326713
H	-5.829701	0.227095	2.592980
H	-6.685832	-1.058887	-3.429309
H	-5.068055	-0.941222	-2.718410
H	-6.137929	0.451886	-2.688228
H	-9.316159	-4.310683	-0.101367
H	-10.245272	-3.031982	-0.884335
H	-10.171622	-3.122929	0.882988
H	8.750374	-2.744738	0.810139
H	6.960298	-1.853415	-2.972263
H	3.940310	-0.674707	-1.902244
H	5.135267	-0.544001	-3.198059
H	4.942793	0.764973	-2.025589
H	7.224466	-0.357806	2.741616
H	7.949463	-1.963375	2.765507

H	6.200698	-1.786762	2.887410
H	9.534673	-3.910649	-1.183472
H	8.645958	-3.765257	-2.708300
H	9.845471	-2.551467	-2.266299
H	5.176574	-0.063199	1.933560

Optimized structure of ***TS2^m^***

Atom	x	y	z
C	-3.593262	-4.115660	0.644003
C	-4.074591	-2.781828	0.520646
C	-5.444927	-2.589407	0.218801
C	-6.258750	-3.698683	-0.044865
C	-5.779845	-5.005985	-0.004465
C	-4.446739	-5.186967	0.367042
C	-3.130515	-1.641871	0.800905
C	-1.749932	-1.594023	0.264329
C	-0.922164	-0.873465	1.160748
C	-1.714362	-0.527947	2.340967
C	-3.027847	-1.038477	2.146630
C	-1.263344	-2.036530	-0.973361
C	0.062875	-1.797296	-1.317212
C	0.913826	-1.100929	-0.438428
C	0.407692	-0.638688	0.800246
S	1.636644	0.207831	1.747064
C	2.843956	-0.049174	0.481756
C	2.316115	-0.761659	-0.621811
C	4.182049	0.358989	0.491854
C	4.985905	0.053253	-0.624388
C	4.478639	-0.647712	-1.718897
C	3.147394	-1.057479	-1.715381
C	4.996763	1.071338	1.483609
C	6.305567	1.202493	0.970885
C	6.378658	0.646582	-0.446268
C	7.301400	1.819685	1.716761
C	6.988064	2.314295	2.988028
C	5.691080	2.194365	3.496163
C	4.685813	1.573683	2.750809
C	-3.986911	-0.911396	3.153545
C	-3.645193	-0.253583	4.337835
C	-2.359414	0.270529	4.519024
C	-1.384971	0.132971	3.526367
C	7.575759	-0.231348	-0.804194
C	8.466635	0.188979	-1.816977
C	9.567811	-0.611617	-2.144841
C	9.819598	-1.823444	-1.502848
C	8.931325	-2.222245	-0.501387
C	7.818652	-1.457115	-0.139742
C	8.284351	1.490024	-2.574831
C	6.916827	-1.976514	0.956849
C	10.996207	-2.687613	-1.889574
C	-6.117465	-1.233219	0.165133
C	-6.667632	-6.181195	-0.334976
C	-2.199589	-4.448745	1.136115
Sn	-3.696110	1.201713	-1.097829
C	-5.204334	2.578413	-0.249915
C	-5.069465	2.877916	1.250330
C	-6.163731	3.816589	1.782007

C	-6.024599	4.120762	3.277075
C	-1.729168	2.186583	-1.262121
C	-1.316016	3.070599	-0.077551
C	0.062864	3.722902	-0.264486
C	0.475000	4.611240	0.913353
C	-4.339657	0.633257	-3.134362
C	-4.554691	1.825119	-4.080731
C	-4.987162	1.408090	-5.495877
C	-5.202027	2.596334	-6.438611
H	2.749000	-1.609899	-2.561066
H	5.121383	-0.883608	-2.561886
H	-1.917424	-2.574865	-1.653066
H	0.446531	-2.150835	-2.269574
H	-0.386199	0.524900	3.691070
H	-2.111855	0.779792	5.445619
H	-4.382657	-0.153272	5.128815
H	-4.978929	-1.333674	3.025721
H	8.310724	1.906612	1.323998
H	7.758470	2.793945	3.584680
H	5.461939	2.584347	4.483532
H	3.685206	1.483961	3.161156
H	9.107650	-3.160389	0.020547
H	10.246775	-0.273457	-2.924436
H	7.336386	1.522794	-3.124321
H	9.086156	1.618694	-3.306300
H	8.304955	2.364302	-1.914029
H	7.299206	-2.922113	1.349849
H	5.898742	-2.151164	0.595574
H	6.842981	-1.271915	1.790549
H	11.451383	-3.159736	-1.013097
H	11.769090	-2.106221	-2.400586
H	10.691186	-3.494232	-2.567990
H	-7.306639	-3.526147	-0.281044
H	-4.058224	-6.197914	0.470153
H	-1.443220	-4.355102	0.351014
H	-2.172382	-5.479321	1.500871
H	-1.889747	-3.794967	1.955469
H	-5.699586	-0.521234	0.877443
H	-7.183758	-1.339485	0.386216
H	-6.043834	-0.785472	-0.832464
H	-7.723815	-5.941725	-0.180351
H	-6.551700	-6.482539	-1.383700
H	-6.423355	-7.053266	0.279610
H	-3.580305	-0.418535	-0.019414
H	-5.145309	3.509558	-0.828593
H	-6.189738	2.146761	-0.464221
H	-3.579064	-0.042997	-3.542455
H	-5.261785	0.045353	-3.053249
H	-1.760897	2.780548	-2.185432
H	-0.987264	1.397485	-1.426101
H	-2.062369	3.861973	0.079782
H	-1.304041	2.478077	0.847041
H	0.813474	2.935132	-0.409697
H	0.057675	4.315660	-1.189722
H	1.458055	5.064611	0.748903
H	-0.244170	5.423842	1.069620
H	0.528100	4.032986	1.842595

H	-5.313711	2.503361	-3.665965
H	-3.632160	2.418065	-4.156276
H	-4.228772	0.734742	-5.917603
H	-5.911700	0.818861	-5.428274
H	-5.508874	2.266334	-7.436557
H	-5.979031	3.269188	-6.058026
H	-4.283972	3.184373	-6.551212
H	-5.092829	1.943495	1.827507
H	-4.088148	3.325601	1.456693
H	-6.141628	4.755259	1.211557
H	-7.147461	3.367674	1.587208
H	-6.821320	4.784928	3.628146
H	-6.069016	3.202447	3.873597
H	-5.066642	4.606642	3.494654
H	6.412538	1.521795	-1.106314

Optimized structure of ***TS2***^{r}

Atom	x	y	z
C	-4.301607	-3.098041	-2.304332
C	-4.516963	-1.993130	-1.433265
C	-5.798119	-1.389396	-1.420989
C	-6.777221	-1.823415	-2.323918
C	-6.552388	-2.851929	-3.235890
C	-5.311499	-3.488961	-3.187584
C	-3.406267	-1.573588	-0.506213
C	-2.000009	-1.431766	-0.951716
C	-1.133049	-1.706769	0.134923
C	-1.945365	-2.132030	1.274741
C	-3.308943	-2.103753	0.870360
C	-1.499878	-0.975917	-2.179101
C	-0.128095	-0.822341	-2.349399
C	0.758566	-1.105535	-1.293485
C	0.242790	-1.544223	-0.050230
S	1.521251	-1.839645	1.133672
C	2.763561	-1.371916	-0.033568
C	2.209578	-1.006996	-1.284211
C	4.147005	-1.333989	0.170874
C	4.973274	-0.935273	-0.898683
C	4.440896	-0.573388	-2.136507
C	3.061500	-0.605896	-2.327187
C	4.987777	-1.623388	1.338810
C	6.335358	-1.401411	0.981381
C	6.439649	-1.034490	-0.494053
C	7.353694	-1.576178	1.909514
C	7.024744	-1.978738	3.209004
C	5.692208	-2.207609	3.565010
C	4.663910	-2.032957	2.635797
C	-4.310119	-2.529945	1.745523
C	-3.955027	-2.956080	3.027858
C	-2.614991	-2.960396	3.434146
C	-1.601914	-2.554339	2.560884
C	7.360820	0.121954	-0.876006
C	7.138930	1.434149	-0.394309
C	8.010373	2.458803	-0.774864
C	9.102345	2.237418	-1.617297
C	9.304494	0.939841	-2.085707
C	8.458663	-0.118747	-1.732032

C	5.985175	1.776603	0.520636
C	8.766451	-1.492300	-2.296822
C	10.041693	3.360977	-1.986079
C	-6.202381	-0.280977	-0.470989
C	-7.610388	-3.271321	-4.227488
C	-3.039465	-3.935794	-2.279479
Sn	-3.276021	1.827969	0.146854
C	-4.551453	2.472637	1.833953
C	-4.468905	1.611485	3.102943
C	-5.404705	2.091272	4.223359
C	-5.316243	1.236168	5.491466
C	-1.149842	2.255667	0.549756
C	-0.665512	1.984613	1.980599
C	0.822767	2.308925	2.184320
C	1.308169	2.044317	3.612839
C	-3.866282	2.975743	-1.647617
C	-3.764870	4.499967	-1.477687
C	-4.165431	5.281175	-2.739773
C	-4.063893	6.800253	-2.569625
H	2.640952	-0.319075	-3.286320
H	5.097127	-0.256433	-2.941674
H	-2.181699	-0.756742	-2.995618
H	0.263381	-0.482641	-3.303611
H	-0.565830	-2.585149	2.883153
H	-2.357520	-3.293883	4.434998
H	-4.725761	-3.293760	3.714524
H	-5.348264	-2.547373	1.428165
H	8.388445	-1.391908	1.634008
H	7.810431	-2.113424	3.946664
H	5.451894	-2.521106	4.576670
H	3.634053	-2.209505	2.928838
H	10.144078	0.739825	-2.747663
H	7.825814	3.463060	-0.399312
H	5.019104	1.562524	0.053435
H	6.004424	2.838695	0.778311
H	6.022612	1.204465	1.452513
H	9.654943	-1.451123	-2.931945
H	7.947183	-1.884225	-2.910635
H	8.963571	-2.231190	-1.511504
H	10.767941	3.550868	-1.185842
H	9.499428	4.296534	-2.156517
H	10.608078	3.128066	-2.892403
H	-7.751801	-1.340412	-2.300832
H	-5.125987	-4.337561	-3.842596
H	-2.215297	-3.470422	-2.828510
H	-3.233061	-4.910342	-2.736266
H	-2.684841	-4.108006	-1.259987
H	-5.720062	-0.357438	0.503936
H	-7.284439	-0.306672	-0.310816
H	-5.966126	0.707650	-0.880741
H	-8.613741	-3.012365	-3.876766
H	-7.465075	-2.775031	-5.195300
H	-7.582402	-4.349911	-4.411188
H	-3.552175	-0.068189	-0.215760
H	-4.276802	3.510845	2.062165
H	-5.582974	2.503578	1.462061
H	-3.229396	2.642935	-2.475840

H	-4.891532	2.693708	-1.915659
H	-0.996851	3.312161	0.291633
H	-0.563073	1.672669	-0.168377
H	-1.256157	2.573999	2.695973
H	-0.836574	0.931815	2.242230
H	1.417381	1.713862	1.478989
H	1.000357	3.361371	1.923074
H	2.369476	2.288172	3.727362
H	0.748634	2.643608	4.340663
H	1.179378	0.990251	3.883100
H	-4.400182	4.829082	-0.643275
H	-2.738591	4.781225	-1.201983
H	-3.529575	4.960108	-3.575834
H	-5.192524	5.008902	-3.018115
H	-4.355570	7.326789	-3.484302
H	-4.714766	7.153301	-1.761518
H	-3.039942	7.104243	-2.323873
H	-4.705445	0.564904	2.867388
H	-3.438554	1.601070	3.482838
H	-5.169377	3.136458	4.466706
H	-6.438552	2.092981	3.851384
H	-5.999101	1.596537	6.267984
H	-5.570772	0.190891	5.282399
H	-4.302544	1.249395	5.907815
H	6.823893	-1.931055	-0.995369

Optimized structure of ***C^m^***

Atom	x	y	z
C	3.641106	-4.720390	-1.031141
C	4.177542	-3.413560	-0.945907
C	5.367169	-3.111787	-1.645848
C	5.991303	-4.107273	-2.407783
C	5.478097	-5.400453	-2.500113
C	4.300020	-5.680940	-1.804028
C	3.497329	-2.324577	-0.119718
C	2.081029	-1.919093	-0.510579
C	1.277212	-1.793131	0.639885
C	2.064302	-2.186436	1.814789
C	3.357032	-2.552291	1.380392
C	1.583998	-1.635161	-1.783192
C	0.264132	-1.211567	-1.926289
C	-0.559965	-1.063007	-0.797916
C	-0.044580	-1.359028	0.486155
S	-1.246196	-1.115718	1.758908
C	-2.453822	-0.605159	0.573840
C	-1.947620	-0.625892	-0.746982
C	-3.774080	-0.214424	0.824277
C	-4.579279	0.167611	-0.266812
C	-4.092313	0.153510	-1.574235
C	-2.779549	-0.246825	-1.813982
C	-4.566799	-0.121362	2.056133
C	-5.863905	0.320452	1.716419
C	-5.946731	0.626740	0.225864
C	-6.839517	0.474378	2.692858
C	-6.517228	0.186848	4.024133
C	-5.231301	-0.242336	4.366290
C	-4.246440	-0.399886	3.388286

C	4.312061	-2.992969	2.287734
C	3.974705	-3.068432	3.644272
C	2.697974	-2.699507	4.079802
C	1.733267	-2.255992	3.171731
C	-7.176898	0.136787	-0.534437
C	-8.035662	1.078026	-1.144929
C	-9.166600	0.632404	-1.840172
C	-9.479271	-0.721529	-1.955317
C	-8.622126	-1.638381	-1.342335
C	-7.481627	-1.241354	-0.638169
C	-7.787006	2.572664	-1.077299
C	-6.617000	-2.311203	-0.010915
C	-10.687514	-1.185060	-2.733763
C	6.008060	-1.737719	-1.610131
C	6.181182	-6.468791	-3.303286
C	2.370587	-5.122207	-0.316981
C	1.305718	2.806052	-2.001652
C	0.313242	3.842781	-1.451989
C	-1.018260	3.872690	-2.221814
C	-2.002555	4.916200	-1.683711
Sn	3.213715	2.708503	-0.866962
C	2.700682	2.398555	1.276230
C	3.821667	1.848308	2.166568
C	3.408936	1.698338	3.639212
C	4.525266	1.137397	4.525901
C	4.207388	4.690831	-1.076838
C	5.529580	4.854497	-0.318067
C	6.159305	6.247192	-0.481394
C	7.480822	6.407368	0.276953
H	-2.397494	-0.269157	-2.830111
H	-4.736530	0.439545	-2.400315
H	2.217602	-1.756556	-2.656652
H	-0.131079	-0.997300	-2.914632
H	0.744636	-1.980305	3.524251
H	2.450645	-2.762700	5.135316
H	4.708992	-3.418901	4.363629
H	5.301385	-3.286449	1.947838
H	-7.840427	0.801781	2.425451
H	-7.272025	0.297113	4.797229
H	-4.995083	-0.459983	5.403732
H	-3.254444	-0.738679	3.669076
H	-8.845970	-2.700834	-1.410780
H	-9.820225	1.368705	-2.302465
H	-6.831437	2.857299	-1.532844
H	-8.572839	3.112721	-1.611385
H	-7.778240	2.947943	-0.047456
H	-7.044989	-3.301135	-0.188827
H	-5.603095	-2.305914	-0.422662
H	-6.522815	-2.175702	1.070640
H	-10.409356	-1.506570	-3.745274
H	-11.174004	-2.036966	-2.248106
H	-11.427649	-0.386331	-2.837217
H	6.904853	-3.859876	-2.943765
H	3.874683	-6.680544	-1.863040
H	1.513277	-4.528270	-0.648257
H	2.142031	-6.174133	-0.506719
H	2.453003	-4.987108	0.765692

H	6.299454	-1.440790	-0.595978
H	6.912933	-1.724870	-2.222925
H	5.343824	-0.955471	-1.995837
H	6.847470	-6.032470	-4.053060
H	5.466190	-7.116111	-3.821011
H	6.791555	-7.113638	-2.658684
H	4.101690	-1.415251	-0.225000
H	3.473247	5.431408	-0.731125
H	4.353451	4.872898	-2.147947
H	0.865704	1.803206	-1.978606
H	1.548254	3.022397	-3.048677
H	2.360322	3.374574	1.647434
H	1.831910	1.730709	1.298100
H	4.704832	2.500521	2.111319
H	4.148888	0.868686	1.791710
H	2.530976	1.041882	3.698082
H	3.087302	2.676322	4.022975
H	4.206622	1.061831	5.570999
H	5.415628	1.776527	4.496442
H	4.822479	0.135455	4.197230
H	0.760612	4.846396	-1.481157
H	0.101525	3.637355	-0.393613
H	-1.478069	2.876467	-2.178349
H	-0.814150	4.070736	-3.282945
H	-2.939760	4.913342	-2.250385
H	-1.582242	5.926816	-1.743069
H	-2.249009	4.722937	-0.633480
H	6.252193	4.098393	-0.656032
H	5.375116	4.662029	0.752973
H	5.443712	7.007006	-0.138915
H	6.321250	6.443970	-1.549955
H	7.903529	7.408343	0.141249
H	8.226686	5.682393	-0.068779
H	7.342335	6.248639	1.352595
H	-5.934885	1.720595	0.147669

Optimized structure of ***C'***

Atom	x	y	z
C	8.045789	-0.690339	-1.443754
C	6.944396	-0.378069	-0.615732
C	6.748673	0.962840	-0.207856
C	7.646654	1.945562	-0.634783
C	8.737843	1.654871	-1.456806
C	8.918621	0.328140	-1.845901
C	5.995702	-1.491502	-0.177789
C	4.535436	-1.385062	-0.602326
C	3.691608	-1.706656	0.479038
C	4.515228	-1.947465	1.669914
C	5.870379	-1.773525	1.314484
C	4.022525	-1.082877	-1.864186
C	2.644457	-1.101781	-2.068117
C	1.775818	-1.428526	-1.013176
C	2.309287	-1.729450	0.262035
C	0.322031	-1.502411	-1.033026
C	-0.213387	-1.853104	0.228888
S	1.047186	-2.099451	1.442519
C	-1.596388	-1.976326	0.406080

C	-2.439304	-1.734314	-0.696854
C	-1.924432	-1.390180	-1.947360
C	-0.545486	-1.278666	-2.115490
C	-2.421715	-2.340238	1.564104
C	-3.776563	-2.319319	1.167180
C	-3.900004	-1.845388	-0.275790
C	-4.783977	-2.649558	2.064756
C	-4.436619	-3.002890	3.374025
C	-3.096529	-3.016848	3.773627
C	-2.079051	-2.686489	2.875003
C	6.876344	-1.917836	2.261184
C	6.527080	-2.241204	3.577350
C	5.186825	-2.422694	3.931808
C	4.170932	-2.278337	2.983991
C	-4.849267	-2.607956	-1.197106
C	-4.647649	-3.976017	-1.497920
C	-5.544405	-4.628353	-2.349107
C	-6.642188	-3.978596	-2.918058
C	-6.824439	-2.630144	-2.613575
C	-5.953484	-1.934519	-1.765875
C	-3.488786	-4.766664	-0.934373
C	-6.242697	-0.470327	-1.496540
C	-7.609047	-4.719630	-3.810687
C	5.599297	1.378958	0.681654
C	9.672573	2.744739	-1.924965
C	8.332834	-2.100988	-1.921284
C	-0.690118	2.842870	-1.747908
Sn	-2.523328	3.040666	-0.508423
C	-3.013850	5.210137	-0.394753
C	-4.230772	5.571099	0.465226
C	-4.510529	7.081410	0.523845
C	-5.727564	7.439577	1.382735
C	0.528534	3.579310	-1.169759
C	1.802171	3.395205	-2.012585
C	3.013043	4.145018	-1.448023
C	-2.028173	2.318254	1.537737
C	-3.219220	1.939445	2.426749
C	-2.805543	1.488584	3.836427
C	-3.994763	1.096334	4.719051
H	2.238849	-0.864464	-3.046979
H	4.692621	-0.824047	-2.678579
H	-2.593189	-1.224630	-2.786673
H	-0.138367	-1.021235	-3.088585
H	-1.043370	-2.709103	3.198274
H	-2.842022	-3.292262	4.792776
H	-5.213426	-3.270843	4.084130
H	-5.824327	-2.645813	1.751576
H	7.917231	-1.770463	1.986650
H	7.302994	-2.351100	4.329306
H	4.930838	-2.675066	4.956616
H	3.135028	-2.417805	3.275756
H	9.763900	0.072236	-2.480812
H	7.485620	2.971552	-0.310830
H	4.630776	1.160143	0.221610
H	5.640199	2.453001	0.880532
H	5.620572	0.858371	1.643788
H	9.230643	-2.115426	-2.544329

H	7.514220	-2.512065	-2.523396
H	8.503401	-2.795835	-1.090856
H	9.257970	3.280281	-2.788228
H	10.641710	2.338095	-2.228196
H	9.847195	3.487020	-1.139668
H	-7.668501	-2.098158	-3.046717
H	-5.375491	-5.679317	-2.573947
H	-2.525024	-4.353575	-1.247578
H	-3.533743	-5.804797	-1.273564
H	-3.493637	-4.770958	0.159738
H	-6.421070	-0.270277	-0.433629
H	-7.137097	-0.153992	-2.039183
H	-5.422355	0.184354	-1.813459
H	-8.171524	-4.032355	-4.449254
H	-7.089884	-5.434547	-4.456907
H	-8.338215	-5.288963	-3.220699
H	-4.267599	-0.813628	-0.217626
H	-2.111278	5.701407	-0.006275
H	-3.147923	5.570599	-1.421333
H	-0.482455	1.771564	-1.843484
H	-0.921407	3.212508	-2.753697
H	-1.440588	3.119681	2.005412
H	-1.354998	1.462145	1.414760
H	-3.911725	2.788556	2.515840
H	-3.795483	1.131715	1.954457
H	-2.117716	0.637263	3.751092
H	-2.236881	2.295678	4.318489
H	-3.668551	0.800065	5.721652
H	-4.698214	1.929681	4.832211
H	-4.543608	0.252314	4.287005
H	0.314014	4.653885	-1.083793
H	0.730860	3.229306	-0.148058
H	2.032507	2.323676	-2.081810
H	1.604580	3.733259	-3.038999
H	3.903822	3.991246	-2.066015
H	2.824363	5.223712	-1.398135
H	3.251010	3.804495	-0.433853
H	-5.125638	5.060704	0.082121
H	-4.090741	5.199239	1.489962
H	-3.621291	7.596278	0.912230
H	-4.656385	7.458418	-0.497600
H	-5.900361	8.520652	1.404187
H	-6.637784	6.965496	0.997798
H	-5.594909	7.104123	2.417748
H	6.364516	-2.422677	-0.624658

Optimized structure of $\boldsymbol{D}^{CS}$

Atom	x	y	z
C	-0.700214	-1.220377	0.002690
C	-1.101130	0.162253	0.003380
C	1.100726	0.161707	-0.003627
C	0.699134	-1.220718	-0.001428
C	-1.699772	-2.242353	0.002426
C	-3.023411	-1.886145	0.003466
C	-3.410102	-0.506445	0.010509
C	-2.406305	0.550450	0.006194
C	2.406093	0.549261	-0.006899

C	3.409390	-0.508107	-0.010218
C	3.022017	-1.887615	-0.001454
C	1.698201	-2.243172	0.000052
C	3.109649	1.824962	-0.007852
C	4.503969	1.520887	-0.008205
C	4.677082	0.072664	-0.010049
C	-4.677512	0.074909	0.009542
C	-4.503747	1.523046	0.006640
C	-3.109282	1.826475	0.005997
C	2.671292	3.144437	-0.014367
C	3.626099	4.170252	-0.021907
C	4.995245	3.877292	-0.026709
C	5.445494	2.553624	-0.022562
C	-5.444809	2.556226	0.020248
C	-4.993936	3.879688	0.023373
C	-3.624656	4.172009	0.018228
C	-2.670317	3.145750	0.011430
C	-5.982102	-0.632323	0.018529
C	-6.736784	-0.739362	-1.171512
C	-7.967626	-1.401157	-1.139392
C	-8.477010	-1.960181	0.035871
C	-7.719002	-1.837459	1.203155
C	-6.481333	-1.186712	1.218239
C	-5.711315	-1.069289	2.514643
C	-6.223197	-0.167787	-2.473586
C	-9.795703	-2.696680	0.038334
C	5.981686	-0.634494	-0.018311
C	6.735055	-0.742174	1.172599
C	7.962841	-1.409641	1.142993
C	8.472897	-1.969379	-0.031691
C	7.712743	-1.852662	-1.198123
C	6.477997	-1.196353	-1.215704
C	5.704736	-1.087915	-2.510978
C	6.219295	-0.172288	2.474556
C	9.818768	-2.654844	-0.045266
H	-1.403509	-3.286097	-0.003030
H	-3.798284	-2.646886	-0.004184
H	3.796482	-2.648764	0.007274
H	1.401448	-3.286769	0.006846
H	1.610764	3.375575	-0.015392
H	3.298942	5.205722	-0.026409
H	5.716653	4.689109	-0.036164
H	6.508122	2.328487	-0.033420
H	-6.507549	2.331615	0.031377
H	-5.714972	4.691839	0.032275
H	-3.297022	5.207331	0.021902
H	-1.609683	3.376405	0.012227
H	-8.542345	-1.482697	-2.059504
H	-8.102790	-2.253804	2.132071
H	-4.782296	-1.649082	2.489381
H	-6.308915	-1.431876	3.355214
H	-5.428237	-0.031808	2.720125
H	-6.910256	-0.389242	-3.294490
H	-5.241982	-0.580910	-2.730828
H	-6.100912	0.919080	-2.418868
H	-9.656457	-3.760759	-0.190858
H	-10.482226	-2.291666	-0.711260

H	-10.285776	-2.637180	1.014730
H	8.533232	-1.497722	2.065195
H	8.091151	-2.280742	-2.123907
H	4.776542	-1.668780	-2.480617
H	6.301104	-1.454289	-3.350781
H	5.419861	-0.051977	-2.721627
H	6.096704	0.914572	2.420370
H	6.905503	-0.393920	3.296124
H	5.237902	-0.585901	2.730350
H	10.036486	-3.128928	0.916585
H	9.868904	-3.423928	-0.821808
H	10.626540	-1.939235	-0.244130
O	0.000001	0.990210	-0.000536

Optimized structure of $\boldsymbol{D}^{OSS}$

Atom	x	y	z
C	-0.711607	-1.236898	0.003164
C	-1.101068	0.136863	0.004580
C	1.100655	0.136329	-0.004892
C	0.710539	-1.237239	-0.002388
C	-1.704879	-2.248242	0.003825
C	-3.037780	-1.886532	0.006494
C	-3.411070	-0.514607	0.013641
C	-2.413788	0.531324	0.008905
C	2.413566	0.530157	-0.009570
C	3.410361	-0.516239	-0.013623
C	3.036409	-1.887986	-0.005244
C	1.703333	-2.249050	-0.002195
C	3.109295	1.812310	-0.009115
C	4.507798	1.521227	-0.009115
C	4.693664	0.081148	-0.012640
C	-4.694093	0.083358	0.012041
C	-4.507580	1.523354	0.007915
C	-3.108941	1.813792	0.007706
C	2.659593	3.126966	-0.013847
C	3.605527	4.162148	-0.019164
C	4.977828	3.883098	-0.023363
C	5.440266	2.564330	-0.020938
C	-5.439572	2.566899	0.019398
C	-4.976509	3.885451	0.021232
C	-3.604080	4.163862	0.016742
C	-2.658625	3.128239	0.011799
C	-5.998882	-0.621670	0.019037
C	-6.748406	-0.734951	-1.173458
C	-7.979014	-1.397323	-1.142865
C	-8.492269	-1.951445	0.033021
C	-7.738636	-1.823274	1.202689
C	-6.501685	-1.171564	1.219309
C	-5.735262	-1.046013	2.516914
C	-6.230119	-0.167900	-2.475732
C	-9.810262	-2.689197	0.033814
C	5.998489	-0.623790	-0.019003
C	6.746031	-0.739081	1.174623
C	7.973579	-1.407121	1.146411
C	8.488133	-1.960615	-0.029257
C	7.732990	-1.837086	-1.198384
C	6.499001	-1.179761	-1.217375

C	5.729931	-1.061725	-2.514129
C	6.224890	-0.175075	2.477079
C	9.833388	-2.647315	-0.040916
H	-1.415251	-3.294079	-0.001385
H	-3.813494	-2.646255	-0.000088
H	3.811728	-2.648110	0.002100
H	1.413223	-3.294749	0.003977
H	1.597103	3.348682	-0.015015
H	3.268221	5.194396	-0.022297
H	5.691017	4.702098	-0.030924
H	6.504865	2.348686	-0.031127
H	-6.504279	2.351790	0.029851
H	-5.689322	4.704779	0.028551
H	-3.266295	5.195955	0.019388
H	-1.596033	3.349463	0.012804
H	-8.550233	-1.483507	-2.064722
H	-8.125200	-2.236355	2.131899
H	-4.791568	-1.601127	2.485980
H	-6.323189	-1.430341	3.354587
H	-5.479203	-0.003103	2.731279
H	-6.909971	-0.399524	-3.299817
H	-5.244341	-0.575632	-2.724076
H	-6.116346	0.920225	-2.427244
H	-9.668955	-3.754317	-0.189154
H	-10.493988	-2.288813	-0.720798
H	-10.304500	-2.625048	1.007811
H	8.539965	-1.500899	2.070507
H	8.114642	-2.260865	-2.124804
H	4.786841	-1.617615	-2.479114
H	6.316904	-1.449233	-3.351004
H	5.472489	-0.020077	-2.732884
H	6.110633	0.913067	2.430104
H	6.903568	-0.407540	3.301896
H	5.238908	-0.583704	2.723154
H	10.047093	-3.125904	0.919591
H	9.885647	-3.412953	-0.820712
H	10.642537	-1.931581	-0.233625
O	-0.000006	0.965906	-0.000429

Optimized structure of $\boldsymbol{D}^{T}$

Atom	x	y	z
C	-0.725416	-1.253318	0.004509
C	-1.101924	0.110814	0.007570
C	1.101427	0.110259	-0.008260
C	0.724238	-1.253682	-0.004683
C	-1.711768	-2.252932	0.006281
C	-3.055556	-1.884646	0.012373
C	-3.412885	-0.521838	0.022345
C	-2.423306	0.512492	0.015577
C	2.423012	0.511270	-0.016371
C	3.412086	-0.523552	-0.022802
C	3.054071	-1.886185	-0.012237
C	1.710094	-2.253786	-0.005981
C	3.111255	1.797948	-0.013702
C	4.516414	1.520570	-0.013130
C	4.717950	0.095133	-0.020182
C	-4.718454	0.097464	0.019449

C	-4.516233	1.522813	0.012440
C	-3.110936	1.799498	0.012772
C	2.649880	3.107843	-0.016949
C	3.585560	4.153048	-0.020459
C	4.962784	3.889792	-0.025055
C	5.438733	2.578307	-0.024422
C	-5.438012	2.581030	0.024126
C	-4.961392	3.892272	0.024740
C	-3.584038	4.154837	0.019738
C	-2.648893	3.109159	0.015956
C	-6.021228	-0.607149	0.023384
C	-6.784893	-0.692673	-1.162881
C	-8.012634	-1.360417	-1.134464
C	-8.509644	-1.947274	0.032437
C	-7.742085	-1.847301	1.195903
C	-6.507503	-1.191741	1.214726
C	-5.728369	-1.091704	2.506823
C	-6.284229	-0.093480	-2.457835
C	-9.824569	-2.690358	0.029563
C	6.020806	-0.609347	-0.023537
C	6.781401	-0.698787	1.164478
C	8.006194	-1.371936	1.138601
C	8.505658	-1.956056	-0.028687
C	7.737632	-1.858784	-1.192011
C	6.505933	-1.197807	-1.213360
C	5.725200	-1.103176	-2.504901
C	6.276628	-0.104646	2.460160
C	9.848051	-2.648230	-0.038373
H	-1.429884	-3.301169	-0.000411
H	-3.832121	-2.643280	0.005379
H	3.830227	-2.645237	-0.004793
H	1.427701	-3.301882	0.001218
H	1.585338	3.319282	-0.018507
H	3.237046	5.181604	-0.022400
H	5.666033	4.717255	-0.031983
H	6.505475	2.373650	-0.036265
H	-6.504864	2.376960	0.036401
H	-5.664229	4.720081	0.031984
H	-3.235006	5.183218	0.021641
H	-1.584242	3.320053	0.017330
H	-8.594076	-1.425920	-2.051613
H	-8.115896	-2.285906	2.118608
H	-4.769277	-1.616683	2.444296
H	-6.294139	-1.522686	3.336970
H	-5.500462	-0.049811	2.756016
H	-6.960928	-0.329728	-3.283217
H	-5.289741	-0.473497	-2.715119
H	-6.197113	0.996296	-2.394631
H	-9.681441	-3.748977	-0.221440
H	-10.518334	-2.274250	-0.707151
H	-10.308010	-2.652626	1.010337
H	8.581976	-1.446430	2.058628
H	8.107431	-2.306347	-2.112028
H	4.766618	-1.628784	-2.439929
H	6.290660	-1.536117	-3.334240
H	5.496174	-0.062261	-2.757104
H	6.188510	0.985220	2.399881

H	6.951691	-0.342478	3.286427
H	5.281931	-0.486458	2.713991
H	10.071560	-3.102258	0.931764
H	9.888064	-3.434243	-0.798367
H	10.657412	-1.941006	-0.259537
O	-0.000039	0.940244	-0.000521

Optimized structure of ***TS3***

Atom	x	y	z
C	-8.308984	-0.799058	-1.410566
C	-7.959403	0.102852	-0.380536
C	-8.897275	1.065269	0.056968
C	-10.159640	1.106920	-0.542110
C	-10.525945	0.221042	-1.559031
C	-9.584045	-0.721815	-1.979008
C	-6.614186	0.040898	0.239136
C	-5.396518	0.528300	-0.324296
C	-4.327060	0.267759	0.602682
C	-4.893281	-0.401031	1.768706
C	-6.297496	-0.530482	1.530435
C	-5.157688	1.184365	-1.556532
C	-3.874294	1.581942	-1.899522
C	-2.811471	1.330259	-1.006203
C	-3.067584	0.678949	0.230075
C	-1.397884	1.590487	-1.019842
C	-0.902926	1.079000	0.203649
O	-1.910454	0.529023	0.966385
C	0.431355	1.161370	0.555649
C	1.314372	1.774037	-0.382836
C	0.837157	2.303844	-1.597972
C	-0.511688	2.215471	-1.919924
C	1.191687	0.794141	1.745087
C	2.537262	1.182192	1.527918
C	2.685555	1.709898	0.140687
C	3.501580	0.936779	2.504621
C	3.111263	0.340043	3.707921
C	1.777604	-0.030335	3.925420
C	0.808158	0.188087	2.943062
C	-7.114202	-1.153611	2.482796
C	-6.535496	-1.631310	3.660698
C	-5.159743	-1.497446	3.889796
C	-4.327915	-0.882386	2.943153
C	3.717945	2.743671	-0.234146
C	4.468804	2.676142	-1.433080
C	5.431664	3.657707	-1.703030
C	5.672454	4.727115	-0.843985
C	4.869153	4.826606	0.293255
C	3.893644	3.876989	0.608219
C	4.291661	1.601758	-2.487767
C	3.018700	4.154981	1.813351
C	6.745550	5.747576	-1.136616
C	-8.544086	2.060827	1.138784
C	-11.908111	0.263773	-2.166705
C	-7.334213	-1.850675	-1.890860
Sn	3.621549	-1.501991	-0.720264
C	2.819046	-2.794782	0.880477
C	3.832267	-3.211583	1.955380

C	3.222412	-4.114643	3.039130
C	4.232308	-4.542147	4.108817
C	2.730784	-2.109219	-2.647704
C	3.028041	-3.565340	-3.039878
C	2.397165	-3.974375	-4.381012
C	2.692443	-5.426094	-4.771027
C	5.818253	-1.735347	-0.862667
C	6.640174	-0.871593	0.105110
C	8.156875	-1.071887	-0.038049
C	8.974821	-0.209175	0.928201
H	-3.686597	2.083929	-2.843407
H	-5.989370	1.375977	-2.227764
H	1.523821	2.800223	-2.275727
H	-0.881957	2.631623	-2.851551
H	-0.224991	-0.100353	3.107305
H	1.494663	-0.491684	4.866929
H	3.850708	0.163060	4.483616
H	4.537788	1.218295	2.338461
H	-8.179709	-1.264469	2.302523
H	-7.158336	-2.114809	4.407582
H	-4.730383	-1.877242	4.812210
H	-3.262014	-0.786000	3.124017
H	-10.873480	1.856689	-0.207608
H	-9.848085	-1.421094	-2.769503
H	-6.458664	-1.402865	-2.372930
H	-7.809120	-2.520963	-2.612214
H	-6.959001	-2.456550	-1.059352
H	-9.359214	2.772374	1.294894
H	-7.642978	2.627459	0.880871
H	-8.340431	1.565780	2.094162
H	-12.320131	1.277359	-2.153721
H	-12.605523	-0.376881	-1.612284
H	-11.901094	-0.086441	-3.203259
H	6.001430	3.582114	-2.626811
H	4.985556	5.685863	0.950312
H	1.973510	3.895527	1.626723
H	3.060989	5.218810	2.063082
H	3.332005	3.593922	2.698873
H	4.972142	0.758476	-2.324965
H	4.528559	2.011901	-3.474362
H	3.276307	1.206965	-2.529418
H	6.979942	5.787984	-2.204381
H	6.443391	6.749757	-0.816826
H	7.675933	5.506666	-0.607145
H	3.177674	0.388870	-0.389912
H	6.037152	-2.798565	-0.700354
H	6.102006	-1.519130	-1.900101
H	1.648854	-1.945232	-2.582872
H	3.102295	-1.425134	-3.419752
H	2.401178	-3.680623	0.384714
H	1.977585	-2.265544	1.338820
H	4.681828	-3.735458	1.494961
H	4.250211	-2.318605	2.438571
H	2.385580	-3.584151	3.512383
H	2.789285	-5.005729	2.564523
H	3.768150	-5.181320	4.867195
H	5.065042	-5.102090	3.667727

H	4.657073	-3.672141	4.622711
H	4.113926	-3.726298	-3.097768
H	2.664918	-4.248277	-2.258786
H	1.311171	-3.820124	-4.326772
H	2.760565	-3.299563	-5.167796
H	2.230288	-5.685720	-5.729100
H	3.770495	-5.600614	-4.864427
H	2.309960	-6.124674	-4.018077
H	6.406633	0.190903	-0.050067
H	6.351027	-1.092960	1.141824
H	8.396093	-2.132080	0.121591
H	8.452056	-0.847348	-1.072158
H	10.050010	-0.372349	0.800599
H	8.779093	0.857439	0.769503
H	8.726188	-0.437796	1.970947

Optimized structure of ***E***

Atom	x	y	z
O	0.054473	0.907876	0.273264
C	-1.060097	0.095412	0.269355
C	-0.710628	-1.264226	0.457818
C	0.731394	-1.276095	0.588769
C	1.132277	0.070183	0.467662
C	-2.366947	0.506926	0.105550
C	-3.374863	-0.512855	0.134479
C	-3.041555	-1.873169	0.314435
C	-1.713108	-2.250273	0.478554
C	1.699042	-2.273073	0.786771
C	3.044509	-1.909051	0.867642
C	3.414462	-0.566700	0.744551
C	2.464142	0.456027	0.536338
C	-3.026546	1.791256	-0.107245
C	-4.429203	1.526891	-0.208808
C	-4.655821	0.108681	-0.060267
C	4.806359	0.048414	0.867394
C	4.534600	1.518257	0.559297
C	3.148938	1.744028	0.419961
C	5.432183	2.574692	0.466151
C	4.938609	3.863908	0.233889
C	3.563914	4.088485	0.103390
C	2.655850	3.031474	0.195255
C	-2.543221	3.089504	-0.207863
C	-3.455687	4.135586	-0.410243
C	-4.830990	3.884611	-0.505966
C	-5.328689	2.584099	-0.403768
C	5.952029	-0.633134	0.122831
C	7.045386	-1.149263	0.853686
C	8.097530	-1.774891	0.173551
C	8.107400	-1.906889	-1.214472
C	7.017140	-1.394347	-1.921381
C	5.944099	-0.762195	-1.286466
C	-5.968400	-0.577862	-0.098018
C	-6.615083	-0.803489	-1.334041
C	-7.854925	-1.449290	-1.345049
C	-8.475975	-1.879584	-0.169482
C	-7.822716	-1.642407	1.042953
C	-6.581146	-1.002662	1.102470

C	-5.977482	-0.375511	-2.636616
C	-9.801242	-2.603031	-0.209990
C	-5.927549	-0.751327	2.442717
C	7.131877	-1.057687	2.365115
C	9.264843	-2.558390	-1.933226
C	4.809466	-0.243407	-2.140236
H	-1.451245	-3.294393	0.618270
H	-3.828873	-2.620753	0.319877
H	3.805998	-2.669422	1.013111
H	1.405527	-3.314521	0.874722
H	1.589660	3.202081	0.089028
H	3.198920	5.095700	-0.075397
H	5.629961	4.697665	0.153002
H	6.500761	2.401935	0.560394
H	-6.395492	2.390271	-0.470022
H	-5.517204	4.712346	-0.658781
H	-3.090130	5.155015	-0.491134
H	-1.479773	3.291814	-0.129407
H	-8.346290	-1.622782	-2.300036
H	-8.294015	-1.957971	1.971308
H	-4.981238	-1.293828	2.540653
H	-6.581832	-1.065941	3.260016
H	-5.696747	0.310233	2.581804
H	-6.580271	-0.697722	-3.489757
H	-4.974971	-0.802133	-2.748962
H	-5.864031	0.712128	-2.695084
H	-9.658772	-3.687968	-0.292425
H	-10.402792	-2.289420	-1.068412
H	-10.384549	-2.421430	0.697745
H	8.931361	-2.169763	0.749646
H	6.996293	-1.488820	-3.005007
H	3.860333	-0.727609	-1.890702
H	5.011729	-0.428909	-3.198292
H	4.662625	0.832966	-2.010189
H	7.128643	-0.020795	2.720601
H	8.056925	-1.518276	2.720948
H	6.301533	-1.572276	2.862457
H	9.839809	-3.204093	-1.263187
H	8.921593	-3.165571	-2.776919
H	9.955619	-1.807461	-2.336740
H	5.050078	0.010967	1.936065

Optimized structure of ***TS4***^{m}

Atom	x	y	z
C	-3.692188	-4.127679	-0.105255
C	-4.087545	-2.774343	0.088525
C	-5.466117	-2.460216	0.004488
C	-6.382206	-3.455004	-0.360465
C	-5.996746	-4.766194	-0.630010
C	-4.646467	-5.081006	-0.470716
C	-3.039542	-1.758395	0.459651
C	-1.727160	-1.667775	-0.227130
C	-0.768262	-1.161624	0.687864
C	-1.392749	-1.022275	1.998688
C	-2.747291	-1.435111	1.874079
C	-1.410915	-1.885606	-1.577573
C	-0.120315	-1.636888	-2.043818

C	0.849548	-1.146971	-1.153703
C	0.504530	-0.914814	0.192618
O	1.572625	-0.424328	0.914445
C	2.623207	-0.342370	0.023858
C	2.247875	-0.771760	-1.264400
C	3.908720	0.095558	0.315255
C	4.836266	0.102455	-0.747960
C	4.490737	-0.319169	-2.034616
C	3.193138	-0.762943	-2.300266
C	4.562715	0.561379	1.538591
C	5.906152	0.862848	1.230317
C	6.163029	0.682425	-0.263237
C	6.772021	1.308867	2.221072
C	6.289221	1.455198	3.526965
C	4.955367	1.162999	3.830950
C	4.079370	0.713204	2.840497
C	-3.571792	-1.469874	3.001124
C	-3.051568	-1.069708	4.234844
C	-1.722546	-0.640659	4.348394
C	-0.881457	-0.618602	3.232728
C	7.445653	-0.032686	-0.681424
C	8.402848	0.656444	-1.459111
C	9.579897	0.003142	-1.844968
C	9.846464	-1.316984	-1.484122
C	8.888104	-1.984916	-0.718263
C	7.698707	-1.374611	-0.309667
C	8.209583	2.093231	-1.904792
C	6.723589	-2.183494	0.515147
C	11.134472	-1.996139	-1.884809
C	-6.040004	-1.089053	0.295998
C	-6.998700	-5.808664	-1.063476
C	-2.278873	-4.617536	0.135396
Sn	-3.603124	1.446571	-0.738252
C	-4.799297	2.699718	0.635565
C	-4.383643	2.657408	2.113291
C	-5.271022	3.525740	3.018649
C	-4.849323	3.488360	4.491056
C	-1.597738	2.307976	-1.063484
C	-0.842419	2.784942	0.184410
C	0.543119	3.367075	-0.137878
C	1.318585	3.808734	1.107137
C	-4.622074	1.408364	-2.700063
C	-4.819742	2.797906	-3.326868
C	-5.514125	2.754628	-4.697813
C	-5.709756	4.140094	-5.321604
H	2.921094	-1.098099	-3.296415
H	5.235681	-0.313470	-2.824764
H	-2.172204	-2.260796	-2.255184
H	0.131038	-1.818587	-3.084285
H	0.154516	-0.308862	3.324370
H	-1.338474	-0.333153	5.316544
H	-3.683283	-1.098625	5.117893
H	-4.596521	-1.820485	2.924551
H	7.810366	1.529489	1.989134
H	6.957347	1.797490	4.311892
H	4.597928	1.282939	4.849536
H	3.046649	0.477872	3.075770

H	9.067868	-3.017758	-0.427451
H	10.306398	0.545601	-2.445844
H	7.317699	2.219814	-2.529379
H	9.067217	2.426265	-2.494756
H	8.111399	2.782489	-1.058142
H	7.120000	-3.184891	0.701994
H	5.759190	-2.293458	0.009805
H	6.524796	-1.714985	1.483679
H	11.861800	-1.985601	-1.063372
H	11.600511	-1.498019	-2.739886
H	10.967797	-3.044330	-2.152961
H	-7.435160	-3.189139	-0.425357
H	-4.322370	-6.109987	-0.611267
H	-1.612331	-4.406568	-0.706267
H	-2.283714	-5.699938	0.291287
H	-1.832163	-4.153538	1.018599
H	-5.498932	-0.558454	1.080260
H	-7.082154	-1.183788	0.615422
H	-6.038036	-0.452962	-0.596280
H	-6.743708	-6.797601	-0.670007
H	-8.008813	-5.558761	-0.725752
H	-7.032625	-5.894934	-2.156869
H	-3.488516	-0.377174	-0.047702
H	-4.745053	3.728277	0.255317
H	-5.845134	2.387428	0.525600
H	-4.030022	0.771595	-3.368140
H	-5.590785	0.909210	-2.578010
H	-1.737352	3.142628	-1.763735
H	-1.010661	1.551765	-1.596457
H	-1.430767	3.547637	0.713498
H	-0.720834	1.953451	0.890772
H	1.127953	2.616525	-0.685935
H	0.424510	4.218939	-0.821788
H	2.289525	4.240275	0.842765
H	0.762743	4.563855	1.675257
H	1.505966	2.961170	1.775577
H	-5.410328	3.434599	-2.653030
H	-3.849332	3.301466	-3.440973
H	-4.925140	2.123770	-5.377164
H	-6.487736	2.257606	-4.590261
H	-6.205951	4.075195	-6.295534
H	-6.323280	4.781574	-4.678632
H	-4.749472	4.646751	-5.471477
H	-4.404494	1.623538	2.483383
H	-3.341106	2.986103	2.219467
H	-5.251737	4.562647	2.655988
H	-6.313777	3.192277	2.925316
H	-5.505848	4.105420	5.113462
H	-4.879345	2.466151	4.885059
H	-3.825651	3.858333	4.618901
H	6.210018	1.696140	-0.678995

Optimized structure of ***TS4^r***

Atom	x	y	z
C	-4.391848	-3.303626	-1.840546
C	-4.520666	-2.128148	-1.048596
C	-5.805008	-1.545379	-0.918191

C	-6.882131	-2.073769	-1.641143
C	-6.751250	-3.176655	-2.481890
C	-5.498093	-3.787826	-2.543963
C	-3.310149	-1.609550	-0.317639
C	-1.976745	-1.484803	-0.956924
C	-0.977780	-1.638600	0.038708
C	-1.619689	-1.974170	1.304823
C	-3.022472	-2.011108	1.077563
C	-1.650609	-1.139479	-2.278095
C	-0.316788	-0.973088	-2.649813
C	0.690889	-1.133484	-1.684503
C	0.339497	-1.456839	-0.358757
O	1.450994	-1.562865	0.450931
C	2.535832	-1.312295	-0.363900
C	2.139999	-1.041505	-1.688584
C	3.868445	-1.310737	0.027741
C	4.826183	-1.032461	-0.970270
C	4.460944	-0.761253	-2.291532
C	3.113086	-0.760224	-2.658444
C	4.543569	-1.525801	1.307971
C	5.931753	-1.380439	1.101714
C	6.228510	-1.148101	-0.377312
C	6.817859	-1.508603	2.164096
C	6.310918	-1.785981	3.439219
C	4.935236	-1.938928	3.641843
C	4.038391	-1.811094	2.578812
C	-3.886678	-2.377182	2.112163
C	-3.352569	-2.677230	3.368151
C	-1.971027	-2.616875	3.593740
C	-1.093574	-2.270327	2.562906
C	7.244214	-0.065637	-0.734917
C	7.023140	1.293023	-0.406417
C	7.984610	2.246275	-0.754335
C	9.166299	1.908404	-1.418029
C	9.366753	0.566153	-1.737817
C	8.432727	-0.424181	-1.409394
C	5.774639	1.760304	0.306729
C	8.747028	-1.854829	-1.802778
C	10.198541	2.958426	-1.753767
C	-6.109752	-0.362358	-0.022642
C	-7.918732	-3.699425	-3.283292
C	-3.115349	-4.116729	-1.910183
Sn	-3.178304	1.835502	0.046806
C	-4.190210	2.570161	1.870360
C	-3.869333	1.811590	3.166467
C	-4.632415	2.347214	4.387931
C	-4.304525	1.594661	5.681508
C	-1.029217	2.335607	0.065138
C	-0.269037	2.055380	1.368456
C	1.218764	2.432949	1.289595
C	1.990340	2.125780	2.576731
C	-4.073985	2.856017	-1.697644
C	-3.962361	4.388326	-1.650344
C	-4.578635	5.081909	-2.876202
C	-4.466187	6.608978	-2.828374
H	2.823371	-0.543509	-3.682042
H	5.225346	-0.538387	-3.030004

H	-2.440685	-1.015795	-3.012911
H	-0.060685	-0.721708	-3.674503
H	-0.021679	-2.246967	2.730535
H	-1.576787	-2.853744	4.577538
H	-4.016108	-2.968050	4.177355
H	-4.956323	-2.446357	1.939186
H	7.886300	-1.384271	2.010507
H	6.992461	-1.882497	4.279366
H	4.559881	-2.156050	4.637650
H	2.970844	-1.927413	2.733915
H	10.275922	0.275392	-2.259337
H	7.799991	3.287233	-0.497503
H	4.874009	1.562897	-0.282931
H	5.822515	2.836044	0.494885
H	5.643199	1.256115	1.268786
H	9.713170	-1.905132	-2.311256
H	7.998991	-2.273299	-2.486110
H	8.801995	-2.523073	-0.935654
H	9.728663	3.894050	-2.073385
H	10.864591	2.623435	-2.554174
H	10.824517	3.193124	-0.883728
H	-7.857960	-1.605631	-1.530588
H	-5.377414	-4.689081	-3.141262
H	-2.383368	-3.690816	-2.603012
H	-3.341049	-5.131724	-2.248962
H	-2.625601	-4.189631	-0.935661
H	-5.508012	-0.349833	0.886754
H	-7.162564	-0.383995	0.274383
H	-5.945924	0.588919	-0.541414
H	-8.873793	-3.430932	-2.822207
H	-7.919226	-3.284654	-4.299199
H	-7.882490	-4.788990	-3.380157
H	-3.454991	-0.088584	-0.139180
H	-3.926047	3.630597	1.975783
H	-5.268474	2.541755	1.670514
H	-3.581443	2.467219	-2.596822
H	-5.126629	2.557343	-1.770099
H	-0.963974	3.402072	-0.189560
H	-0.570167	1.789991	-0.766832
H	-0.728762	2.607684	2.199938
H	-0.350809	0.992341	1.629992
H	1.679916	1.896552	0.449615
H	1.305790	3.502256	1.052054
H	3.039874	2.427475	2.496672
H	1.557537	2.652221	3.435510
H	1.972170	1.053462	2.800496
H	-4.451548	4.773948	-0.744676
H	-2.907480	4.687148	-1.571554
H	-4.089608	4.704054	-3.784208
H	-5.634984	4.792093	-2.957824
H	-4.913648	7.072180	-3.713914
H	-4.975122	7.016831	-1.947485
H	-3.418768	6.928189	-2.780301
H	-4.096660	0.743660	3.047025
H	-2.791826	1.860894	3.373156
H	-4.406276	3.414791	4.515182
H	-5.711717	2.288331	4.190770

H	-4.870683	1.989887	6.531467
H	-4.540997	0.528510	5.589495
H	-3.239110	1.673653	5.926017
H	6.627487	-2.099011	-0.750563

Optimized structure of ***F^m***

Atom	x	y	z
C	3.520331	-4.746270	-0.980397
C	4.078722	-3.455215	-0.825489
C	5.359470	-3.191664	-1.360742
C	6.050245	-4.208689	-2.031186
C	5.516266	-5.486987	-2.189614
C	4.247975	-5.729554	-1.657077
C	3.327448	-2.343902	-0.096509
C	1.982247	-1.901861	-0.667464
C	1.051222	-1.750485	0.381898
C	1.667904	-2.156991	1.645227
C	2.993238	-2.560261	1.376577
C	1.656373	-1.610489	-1.994910
C	0.376086	-1.151435	-2.315774
C	-0.565483	-0.980838	-1.290746
C	-0.212047	-1.283808	0.038341
O	-1.259092	-1.061962	0.907648
C	-2.303063	-0.605005	0.130391
C	-1.946201	-0.531079	-1.229565
C	-3.567869	-0.253263	0.585720
C	-4.490783	0.196596	-0.381713
C	-4.161865	0.280514	-1.737243
C	-2.885814	-0.087392	-2.171098
C	-4.202920	-0.256756	1.904069
C	-5.530554	0.196218	1.752464
C	-5.791056	0.606450	0.305846
C	-6.378214	0.270406	2.850799
C	-5.892592	-0.109033	4.107704
C	-4.573661	-0.550293	4.259114
C	-3.716224	-0.628328	3.159592
C	3.809351	-3.022381	2.401953
C	3.295664	-3.079357	3.703232
C	1.983827	-2.672139	3.970532
C	1.157449	-2.207119	2.944907
C	-7.104026	0.160138	-0.333104
C	-8.031945	1.133812	-0.765191
C	-9.239236	0.727670	-1.347199
C	-9.562498	-0.617533	-1.519830
C	-8.635637	-1.567256	-1.083664
C	-7.417898	-1.210093	-0.497583
C	-7.778361	2.622246	-0.621745
C	-6.481727	-2.313139	-0.058974
C	-10.857056	-1.036804	-2.174864
C	6.029731	-1.836394	-1.242000
C	6.289046	-6.579511	-2.889586
C	2.153892	-5.106652	-0.443233
C	1.412036	2.855647	-2.072477
C	0.400326	3.899752	-1.574302
C	-0.866503	3.977714	-2.443490
C	-1.866874	5.031851	-1.958389
Sn	3.217861	2.683107	-0.789495

C	2.516455	2.331872	1.293595
C	3.558120	1.781775	2.275709
C	3.013495	1.604459	3.701760
C	4.049867	1.039854	4.678569
C	4.267216	4.646439	-0.863919
C	5.553746	4.745992	-0.035872
C	6.223546	6.127373	-0.112150
C	7.509919	6.223748	0.714364
H	-2.627236	-0.033639	-3.224123
H	-4.903387	0.619018	-2.454704
H	2.394660	-1.754557	-2.777974
H	0.114258	-0.931843	-3.346184
H	0.137597	-1.898012	3.148675
H	1.602203	-2.723066	4.986044
H	3.920387	-3.446250	4.512345
H	4.825576	-3.346679	2.195550
H	-7.404954	0.606467	2.734786
H	-6.546642	-0.061328	4.973494
H	-4.213526	-0.839136	5.242245
H	-2.694463	-0.974819	3.274021
H	-8.865122	-2.624317	-1.200319
H	-9.945223	1.488555	-1.672318
H	-6.885042	2.947329	-1.167569
H	-8.623438	3.191116	-1.017601
H	-7.645743	2.923991	0.423655
H	-6.926762	-3.292170	-0.254614
H	-5.525723	-2.267637	-0.589615
H	-6.256586	-2.254184	1.010072
H	-10.701720	-1.304033	-3.227616
H	-11.289903	-1.913022	-1.681559
H	-11.598093	-0.232740	-2.147824
H	7.034048	-3.990588	-2.440594
H	3.805133	-6.716734	-1.771075
H	1.365977	-4.499976	-0.900242
H	1.928071	-6.156641	-0.646599
H	2.088707	-4.952805	0.638037
H	6.190176	-1.541368	-0.198570
H	7.008967	-1.853465	-1.727079
H	5.446667	-1.038161	-1.716098
H	7.057441	-6.166545	-3.549546
H	5.630175	-7.213318	-3.491588
H	6.794564	-7.233627	-2.168122
H	3.964222	-1.451269	-0.125905
H	3.534298	5.390739	-0.523704
H	4.470695	4.868035	-1.918143
H	0.950804	1.863063	-2.115661
H	1.743998	3.095736	-3.089325
H	2.129424	3.297458	1.646164
H	1.658970	1.652546	1.226338
H	4.433846	2.445325	2.311833
H	3.931462	0.811949	1.918843
H	2.141398	0.938493	3.670044
H	2.647649	2.573241	4.068972
H	3.637901	0.945016	5.688876
H	4.932850	1.686886	4.740149
H	4.385612	0.045742	4.363473
H	0.868709	4.893631	-1.542171

H	0.102514	3.673293	-0.541206
H	-1.349497	2.991641	-2.458886
H	-0.576633	4.193916	-3.480808
H	-2.757530	5.062647	-2.594935
H	-1.421682	6.033498	-1.962036
H	-2.197547	4.822040	-0.934774
H	6.273924	3.985365	-0.368780
H	5.341857	4.515430	1.017631
H	5.510324	6.891293	0.226115
H	6.442120	6.361716	-1.162903
H	7.962506	7.218016	0.639275
H	8.254221	5.493791	0.375832
H	7.315427	6.026899	1.774936
H	-5.789808	1.703105	0.305325

Optimized structure of ***F**^{r}*

Atom	x	y	z
C	8.074606	-0.846438	-1.077437
C	6.880696	-0.479936	-0.416944
C	6.677398	0.875722	-0.065433
C	7.659748	1.818952	-0.380748
C	8.843562	1.474405	-1.037186
C	9.029843	0.133976	-1.372891
C	5.843497	-1.552202	-0.091859
C	4.452174	-1.404521	-0.703367
C	3.475496	-1.684006	0.275436
C	4.127809	-1.929890	1.562042
C	5.521148	-1.804871	1.378251
C	4.112135	-1.106175	-2.025508
C	2.770289	-1.080790	-2.413625
C	1.779167	-1.364521	-1.463183
C	2.148882	-1.660286	-0.137229
C	0.327549	-1.434503	-1.483252
C	-0.048729	-1.764890	-0.167063
O	1.047858	-1.904793	0.656956
C	-1.377772	-1.926234	0.206127
C	-2.350163	-1.733193	-0.797912
C	-2.003525	-1.406227	-2.111767
C	-0.659153	-1.258980	-2.464038
C	-2.036644	-2.284554	1.462733
C	-3.429169	-2.310377	1.235937
C	-3.744422	-1.873973	-0.191894
C	-4.302775	-2.648774	2.262278
C	-3.778761	-2.960770	3.522662
C	-2.398292	-2.927512	3.749195
C	-1.514017	-2.589501	2.722167
C	6.389360	-1.965690	2.450966
C	5.859022	-2.254917	3.713781
C	4.477937	-2.386318	3.894196
C	3.599187	-2.225410	2.820817
C	-4.779812	-2.683909	-0.968464
C	-4.573858	-4.051212	-1.269770
C	-5.552343	-4.749248	-1.983565
C	-6.736560	-4.146182	-2.413603
C	-6.922319	-2.797830	-2.110842
C	-5.971296	-2.057535	-1.397687
C	-3.324625	-4.793021	-0.851635

C	-6.271575	-0.597624	-1.117684
C	-7.787120	-4.935856	-3.157587
C	5.429640	1.349044	0.644858
C	9.872041	2.522600	-1.389658
C	8.375545	-2.276254	-1.484056
C	-0.770281	2.842977	-1.907314
Sn	-2.516209	3.003152	-0.542637
C	-3.041007	5.163104	-0.400231
C	-4.233304	5.501857	0.502189
C	-4.537224	7.006978	0.572381
C	-5.730000	7.343250	1.473132
C	0.485383	3.561635	-1.389002
C	1.698755	3.407406	-2.321920
C	2.946111	4.139286	-1.816377
C	-1.857723	2.296843	1.462867
C	-2.970926	1.925540	2.450364
C	-2.441347	1.490481	3.825862
C	-3.552865	1.106161	4.807587
H	2.499554	-0.845890	-3.438399
H	4.891344	-0.882567	-2.748024
H	-2.778945	-1.281354	-2.861525
H	-0.383275	-1.014908	-3.485238
H	-0.442798	-2.571225	2.893391
H	-2.009633	-3.172573	4.733267
H	-4.450069	-3.234596	4.331312
H	-5.374402	-2.683676	2.086536
H	7.461885	-1.857139	2.314992
H	6.526369	-2.377001	4.561952
H	4.084322	-2.611916	4.881031
H	2.527424	-2.321255	2.959666
H	9.945533	-0.163573	-1.878925
H	7.491419	2.856788	-0.101233
H	4.531787	1.175446	0.043434
H	5.491814	2.420520	0.852326
H	5.281521	0.830792	1.596917
H	9.350284	-2.334086	-1.974988
H	7.634682	-2.674103	-2.187266
H	8.404089	-2.957037	-0.625460
H	9.593510	3.061193	-2.304114
H	10.856017	2.076441	-1.560574
H	9.971766	3.268791	-0.594960
H	-7.833500	-2.301716	-2.437468
H	-5.379462	-5.799160	-2.210329
H	-2.427310	-4.366569	-1.310714
H	-3.385979	-5.842906	-1.149659
H	-3.175459	-4.760002	0.231706
H	-6.311274	-0.379377	-0.044221
H	-7.241391	-0.321853	-1.539474
H	-5.524418	0.074947	-1.555094
H	-8.452644	-4.280097	-3.726404
H	-7.333935	-5.646780	-3.855780
H	-8.411565	-5.516165	-2.466770
H	-4.131102	-0.850785	-0.109205
H	-2.133861	5.668995	-0.042306
H	-3.217012	5.523295	-1.420624
H	-0.570636	1.775757	-2.052054
H	-1.068631	3.245093	-2.882566

H	-1.234299	3.102412	1.873711
H	-1.196146	1.439654	1.293004
H	-3.655532	2.774592	2.587567
H	-3.582157	1.111981	2.035478
H	-1.761233	0.639071	3.693288
H	-1.837007	2.303803	4.250739
H	-3.144567	0.821307	5.783057
H	-4.246948	1.939359	4.969291
H	-4.132849	0.256439	4.430978
H	0.278088	4.632464	-1.252914
H	0.756281	3.178364	-0.395489
H	1.922600	2.339006	-2.442124
H	1.432188	3.779157	-3.320686
H	3.791267	4.011137	-2.500741
H	2.761244	5.214896	-1.713759
H	3.255171	3.762459	-0.834724
H	-5.131771	4.976006	0.149686
H	-4.051379	5.131828	1.520982
H	-3.644266	7.537121	0.930521
H	-4.724779	7.382036	-0.442965
H	-5.920799	8.421124	1.501883
H	-6.644528	6.853579	1.119172
H	-5.555920	7.009489	2.502541
H	6.232651	-2.502819	-0.476063

Optimized structure of ***Int1***

Atom	x	y	z
C	-6.959703	-0.838924	1.640225
C	-6.584374	-0.716461	0.283591
C	-7.420544	-1.243594	-0.726651
C	-8.607621	-1.884686	-0.361092
C	-8.995065	-2.019636	0.974935
C	-8.157976	-1.485830	1.958071
C	-5.324689	-0.023462	-0.082063
C	-4.031656	-0.531056	0.016136
C	-3.082134	0.475689	-0.440277
C	-3.856548	1.652927	-0.829954
C	-5.229628	1.334536	-0.607112
C	-3.567706	-1.808998	0.457299
C	-2.229085	-2.089892	0.463656
C	-1.274922	-1.114280	0.025445
C	-1.746696	0.175287	-0.428214
C	0.120844	-1.236032	-0.033774
C	0.766920	-0.042803	-0.534411
S	-0.407767	1.219982	-0.928741
C	2.129093	0.021758	-0.658123
C	2.928255	-1.135146	-0.276981
C	2.288866	-2.318208	0.207319
C	0.927210	-2.364479	0.327938
C	3.058127	1.051239	-1.119354
C	4.373360	0.502785	-1.007022
C	4.276611	-0.857041	-0.484325
C	-6.223718	2.277326	-0.875291
C	-5.850113	3.529870	-1.374319
C	-4.504577	3.838938	-1.597328
C	-3.497013	2.902123	-1.324290
C	2.872294	2.353158	-1.598763

C	4.027609	3.038110	-1.933976
C	5.321290	2.563579	-1.843687
C	5.497097	1.254238	-1.358897
C	5.424518	-1.764608	-0.240991
C	5.889616	-1.994631	1.074156
C	6.977816	-2.852528	1.264434
C	7.620548	-3.487167	0.198672
C	7.140270	-3.251011	-1.092171
C	6.057323	-2.401101	-1.333572
C	5.256691	-1.315995	2.267879
C	8.814715	-4.381763	0.432685
C	5.561173	-2.203243	-2.748455
C	-7.038432	-1.143096	-2.186064
C	-10.267862	-2.743286	1.345782
C	-6.101500	-0.265048	2.745514
C	4.280233	4.262509	1.739990
C	5.108681	3.191701	2.316966
N	5.760360	2.350918	2.782883
C	4.802451	5.643831	2.181410
C	2.797598	4.052286	2.104841
H	-1.874988	-3.059312	0.798350
H	-4.290355	-2.551355	0.782467
H	2.897956	-3.175944	0.475823
H	0.441445	-3.261650	0.697069
H	1.887047	2.797532	-1.704184
H	6.494003	0.835020	-1.255215
H	-7.268373	2.040161	-0.695611
H	-6.613671	4.271669	-1.589175
H	-4.234248	4.816585	-1.984726
H	-2.457230	3.161401	-1.499386
H	-9.245086	-2.291483	-1.143176
H	-8.446824	-1.568171	3.003652
H	-5.146885	-0.794559	2.835837
H	-6.613545	-0.332856	3.708985
H	-5.860755	0.787014	2.561146
H	-7.770131	-1.654930	-2.816558
H	-6.056668	-1.591308	-2.372789
H	-6.975952	-0.101593	-2.518666
H	-10.089158	-3.817914	1.477553
H	-11.030996	-2.635284	0.569143
H	-10.683633	-2.366531	2.285035
H	7.334600	-3.023918	2.277581
H	7.615770	-3.746273	-1.936069
H	4.500892	-2.462275	-2.840322
H	6.123734	-2.826601	-3.448391
H	5.658738	-1.161688	-3.072715
H	5.347799	-0.225932	2.211559
H	5.740233	-1.638054	3.193720
H	4.189495	-1.545848	2.349152
H	8.757409	-4.880067	1.405128
H	8.895889	-5.152355	-0.339866
H	9.749253	-3.806838	0.417856
H	2.650471	4.103984	3.187748
H	2.192243	4.834857	1.637849
H	2.437894	3.082826	1.752173
H	4.719395	5.763437	3.265772
H	5.849936	5.782104	1.901033

H	4.209883	6.429297	1.703015
H	4.382907	4.176351	0.650587
H	6.177370	3.167050	-2.130436

Table S20
Optimized structure of TS5

Atom	x	y	z
C	-7.404801	-1.094977	-0.946400
C	-6.604666	-0.742339	0.163763
C	-7.022343	-1.088441	1.468468
C	-8.227952	-1.776253	1.636208
C	-9.034358	-2.132316	0.551962
C	-8.601336	-1.784731	-0.730541
C	-5.330887	-0.012572	-0.040927
C	-4.018579	-0.565375	0.009404
C	-3.060330	0.477624	-0.241202
C	-3.806042	1.719361	-0.450965
C	-5.194465	1.399745	-0.326733
C	-3.595134	-1.894171	0.239081
C	-2.245338	-2.195446	0.233189
C	-1.283232	-1.186055	-0.005528
C	-1.715252	0.153859	-0.243813
C	0.148880	-1.317102	-0.049068
C	0.800539	-0.075658	-0.321353
S	-0.352502	1.249073	-0.524953
C	2.180300	-0.005633	-0.398399
C	2.944756	-1.198885	-0.200818
C	2.310739	-2.425530	0.067979
C	0.926499	-2.479500	0.141567
C	3.131729	1.080667	-0.644853
C	4.455954	0.493728	-0.597332
C	4.347705	-0.882835	-0.323483
C	5.590842	1.306971	-0.841722
C	5.419695	2.648144	-1.104686
C	4.126476	3.248541	-1.051990
C	2.970434	2.418093	-0.900421
C	-6.163473	2.399800	-0.466170
C	-5.751823	3.707065	-0.737673
C	-4.392577	4.016423	-0.863639
C	-3.410507	3.024627	-0.719048
C	5.470538	-1.841836	-0.167386
C	5.933935	-2.577439	-1.279129
C	6.994920	-3.471746	-1.107183
C	7.609909	-3.656627	0.133987
C	7.129380	-2.923755	1.222803
C	6.070489	-2.018757	1.099129
C	5.300858	-2.407451	-2.641832
C	5.571725	-1.263162	2.309721
C	8.777313	-4.602612	0.289804
C	-6.196379	-0.712442	2.678104
C	-10.348166	-2.847346	0.761600
C	-6.971406	-0.761757	-2.356167
C	4.143551	4.423669	0.996429
C	5.182479	5.479922	0.699520
C	2.713908	4.890663	1.131834

C	4.553495	3.358587	1.834257
N	4.909480	2.457412	2.489031
H	-1.916308	-3.214619	0.409916
H	-4.333049	-2.670928	0.415236
H	2.906624	-3.320402	0.218718
H	0.432862	-3.423521	0.350329
H	1.984204	2.866334	-0.967903
H	3.993467	4.237237	-1.479256
H	6.280909	3.271720	-1.323785
H	6.580914	0.860842	-0.844424
H	-7.217499	2.159216	-0.361037
H	-6.493465	4.492367	-0.850061
H	-4.091750	5.038420	-1.074009
H	-2.361213	3.286324	-0.816091
H	-9.207984	-2.062249	-1.589890
H	-8.545801	-2.037372	2.643322
H	-5.229224	-1.226306	2.685558
H	-6.720433	-0.969847	3.602283
H	-5.981586	0.361260	2.697217
H	-7.674317	-1.170499	-3.086797
H	-5.978607	-1.169030	-2.575585
H	-6.909333	0.319847	-2.516248
H	-10.326344	-3.470092	1.660919
H	-10.595284	-3.488841	-0.089643
H	-11.173464	-2.133996	0.880593
H	7.588381	-3.059994	2.199651
H	7.348819	-4.037462	-1.966430
H	4.233049	-2.651240	-2.623291
H	5.781893	-3.054859	-3.379760
H	5.380623	-1.373794	-2.995722
H	5.604680	-0.177999	2.168531
H	6.168847	-1.506873	3.192464
H	4.528586	-1.515623	2.531720
H	8.741800	-5.408257	-0.449625
H	9.732604	-4.079613	0.155402
H	8.795054	-5.056213	1.285472
H	2.613321	5.573946	1.987734
H	2.403115	5.445633	0.239740
H	2.024383	4.058854	1.287590
H	5.288721	6.165989	1.552302
H	6.163852	5.042664	0.503326
H	4.884634	6.083486	-0.164524

Optimized structure of ***Int2***

Atom	x	y	z
C	-7.048081	-1.138908	1.419286
C	-6.614920	-0.736419	0.135776
C	-7.409139	-1.027422	-0.996347
C	-8.613994	-1.714838	-0.823393
C	-9.058531	-2.124028	0.436755
C	-8.261457	-1.822025	1.544128
C	-5.335976	-0.005062	-0.021999
C	-4.020286	-0.572158	0.022292
C	-3.056250	0.471518	-0.173089
C	-3.790908	1.726098	-0.339681
C	-5.184475	1.413055	-0.246577
C	-3.610899	-1.909240	0.200831

C	-2.259484	-2.219847	0.197754
C	-1.291985	-1.210439	0.012247
C	-1.710092	0.137489	-0.174658
C	0.149290	-1.347962	-0.021762
C	0.805903	-0.104970	-0.235877
S	-0.336692	1.233577	-0.396054
C	2.191713	-0.042563	-0.300136
C	2.943070	-1.239268	-0.147392
C	2.307630	-2.467861	0.069940
C	0.917628	-2.516298	0.129693
C	3.154188	1.049302	-0.482777
C	4.490131	0.429798	-0.455371
C	4.373783	-0.920513	-0.251755
C	-6.143869	2.427830	-0.355783
C	-5.718438	3.741098	-0.566265
C	-4.354531	4.043608	-0.661573
C	-3.382270	3.038385	-0.546801
C	2.995117	2.376341	-0.640638
C	4.173616	3.312279	-0.755007
C	5.500115	2.595721	-0.846617
C	5.641735	1.265703	-0.680507
C	5.476526	-1.910049	-0.134300
C	6.171337	-2.047533	1.087784
C	7.200476	-2.990017	1.179000
C	7.562809	-3.798641	0.098245
C	6.857696	-3.650821	-1.099189
C	5.821344	-2.722327	-1.236254
C	4.172948	4.408156	0.404119
C	4.410814	3.738148	1.697796
N	4.602580	3.228900	2.723559
C	5.808168	-1.209716	2.293386
C	8.700176	-4.785954	0.213993
C	5.096244	-2.590784	-2.556544
C	-6.964789	-0.624761	-2.384462
C	-10.348995	-2.892975	0.593977
C	-6.234535	-0.818642	2.653065
C	5.296758	5.446216	0.171850
C	2.817328	5.150924	0.468976
H	-1.940143	-3.248159	0.336037
H	-4.355202	-2.688091	0.336099
H	2.896409	-3.371385	0.193962
H	0.419113	-3.466106	0.298218
H	2.003613	2.815524	-0.683288
H	4.038806	3.916206	-1.668833
H	6.374929	3.203830	-1.052658
H	6.623046	0.802954	-0.739773
H	-7.201043	2.192080	-0.273627
H	-6.451891	4.537105	-0.654307
H	-4.042811	5.071115	-0.823871
H	-2.329784	3.295423	-0.618289
H	-9.219343	-1.938693	-1.699168
H	-8.594559	-2.120219	2.535905
H	-5.265984	-1.329792	2.644693
H	-6.766125	-1.121044	3.559129
H	-6.023023	0.253504	2.725186
H	-7.660720	-0.998716	-3.140003
H	-5.969447	-1.019543	-2.614850

H	-6.903352	0.463385	-2.491542
H	-10.178810	-3.974126	0.514538
H	-11.073730	-2.621851	-0.179605
H	-10.808776	-2.708538	1.569523
H	7.729414	-3.097273	2.123529
H	7.120293	-4.273594	-1.951698
H	4.026686	-2.803818	-2.453869
H	5.507455	-3.280307	-3.298484
H	5.176231	-1.574410	-2.957166
H	5.977930	-0.142086	2.119767
H	6.398846	-1.506147	3.164071
H	4.749097	-1.316812	2.551008
H	8.791052	-5.174412	1.232875
H	8.563979	-5.635268	-0.462194
H	9.659469	-4.318121	-0.041114
H	2.868328	5.960845	1.201386
H	2.588505	5.588558	-0.508520
H	1.998561	4.490611	0.758883
H	5.250241	6.227095	0.935437
H	6.289312	4.995513	0.215652
H	5.166624	5.917491	-0.807953

Optimized structure of ***TS6***

Atom	x	y	z
C	-7.782142	-0.587918	-1.249099
C	-7.051697	-0.637308	-0.040352
C	-7.601643	-1.287693	1.087172
C	-8.867466	-1.872072	0.983856
C	-9.606761	-1.832864	-0.201339
C	-9.042919	-1.188861	-1.306103
C	-5.712124	-0.009293	0.043492
C	-4.454357	-0.684406	0.013735
C	-3.400261	0.285858	0.123186
C	-4.024638	1.605935	0.227968
C	-5.439633	1.405074	0.174530
C	-4.160377	-2.059474	-0.115556
C	-2.842194	-2.481515	-0.128124
C	-1.787713	-1.547043	-0.017355
C	-2.089295	-0.158422	0.107311
C	-0.368902	-1.802274	-0.016203
C	0.397375	-0.605937	0.109612
S	-0.624551	0.830009	0.227566
C	1.782288	-0.656220	0.126506
C	2.429905	-1.922511	0.010360
C	1.682835	-3.106531	-0.106632
C	0.295919	-3.041313	-0.120731
C	2.835743	0.357208	0.242121
C	4.105332	-0.358364	0.184861
C	3.868626	-1.723756	0.048024
C	5.316643	0.385686	0.288864
C	5.278572	1.740945	0.435089
C	4.024054	2.523375	0.424873
C	2.790775	1.706088	0.396825
C	-6.311083	2.496653	0.265200
C	-5.775137	3.780032	0.397835
C	-4.389867	3.974219	0.445384
C	-3.505201	2.887716	0.362852

C	4.887528	-2.802127	-0.029299
C	5.239956	-3.354157	-1.280421
C	6.196112	-4.372801	-1.329220
C	6.814035	-4.862212	-0.175159
C	6.445679	-4.307064	1.053229
C	5.495461	-3.285733	1.150340
C	4.608998	-2.851516	-2.559365
C	5.123449	-2.730335	2.506786
C	7.867142	-5.941920	-0.255473
C	4.001421	3.742383	1.432598
C	5.163399	4.733467	1.175557
C	-6.852308	-1.343288	2.399518
C	-10.985655	-2.444287	-0.278232
C	-7.208939	0.077645	-2.479931
C	2.668812	4.530072	1.386920
C	4.160603	3.187139	2.795383
N	4.285575	2.770107	3.872169
C	4.056609	3.613420	-2.222043
C	3.062680	2.645471	-2.830089
C	3.636135	4.968590	-2.133950
N	3.284703	6.077226	-2.001856
C	5.518339	3.378547	-2.538011
H	-2.612023	-3.537702	-0.226778
H	-4.970204	-2.776706	-0.207999
H	2.190679	-4.062909	-0.183463
H	-0.285947	-3.953243	-0.211996
H	1.837610	2.220094	0.450159
H	4.013387	3.106255	-0.691238
H	6.211845	2.281900	0.544791
H	6.268188	-0.138572	0.277365
H	-7.385933	2.342920	0.235785
H	-6.439400	4.636206	0.467470
H	-3.991622	4.978888	0.550116
H	-2.434304	3.060911	0.407234
H	-9.596237	-1.156606	-2.242175
H	-9.287464	-2.368299	1.856072
H	-5.931209	-1.930131	2.317467
H	-7.469219	-1.794790	3.180808
H	-6.558714	-0.342937	2.734872
H	-7.877849	-0.044340	-3.335814
H	-6.235313	-0.347004	-2.747236
H	-7.051942	1.150365	-2.325186
H	-11.086655	-3.292157	0.405824
H	-11.212509	-2.794877	-1.289537
H	-11.758605	-1.714260	-0.006817
H	6.906031	-4.681487	1.964891
H	6.464743	-4.793809	-2.295662
H	3.524598	-3.005574	-2.564378
H	5.026164	-3.365991	-3.428888
H	4.774299	-1.776510	-2.690941
H	5.409282	-1.678024	2.608956
H	5.618254	-3.289754	3.304795
H	4.043038	-2.779263	2.678209
H	7.702328	-6.599410	-1.114287
H	8.869946	-5.510089	-0.364661
H	7.877373	-6.559148	0.647807
H	2.732191	5.387134	2.062187

H	2.486490	4.910189	0.378796
H	1.819227	3.924279	1.706676
H	5.142550	5.523741	1.930175
H	6.142604	4.254490	1.229873
H	5.044072	5.201951	0.196005
H	5.690463	3.400156	-3.623077
H	6.162002	4.134845	-2.082329
H	5.829147	2.392105	-2.175820
H	3.032698	2.756567	-3.923028
H	3.352189	1.614041	-2.609554
H	2.049928	2.807230	-2.452512

Optimized structure of ***Int3***

Atom	x	y	z
C	5.768541	-2.783501	-1.217389
C	5.426435	-2.112527	-0.021937
C	6.138797	-2.387755	1.167218
C	7.174283	-3.326146	1.137236
C	7.531567	-3.997221	-0.035480
C	6.813623	-3.712066	-1.199861
C	4.325145	-1.120128	-0.014160
C	2.935585	-1.395613	-0.015870
C	2.205125	-0.151483	-0.009352
C	3.189248	0.931784	-0.007240
C	4.480585	0.321536	-0.007992
C	2.247090	-2.634858	-0.008173
C	0.868414	-2.654258	-0.004549
C	0.127346	-1.443048	-0.002967
C	0.825723	-0.191859	-0.003539
S	-0.285595	1.184367	0.005656
C	-1.683897	0.099826	0.009367
C	-1.290838	-1.278259	0.003214
C	-3.016712	0.456770	0.016005
C	-4.011994	-0.587646	0.017545
C	-3.626825	-1.951666	0.004839
C	-2.289435	-2.287502	0.000437
C	-3.725404	1.737517	0.019690
C	-5.122944	1.439991	0.019054
C	-5.300733	-0.000108	0.018114
C	-6.064311	2.473667	0.035455
C	-5.614425	3.797206	0.042557
C	-4.245609	4.086494	0.038676
C	-3.291177	3.057803	0.029207
C	5.622882	1.123141	-0.020042
C	5.482918	2.514299	-0.020632
C	4.219409	3.122825	-0.015099
C	3.064396	2.313191	-0.010712
C	-6.599236	-0.715697	0.022463
C	-7.093157	-1.281217	1.219273
C	-8.324351	-1.943492	1.200129
C	-9.080355	-2.067569	0.031499
C	-8.575541	-1.498225	-1.140790
C	-7.351065	-0.824607	-1.169078
C	-6.324550	-1.160108	2.516034
C	-6.841324	-0.242606	-2.468182
C	-10.391678	-2.816961	0.029356
C	4.045688	4.653748	-0.017493

C	3.286938	5.124265	1.253554
C	5.779123	-1.705138	2.467590
C	8.677830	-4.980522	-0.049634
C	5.036910	-2.500410	-2.510346
C	5.360231	5.328647	-0.016976
N	6.374333	5.894192	-0.016534
C	3.289620	5.119975	-1.291783
H	-1.990865	-3.330854	-0.009045
H	-4.390292	-2.723597	-0.004689
H	2.812656	-3.561616	-0.001467
H	0.338473	-3.601357	0.001148
H	2.080555	2.771563	-0.011873
H	6.373286	3.133994	-0.027600
H	6.611506	0.673765	-0.031727
H	-7.126622	2.247404	0.045713
H	-6.334547	4.609951	0.053588
H	-3.915198	5.120829	0.045520
H	-2.233524	3.303225	0.031321
H	-9.148543	-1.581429	-2.061763
H	-8.704492	-2.368412	2.126596
H	-5.373914	-1.702686	2.476818
H	-6.905102	-1.561084	3.350960
H	-6.081586	-0.116385	2.741643
H	-7.520959	-0.474522	-3.292285
H	-5.852471	-0.639212	-2.722369
H	-6.738013	0.846206	-2.412241
H	-10.241213	-3.878666	-0.203523
H	-11.081016	-2.416062	-0.719802
H	-10.883644	-2.765985	1.005238
H	7.712621	-3.541070	2.057767
H	7.075261	-4.222932	-2.123945
H	3.988729	-2.814470	-2.462300
H	5.506385	-3.026649	-3.345530
H	5.034196	-1.430163	-2.742399
H	5.935669	-0.622529	2.414225
H	6.385065	-2.091831	3.291035
H	4.724659	-1.858333	2.721221
H	8.787572	-5.481651	0.916790
H	8.537774	-5.748230	-0.816423
H	9.628941	-4.477025	-0.263814
H	3.185144	6.213157	1.257673
H	2.286781	4.684570	1.276487
H	3.818294	4.819299	2.158093
H	3.186919	6.208777	-1.299458
H	3.823408	4.812665	-2.194089
H	2.289942	4.679200	-1.315674

Optimized structure of ***TS7***

Atom	x	y	z
C	-6.826773	-2.361398	-0.889234
C	-6.040639	-2.023234	0.235416
C	-6.387794	-2.522237	1.511095
C	-7.509648	-3.347100	1.635592
C	-8.296740	-3.697870	0.535502
C	-7.937976	-3.191641	-0.716751
C	-4.861056	-1.139904	0.078306
C	-3.493835	-1.541686	0.069762

C	-2.662251	-0.380671	-0.102584
C	-3.547318	0.780735	-0.201522
C	-4.887685	0.296456	-0.090699
C	-2.919832	-2.828353	0.183616
C	-1.545305	-2.973264	0.139278
C	-0.706256	-1.845764	-0.023630
C	-1.289569	-0.548384	-0.144875
C	0.730401	-1.810970	-0.092211
C	1.235459	-0.486384	-0.265405
S	-0.062586	0.712486	-0.345905
C	2.596951	-0.255535	-0.350048
C	3.492152	-1.368477	-0.262337
C	3.003239	-2.676370	-0.091946
C	1.635337	-2.890894	-0.008463
C	3.418143	0.946792	-0.513562
C	4.799407	0.508593	-0.531246
C	4.848967	-0.889363	-0.373726
C	5.833175	1.460225	-0.717184
C	5.510865	2.791526	-0.861802
C	4.161071	3.238228	-0.743064
C	3.106300	2.274911	-0.651070
C	-5.953473	1.197907	-0.137846
C	-5.691309	2.560463	-0.304519
C	-4.379583	3.044261	-0.420077
C	-3.301772	2.136055	-0.364222
C	6.073354	-1.727033	-0.310954
C	6.605054	-2.294510	-1.488663
C	7.760903	-3.076643	-1.403333
C	8.404768	-3.310904	-0.185343
C	7.856064	-2.745558	0.969179
C	6.701579	-1.957332	0.933043
C	5.942881	-2.066580	-2.828918
C	6.134451	-1.381612	2.210622
C	9.671943	-4.130665	-0.121054
C	-5.583404	-2.157254	2.738581
C	-9.484623	-4.617561	0.690774
C	-6.472005	-1.856310	-2.269640
C	4.094314	4.228501	1.400199
C	5.025680	5.399998	1.193026
C	2.628787	4.544391	1.580959
C	4.611734	3.137380	2.139196
N	5.056821	2.219079	2.710229
H	-1.101187	-3.959769	0.227020
H	-3.562756	-3.695223	0.301470
H	3.697449	-3.508247	-0.024403
H	1.253405	-3.898241	0.124658
H	2.075732	2.615313	-0.667981
H	3.912127	4.239118	-1.080781
H	6.293795	3.522962	-1.036296
H	6.865853	1.128381	-0.768890
H	-6.976627	0.846240	-0.043242
H	-6.522084	3.256982	-0.343111
C	-4.071024	4.542418	-0.604266
H	-2.282070	2.498621	-0.447391
H	-8.537524	-3.451022	-1.586610
H	-7.778352	-3.719694	2.621659
H	-4.564814	-2.556931	2.690876

H	-6.055307	-2.549299	3.643262
H	-5.490365	-1.071534	2.847958
H	-7.138892	-2.282691	-3.023468
H	-5.443461	-2.119315	-2.539007
H	-6.545072	-0.765471	-2.334455
H	-9.193595	-5.667050	0.556739
H	-10.259596	-4.399481	-0.050123
H	-9.931557	-4.529836	1.685509
H	8.337222	-2.923127	1.928542
H	8.167154	-3.513452	-2.313102
H	4.907759	-2.425132	-2.833005
H	6.482777	-2.586142	-3.624881
H	5.907389	-1.001823	-3.084058
H	6.039033	-0.291664	2.171551
H	6.767799	-1.634623	3.065002
H	5.130993	-1.774269	2.410919
H	9.750147	-4.673354	0.825905
H	9.719245	-4.859993	-0.935221
H	10.561366	-3.493251	-0.203210
H	2.474538	5.138621	2.493423
H	2.256910	5.142576	0.741643
H	2.022987	3.640273	1.666542
H	5.075101	6.017532	2.101507
H	6.041847	5.076118	0.958243
H	4.663605	6.044225	0.384658
C	-5.318668	5.333291	-0.641552
C	-3.222046	5.079425	0.580464
C	-3.329477	4.790908	-1.946325
H	-3.022384	6.147527	0.455782
H	-3.741631	4.929294	1.529663
H	-2.265957	4.551873	0.624097
H	-3.130473	5.857710	-2.082531
H	-2.375284	4.258202	-1.950550
H	-3.925413	4.436002	-2.790346
N	-6.276245	5.989102	-0.675758

Optimized structure of ***Int4***

Atom	x	y	z
C	-6.833136	-2.331727	-0.936276
C	-6.055227	-2.020907	0.201921
C	-6.415115	-2.545989	1.463678
C	-7.541136	-3.368677	1.561031
C	-8.320314	-3.692471	0.447057
C	-7.949019	-3.160935	-0.790944
C	-4.870337	-1.140770	0.074000
C	-3.497779	-1.554076	0.072081
C	-2.660668	-0.398100	-0.069992
C	-3.536865	0.770902	-0.155610
C	-4.883227	0.295097	-0.068325
C	-2.936336	-2.843791	0.166827
C	-1.558816	-2.996991	0.132668
C	-0.714559	-1.874897	-0.000935
C	-1.285385	-0.574927	-0.103224
C	0.732186	-1.845726	-0.055591
C	1.242011	-0.526245	-0.199614
S	-0.047787	0.680970	-0.270054
C	2.610688	-0.303229	-0.270396

C	3.493547	-1.414771	-0.196507
C	3.003424	-2.718025	-0.049338
C	1.628903	-2.926815	0.018651
C	3.442733	0.899087	-0.391425
C	4.839725	0.432903	-0.411847
C	4.877991	-0.931726	-0.291258
C	5.887814	1.404831	-0.592113
C	5.596668	2.718372	-0.671271
C	4.200385	3.274711	-0.522267
C	3.134882	2.207229	-0.463200
C	-5.941749	1.207190	-0.109570
C	-5.667100	2.569813	-0.246907
C	-4.349704	3.044936	-0.338500
C	-3.279649	2.127187	-0.288768
C	6.085596	-1.796728	-0.242407
C	6.503641	-2.496928	-1.394798
C	7.638367	-3.310534	-1.322034
C	8.371434	-3.451099	-0.140596
C	7.936056	-2.755001	0.990392
C	6.807508	-1.929541	0.964408
C	5.751186	-2.367140	-2.699888
C	6.369430	-1.212892	2.222131
C	9.612725	-4.310545	-0.093429
C	-4.027398	4.543873	-0.488149
C	-3.264161	4.813557	-1.813877
C	4.094794	4.282365	0.709887
C	5.098377	5.448884	0.547159
C	-5.620196	-2.209407	2.705309
C	-9.512994	-4.610481	0.572689
C	-6.464584	-1.798926	-2.302611
C	2.668185	4.868897	0.829835
C	4.415574	3.553981	1.953067
N	4.669799	2.997435	2.939922
C	-5.268219	5.345121	-0.528360
N	-6.219897	6.009339	-0.563904
C	-3.192174	5.050365	0.719628
H	-1.122848	-3.988419	0.206392
H	-3.585262	-3.708941	0.262228
H	3.691937	-3.554746	0.014984
H	1.242775	-3.935126	0.133444
H	2.100141	2.534539	-0.470464
H	3.986697	3.920086	-1.391543
H	6.394968	3.432490	-0.845087
H	6.913385	1.059299	-0.688830
H	-6.968580	0.862000	-0.032479
H	-6.491583	3.273989	-0.280907
H	-2.256331	2.483393	-0.352713
H	-8.542157	-3.399230	-1.671169
H	-7.819646	-3.761188	2.536600
H	-4.599972	-2.604414	2.654430
H	-6.096853	-2.625117	3.596829
H	-5.531550	-1.126401	2.842179
H	-7.125107	-2.208495	-3.071234
H	-5.434016	-2.058340	-2.567761
H	-6.535189	-0.706875	-2.345709
H	-9.223787	-5.658636	0.424930
H	-10.278190	-4.377483	-0.173784

H	-9.971685	-4.537669	1.563288
H	8.486219	-2.859664	1.922979
H	7.957132	-3.847165	-2.213061
H	4.712932	-2.702861	-2.603857
H	6.225871	-2.961219	-3.485175
H	5.713951	-1.325993	-3.038374
H	6.412639	-0.124311	2.113116
H	7.003725	-1.490746	3.067858
H	5.333901	-1.457143	2.482122
H	9.759630	-4.747644	0.898835
H	9.562715	-5.126982	-0.820055
H	10.510591	-3.723809	-0.325182
H	2.639262	5.626357	1.617471
H	2.384744	5.346882	-0.113912
H	1.927969	4.105414	1.074402
H	4.975216	6.166567	1.362466
H	6.133881	5.105762	0.558460
H	4.909681	5.968837	-0.397873
H	-3.055036	5.881314	-1.925276
H	-2.314149	4.273425	-1.814986
H	-3.850183	4.480687	-2.673699
H	-2.982219	6.119073	0.619377
H	-3.727116	4.885757	1.657812
H	-2.241139	4.514095	0.767306

Optimized structure of ***TS8***

Atom	x	y	z
C	-7.031658	-2.588706	1.055565
C	-6.551361	-1.912458	-0.088623
C	-7.278750	-1.973434	-1.298842
C	-8.465824	-2.710176	-1.341123
C	-8.956893	-3.388227	-0.222097
C	-8.225527	-3.310427	0.966154
C	-5.293082	-1.133405	-0.017702
C	-3.965610	-1.660281	-0.015525
C	-3.029073	-0.573614	0.065925
C	-3.801812	0.668999	0.119339
C	-5.183182	0.305412	0.064813
C	-3.515675	-2.996344	-0.095403
C	-2.158003	-3.265333	-0.085849
C	-1.217283	-2.213998	-0.001355
C	-1.675861	-0.865591	0.073548
C	0.221476	-2.306191	0.016018
C	0.846284	-1.027528	0.105005
S	-0.333771	0.286208	0.168404
C	2.227542	-0.918930	0.129795
C	3.015730	-2.106269	0.057942
C	2.408849	-3.370655	-0.021885
C	1.023576	-3.464273	-0.043674
C	3.157924	0.211026	0.214848
C	4.501224	-0.356537	0.183987
C	4.422570	-1.743613	0.092722
C	5.619410	0.524168	0.265266
C	5.426173	1.870238	0.365243
C	4.090316	2.502985	0.324187
C	2.958504	1.549985	0.324700
C	-6.161688	1.301221	0.112961

C	-5.771911	2.640174	0.201651
C	-4.418667	3.007221	0.249510
C	-3.429531	2.001702	0.209999
C	5.557891	-2.700870	0.053998
C	6.216088	-3.065350	1.249436
C	7.281950	-3.968375	1.187594
C	7.714197	-4.523242	-0.019953
C	7.050074	-4.147498	-1.190817
C	5.978750	-3.249538	-1.177325
C	5.782170	-2.504148	2.585007
C	5.303295	-2.863811	-2.474049
C	8.847404	-5.521139	-0.054850
C	-3.970836	4.478195	0.347759
C	-3.171683	4.723989	1.656894
C	3.925620	3.749476	1.283906
C	2.502254	4.357980	1.236392
C	-6.782419	-1.274513	-2.544525
C	-10.226779	-4.201799	-0.301658
C	-6.292126	-2.519302	2.372866
C	4.175053	3.276006	2.663871
N	4.366215	2.922956	3.753718
C	4.945587	4.869571	0.962994
C	-3.111411	4.876183	-0.883458
C	-5.142234	5.378499	0.373991
N	-6.039552	6.115025	0.396909
C	4.014252	3.483623	-2.362738
C	3.328721	4.728574	-2.332419
N	2.754747	5.744830	-2.243973
C	5.501798	3.531479	-2.638695
C	3.249876	2.316432	-2.952644
H	-1.808477	-4.291153	-0.146812
H	-4.237788	-3.803761	-0.167806
H	3.022637	-4.264975	-0.064768
H	0.549956	-4.439035	-0.106776
H	1.952775	1.953695	0.358111
H	4.018215	3.034032	-0.814645
H	6.290642	2.517660	0.462277
H	6.624450	0.111715	0.274805
H	-7.215350	1.039669	0.084689
H	-6.534572	3.410756	0.236100
H	-2.379603	2.273480	0.252266
H	-9.019948	-2.756299	-2.276067
H	-8.596587	-3.819764	1.852819
H	-5.309417	-2.999245	2.313647
H	-6.860121	-3.015522	3.163982
H	-6.118057	-1.482628	2.680159
H	-7.426349	-1.498200	-3.398923
H	-5.763147	-1.585353	-2.797906
H	-6.757606	-0.186985	-2.418058
H	-10.739334	-4.239388	0.664148
H	-10.016144	-5.237058	-0.598199
H	-10.921510	-3.788758	-1.039019
H	7.785785	-4.246609	2.110604
H	7.375558	-4.560979	-2.142889
H	4.243345	-3.139808	-2.476769
H	5.781305	-3.356431	-3.324666
H	5.346942	-1.781964	-2.641694

H	5.952666	-1.424133	2.649269
H	6.331847	-2.977749	3.402380
H	4.712869	-2.663348	2.758938
H	8.477129	-6.545669	0.076225
H	9.380394	-5.488860	-1.009733
H	9.570639	-5.333864	0.744427
H	2.468344	5.247156	1.871091
H	2.252682	4.662869	0.217114
H	1.745062	3.663734	1.605130
H	4.847839	5.674631	1.695576
H	5.978516	4.518595	0.997007
H	4.739185	5.287692	-0.024896
H	5.696382	3.550420	-3.719972
H	5.972781	4.413639	-2.198763
H	5.988290	2.636544	-2.234312
H	3.216528	2.389641	-4.048636
H	3.741797	1.373319	-2.698021
H	2.219285	2.276608	-2.591238
H	-2.814428	5.926597	-0.819899
H	-2.206045	4.265483	-0.921900
H	-3.668532	4.725915	-1.811118
H	-2.874327	5.773636	1.731818
H	-3.771694	4.466761	2.532873
H	-2.268442	4.108915	1.664930

Optimized structure of ***1***

Atom	x	y	z
C	6.794406	-2.196738	1.169587
C	6.053630	-2.008340	-0.019074
C	6.472110	-2.633521	-1.215111
C	7.618768	-3.433180	-1.197851
C	8.361947	-3.637342	-0.032226
C	7.932571	-3.007489	1.139180
C	4.847043	-1.147237	-0.012598
C	3.498028	-1.579984	-0.014474
C	2.629757	-0.427742	-0.010472
C	3.483690	0.761256	-0.010054
C	4.836307	0.302695	-0.009058
C	2.957041	-2.890080	-0.004831
C	1.589789	-3.068182	-0.001382
C	0.714538	-1.950294	-0.002103
C	1.264337	-0.627234	-0.005031
C	-0.713305	-1.950559	0.003564
C	-1.263596	-0.627709	0.006715
S	0.000138	0.611194	0.000977
C	-2.629093	-0.428731	0.011994
C	-3.496948	-1.581291	0.015673
C	-2.955464	-2.891190	0.005770
C	-1.588143	-3.068770	0.002447
C	-3.483440	0.759968	0.011263
C	-4.835886	0.300910	0.009736
C	-4.846116	-1.149014	0.013540
C	-5.879631	1.227060	0.022189
C	-5.582318	2.593279	0.025582
C	-4.257875	3.053980	0.022476
C	-3.202406	2.118119	0.017568
C	5.879700	1.229243	-0.022305

C	5.581862	2.595351	-0.025837
C	4.257249	3.055556	-0.022079
C	3.202139	2.119298	-0.016465
C	-6.052877	-2.009871	0.019009
C	-6.469843	-2.639351	1.213242
C	-7.613002	-3.444023	1.193071
C	-8.356913	-3.644952	0.027422
C	-7.924717	-3.016900	-1.143978
C	-6.790119	-2.201145	-1.171466
C	-5.712731	-2.442170	2.507489
C	-6.354133	-1.561779	-2.470523
C	-9.606547	-4.492973	0.038570
C	-3.911338	4.555246	0.028027
C	-3.108433	4.930027	1.303798
C	5.717093	-2.430580	-2.509684
C	9.578392	-4.532400	-0.032188
C	6.363147	-1.553429	2.468278
C	-3.103172	4.938728	-1.241779
C	-5.140608	5.375132	0.027978
N	-6.084099	6.051865	0.027950
H	1.172405	-4.069924	0.005890
H	3.625748	-3.745333	0.003395
H	-3.623816	-3.746721	-0.002917
H	-1.170397	-4.070359	-0.005097
H	-2.172900	2.461918	0.020579
H	-6.396311	3.310308	0.032873
H	-6.912911	0.892944	0.031923
H	6.913115	0.895550	-0.032642
H	6.395589	3.312676	-0.033752
C	3.910132	4.556685	-0.027775
H	2.172501	2.462706	-0.019080
H	8.497181	-3.151489	2.057767
H	7.942631	-3.903525	-2.123691
H	4.710262	-2.859465	-2.464919
H	6.244120	-2.899969	-3.344361
H	5.596062	-1.366878	-2.740406
H	7.008058	-1.869385	3.292251
H	5.331962	-1.820499	2.723280
H	6.400480	-0.460289	2.412316
H	9.308287	-5.568011	0.209878
H	10.314267	-4.208078	0.709658
H	10.066153	-4.545090	-1.011357
H	-8.482560	-3.168701	-2.065438
H	-7.931868	-3.923269	2.116078
H	-4.706427	-2.872026	2.460255
H	-6.239424	-2.913594	3.341230
H	-5.590263	-1.379369	2.741535
H	-6.390568	-0.468483	-2.417036
H	-6.997132	-1.878835	-3.295567
H	-5.322492	-1.830388	-2.722070
H	-9.766061	-4.983801	-0.926200
H	-9.558462	-5.267277	0.809921
H	-10.496140	-3.884345	0.243822
H	-2.877461	6.008824	-1.243457
H	-2.160075	4.387010	-1.265823
H	-3.665492	4.698731	-2.147133
H	-2.882051	5.999948	1.313499

H	-3.674816	4.684397	2.205093
H	-2.165846	4.377424	1.328200
C	3.106731	4.930926	-1.303395
C	3.102162	4.940072	1.242183
C	5.139071	5.377065	-0.028204
H	2.879906	6.000752	-1.313213
H	3.672978	4.685379	-2.204798
H	2.164368	4.377923	-1.327452
H	2.876056	6.010085	1.243740
H	2.159271	4.388015	1.266577
H	3.664815	4.700438	2.147425
N	6.082259	6.054220	-0.028644

5. References

1. S. Mori, M. Akita, S. Suzuki, M. S. Asano, M. Murata, T. Akiyama, T. Matsumoto, C. Kitamura, S.-i. Kato, *Chem. Commun.* **2020**, *56*, 5881.
2. S. Mori, S. Moles Quintero, N. Tabaka, R. Kishi, R. González Núñez, A. Harbuzaru, R. Ponce Ortiz, J. Marín-Beloqui, S. Suzuki, C. Kitamura, C. J. Gómez-García, Y. Dai, F. Negri, M. Nakano, S.-i. Kato, J. Casado, *Angew. Chem. Int. Ed.* **2022**, *61*, e202206680.
3. X. Fu, D. Zhao, *Org. Lett.* **2015**, *17*, 5694.
4. *CrysAlisPro*; Rigaku OD, The Woodlands, TX, 2015.
5. O. V. Dolomanov, L. J. Bourhis, R. J. Gildea, J. A. K. Howard, H. Puschmann, *J. Appl. Cryst.* **2009**, *42*, 339.
6. G. M. Sheldrick, *Acta Crystallogr.*, *Sect. A* **2015**, *71*, 3.
7. G. M. Sheldrick, *Acta Crystallogr., Sect. C* **2015**, *71*, 3.
8. Yamaguchi, K. *Chem. Phys. Lett.* **1975**, *33*, 330.
9. A. D. Becke, *J. Chem. Phys.* **1993**, *98*, 5648.
10. C. Lee, W. Yang, R. G. Parr, *Phys. Rev. B* **1998**, *57*, 785.
11. R. Ditchfield, W. J. Hehre, J. A. Pople, *J. Chem. Phys.* **1971**, *54*, 724.
12. *Gaussian 16*, Revision C.01, M. J. Frisch, G. W. Trucks, H. B. Schlegel, G. E. Scuseria, M. A. Robb, J. R. Cheeseman, G. Scalmani, V. Barone, G. A. Petersson, H. Nakatsuji, X. Li, M. Caricato, A. V. Marenich, J. Bloino, B. G. Janesko, R. Gomperts, B. Mennucci, H. P. Hratchian, J. V. Ortiz, A. F. Izmaylov, J. L. Sonnenberg, D. Williams-Young, F. Ding, F. Lipparini, F. Egidi, J. Goings, B. Peng, A. Petrone, T. Henderson, D. Ranasinghe, V. G. Zakrzewski, J. Gao, N. Rega, G. Zheng, W. Liang, M. Hada, M. Ehara, K. Toyota, R. Fukuda, J. Hasegawa, M. Ishida, T. Nakajima, Y. Honda, O. Kitao, H. Nakai, T. Vreven, K. Throssell, J. A. Montgomery, Jr., J. E. Peralta, F. Ogliaro, M. J. Bearpark, J. J. Heyd, E. N. Brothers, K. N. Kudin, V. N. Staroverov, T. A. Keith, R. Kobayashi, J. Normand, K. Raghavachari, A. P. Rendell, J. C. Burant, S. S. Iyengar, J. Tomasi, M. Cossi, J. M. Millam, M. Klene, C. Adamo, R. Cammi, J. W. Ochterski, R. L. Martin, K. Morokuma, O. Farkas, J. B. Foresman, and D. J. Fox, Gaussian, Inc., Wallingford CT, 2016.
